# Exploring the efficacy of identity priming and message framing in influencing American attitudes toward trophy hunting

**DOI:** 10.1371/journal.pone.0312949

**Published:** 2024-11-07

**Authors:** Eden Rozing, Elena C. Rubino, Amber Turner, Pipiet Larasatie

**Affiliations:** 1 Division of Agriculture, Arkansas Forest Resource Center, University of Arkansas System, Monticello, Arkansas, United States of America; 2 College of Forestry, Agriculture, and Natural Resources, University of Arkansas at Monticello, Monticello, Arkansas, United States of America; 3 Arkansas Center for Forest Business, University of Arkansas at Monticello, Monticello, Arkansas, United States of America; 4 School of Social and Behavioral Sciences, University of Arkansas at Monticello, Monticello, Arkansas, United States of America; Spanish Scientific Research Council, SPAIN

## Abstract

In the United States, the general public typically disapproves of trophy hunting. Given the global ubiquity of the practice, its potential to benefit conservation when properly managed, and the substantial role played by American hunter-tourists, changing these attitudes can help to maintain the practice as a wildlife management tool. Existing trophy hunting communications, which are currently neither well-designed nor widely distributed, are unable to effectively do so. We used an online survey to explore current American attitudes regarding trophy hunting and assess the efficacy of different messaging strategies in influencing these attitudes. Respondents were randomly sorted into nine treatment groups, each of which received both an identity-focused priming item (or lack thereof, as a control) and a message about trophy hunting. The priming items prompted respondents to consider either their personal values (personal identity) or political affiliation (social identity) before reading their assigned message. All three messages evaluated in this study contained a brief informative paragraph about trophy hunting, and additional information concerning either the ecological or socioeconomic benefits associated with the practice was appended onto this paragraph for respondents assigned to treatments featuring an experimental message frame. Based on the responses of 2000 U.S. residents, we found that negative attitudes regarding trophy hunting were pervasive and resistant to change. Messages framed around the ecological or socioeconomic benefits of managed trophy hunting slightly increased approval for the practice, but identity-focused priming items had no comparable effect. Additionally, respondents’ trust in the messages varied by source. This research represents a novel approach to understanding and changing public attitudes toward a highly controversial form of hunting through scientifically informed messaging. While our findings suggest several areas of improvement specifically for future trophy hunting communications, they may also be applied to outreach efforts concerning other contentious wildlife management issues.

## Introduction

### Public attitudes regarding trophy hunting

In the United States, hunting remains a primary wildlife conservation and management strategy even as the number of active hunters has declined [1, 2]. With this demographic shift, wildlife managers, conservation organizations, and hunting associations increasingly work to mitigate conflict and maintain support for hunting among stakeholders not directly familiar with the practice, a task often accomplished through public outreach [1, 3, 4]. Despite the diminishing hunter population, surveys of U.S. residents have consistently indicated majority approval (>70%) for the practice since the 1990s [2, 3, 5]. However, this support varies according to hunters’ motivations: while hunting for subsistence or protection is broadly accepted, trophy hunting receives minimal support in the United States [3, 5, 6].

Trophy hunting describes the practice in which individual animals with particularly desirable physical characteristics or features (e.g., body size, horn or antler measurements) are hunted for preservation and display. Otherwise known as “sport” or “safari” hunting, it is usually practiced by hunters paying for the right to hunt, the services of a guide, and requisite equipment or staff [7]. Males of large, charismatic species are the most typical targets, from polar bears in northern Canada [8] to ungulates and large cats in sub-Saharan Africa [7].

Responsibly managed trophy hunting can serve as a tool to benefit local communities and surrounding ecosystems by improving socioeconomic and conservation outcomes [7–13]. Hunting license sales and land use fees can fund wildlife conservation initiatives, including population monitoring, habitat enhancement, and anti-poaching efforts [9]. Further, as trophy hunters are often willing to hunt in marginal or remote areas, their activities can underwrite wildlife conservation in locations that do not receive other types of tourist spending [10]. Trophy hunting can also reframe species that are otherwise considered nuisances or hazards as potentially valuable, thereby incentivizing the conservation of these animals and their associated ecosystems [7, 11]. Meanwhile, the socioeconomic benefits of trophy hunting include job opportunities, marketable skill training for local residents, and funding for community programs (e.g., infrastructure, education, healthcare) [8, 11, 12]. Additionally, game meat sold or donated to communities by trophy hunting operators can be a culturally significant source of protein [13].

Despite the global ubiquity of trophy hunting and its potential benefits when appropriately implemented, American attitudes toward the practice have been broadly negative for decades. In the 1980s, over three-quarters of surveyed U.S. residents disapproved of hunting for a trophy [14]. Recent studies indicate that American support for trophy hunting remains minimal; typically, only about a third of the public approves of the practice [3, 5]. Concerns about the morality or legitimacy of trophy hunting may contribute to this lack of acceptance [6]. In addition, hunting communications professionals have noted that efforts to discuss trophy hunting with members of the public are often stymied by misinterpretations of terminology and misunderstandings regarding the practice [1]. Negative misconceptions of trophy hunting are ubiquitous, including assertions that the practice is a threat to endangered species, a wasteful use of wildlife, or a form of animal cruelty [1, 4, 15, 16].

The pervasive antipathy for trophy hunting is evident in the popularity and influence enjoyed by anti-trophy hunting campaigns; these efforts shape public discourse even without enacting substantial policy changes [17, 18]. Citing the potential drawbacks of trophy hunting and highlighting instances of poor sportsmanship or violations of hunting regulations, activists call for national or global prohibitions on the practice [19]. Meanwhile, conservation practitioners’ ability to effectively respond to such efforts is hindered by the real and perceived difficulties associated with public outreach about this contentious form of hunting. In particular, a prevailing recommendation to separate attempts to change public attitudes toward trophy hunting through dedicated communications strategies from broader discussions of hunting in general may only serve to reinforce the stigma against the practice, thereby exacerbating the issues surrounding its place in the popular discourse [1]. Given the prevalence of anti-trophy hunting sentiments in the United States, hunters, wildlife managers, and other interested parties seek to improve their outreach efforts to change attitudes regarding the practice and maintain well-managed trophy hunting as a tool for socioeconomic development and wildlife conservation and management.

### Improving trophy hunting communications

Well-designed messaging programs could build support for trophy hunting in the face of widespread animosity toward the practice. One measure of success for such efforts is their capacity to address existing misbeliefs, a major challenge when communicating with the public about trophy hunting [1]. Information provided via messaging can dispel misconceptions and change attitudes about controversial or emotional subjects if audiences are willing to listen [20, 21]. Conventional anti-misinformation strategies include inoculation (warnings about misinformation alongside preemptive counterarguments), debunking of erroneous information, presentation of plausible alternative viewpoints, and encouraging careful evaluation of message and source credibility [21]. These tactics may be applied to trophy hunting communications to improve public attitudes by refuting the numerous misconceptions regarding the practice.

The success of a messaging campaign also depends on its ability to relate information to different audiences. To effectively persuade audiences, message designers must account for how the various identities of potential recipients may affect their response to a message’s contents and presentation [22, 23]. Social identity theory states that people formulate self-images according to group memberships. According to this theory, individual group members’ attitudes and behaviors are determined based on perceived ideals (norms) for the larger group [24]. As an example, in the United States, political affiliation strongly influences reasoning processes for many issues (e.g., gun control, vaccination); such ideologically motivated cognitions demonstrate adherence to party priorities and signal group affinities [25]. For environmental concerns, social identity can be a major determiner of individuals’ positions on given issues (e.g., the “conservative white male effect” seen in some climate change research, [26, 27]), thereby necessitating that related communications account for identity-driven cognitions to maximize efficacy [25, 28, 29]. In debates over wildlife management and hunting, polarization and high tension make appeals to group membership especially powerful [23, 30, 31]. Often, trust in state wildlife management agencies is linked to political affiliation; conservatives generally report lower trust in government programs even while holding traditionalist, utilitarian views of wildlife use [32]. Messaging can also resonate with other aspects of identity besides group membership. For instance, identity theory posits that personal values and morals exert substantial influence on self-concepts, ultimately driving behaviors and beliefs [33, 34]. Examples of such values include family, tradition, and spirituality [35]. Message designers can use these and other elements of identity to tailor communications to audiences to increase their efficacy.

The source of a message is just as important as its contents and relatability to recipients. Audiences must accept the credibility of a message’s author or distributor; otherwise, they may perceive information provided in the message as biased or incorrect, redoubling adherence to prior beliefs [21, 36]. Given the increasing trend of political polarization in the United States [37], perceptions of credibility are frequently linked to political affiliation. Audiences place greater trust in information from figures whose political beliefs align with their own [25, 36]. Notably, scientific information about contentious topics is subject to varying acceptance depending on the political alignment of both source and recipient, though this can be mitigated using data from apolitical or uncontested sources [22]. Prior research has suggested that wildlife and conservation agency personnel are generally viewed as credible providers of information about hunting, though it remains unclear whether this holds true for information about trophy hunting [4].

Existing trophy hunting communications, which are currently neither well-designed nor widely distributed, do not represent successful outreach efforts. Some outreach professionals even discourage public discussion of the practice. One popular guide to hunting communications labels trophy hunting a “perfect villain” used by anti-hunting activists to negatively influence broader attitudes toward hunting, encouraging its readers to steer debates away from trophy hunting to more palatable motivations (e.g., subsistence hunting) [1]. This argument positions trophy hunting as too objectionable to even be mentioned in public discussions, limiting conversations about the practice to intra-community discourse. To this point, research suggests that communications from hunting operations primarily target their customer base without attempting to appeal to a broader audience. A review of online marketing by safari hunting outfits found that such websites emphasize the value of the hunt to the hunter rather than issues of morality or sustainability; descriptions of pricing plans and species of interest are prioritized over promotion of ethical standards or conservation practices [38]. Trophy hunters, meanwhile, often discuss hunting in terms of achievement or accomplishment, which can be off-putting to the general public [4, 39].

By ignoring non-hunters’ concerns about trophy hunting in outreach materials, hunt operators and trophy hunters limit their ability to connect with audiences not already familiar with the practice. In doing so, they risk losing what little public support exists for trophy hunting as the social license (i.e., unofficial sanction) for the practice diminishes [3, 5, 40]. Thus, the problem of inadequate public-facing communication about trophy hunting persists. To address the issues with current outreach efforts and improve public attitudes concerning this controversial practice, new communication strategies are required. In particular, priming and message framing represent promising options, especially when used to tailor messages to specific target audiences.

Priming is a psychological process in which people’s responses to a given situation or piece of information are influenced by prior exposure to other information [41, 42]. In other words, priming describes how recent experiences subconsciously affect present reactions. In the context of trophy hunting and wildlife management, audiences could be primed to connect messages they receive with their place identity, relationships to nature, or social identity groups (such as political affiliation, hunter status, or conservation organization membership) in order to increase message acceptance [29, 31, 32, 43].

As with identity-focused priming, the frame of a message can substantially affect how audiences receive it. A message’s “frame” is the combined effect of its contents, context, organization, structure, and medium, all contributing to the overall presentation of the information contained in the message [44, 45]. Framing describes how people (re)conceptualize an issue based on contextualized information [46]. The same information can provoke different responses according to how it is framed for audiences. For example, messaging about invasive species mitigation invoking legal consequences of negligence is more likely to promote proper watercraft sterilization behaviors than messages emphasizing cultural norms surrounding sterilization [45]. Regarding the efficacy of different frames in improving hunting communications, research focused on developing pro-hunting messages has found receptive audiences for communications with different themes, including the economic and ecological benefits of such activities [4].

### Research goals

To address the growing need for effective communication about trophy hunting, this research intended to explore the effects of different message designs on public attitudes toward the practice. Therefore, we had two main objectives: to investigate how different combinations of identity priming items and message frames affect respondents’ attitudes toward trophy hunting and to compare respondents’ willingness to trust information about trophy hunting from different hypothetical sources. We hypothesized that messages emphasizing potential benefits of trophy hunting would increase respondents’ support for the practice as compared with a neutral control message (H1), and we anticipated that wildlife conservation-focused messages would have a greater positive effect than messages centered around the socioeconomic benefits of trophy hunting given the prevalence of the popular belief that trophy hunting poses a substantial threat to wildlife species [1, 15, 16] (H2). We also expected that priming for personal value identity or social identity would affect respondents’ post-message support for trophy hunting (H3). Finally, we predicted that respondents would be more trusting of trophy hunting communications from government agencies or conservation organizations than those from hunting associations (H4). Overall, this study aimed to evaluate how different aspects of message design can influence public attitudes regarding trophy hunting; we successfully achieved this goal and provided suggestions for improving future public-facing trophy hunting communications based on our findings.

## Materials and methods

### Survey and message design

We followed the recommendations of Dillman et al. [47] while developing our online survey instrument. After indicating their written consent to participate and completing an introductory section regarding attitudes towards hunting in general, respondents transitioned to the trophy hunting-specific message testing experiment, which employed a pretest-posttest design [48]. First, each respondent answered the same pretest question, indicating their current level of approval for “legal, regulated” trophy hunting using a five-point Likert scale (1 = “strongly disapprove” to 5 = “strongly approve”) with an additional “I don’t know” option. This question was based on prior research by Responsive Management and the National Shooting Sports Foundation [5]. Next, respondents were randomly assigned to one of nine treatment groups, where each treatment had two components: an identity-focused priming item (or lack thereof, as a control) and one of three sets of message content (including a control message) (Table 1).

**Table 1:** Description of message treatments.

		Message content		
		*Definition (control)*	*Wildlife conservation benefits*	*Socioeconomic benefits*
**Priming item**	***No prime (control)***	Treatment 1	Treatment 4	Treatment 7
	***Social identity prime***	Treatment 2	Treatment 5	Treatment 8
	***Value identity prime***	Treatment 3	Treatment 6	Treatment 9

Given our interest in the potential roles of social identity theory [24] and identity theory [33, 34] in determining approval for trophy hunting, we included questions that primed for both types of identities (independently) in this experiment. To maximize the effects of these primes, respondents answered their designated priming item just prior to viewing their assigned messaging [41, 42]. Respondents assigned to treatments subject to the value identity prime (Treatments 3, 6, and 9; Table 1), corresponding to identity theory [33, 34], were asked to consider a list of personal values adapted from the Chronic Pain Values Inventory [35], which consisted of family/friendship, conservation/stewardship, economic/financial success, growth and learning, and recreation/fun. Respondents were then instructed to rank these items according to the level of importance they would assign to each in their own lives and the lives of others. Respondents assigned to Treatments 1, 2, 4, 5, 7, and 8 (Table 1) received this question as part of the demographics section at the end of the survey. Respondents assigned to the “social identity” treatments (i.e., Treatments 2, 5, and 8; Table 1), corresponding to social identity theory [24], were asked to report their political alignment; options included “strongly conservative” (1), “moderately conservative” (2), “centrist” (3), “moderately liberal” (4), and “strongly liberal” (5). Respondents assigned to Treatments 1, 3, 4, 6, 7, and 9 received this question as part of the demographics section at the end of the survey.

After the identity priming item (or absence of one), respondents viewed one of three messages corresponding to their assigned frame (Table in S1 Appendix). The control message featured a single paragraph providing descriptive information about trophy hunting, including a basic definition of the term and a brief summary of typical trophy hunting operations [7, 49]. The two experimental messages contained the same descriptive information as the control message, and additional information related to the treatment frames was appended to the definitional paragraph. Prior research suggested that these messages should focus on the ecological and socioeconomic benefits of managed trophy hunting, as audiences have proven receptive to these two frames in similar contexts [4, 44]. Treatments 4–6 focused on the wildlife conservation benefits associated with managed trophy hunting [9, 50]. Treatments 7–9 emphasized the socioeconomic benefits that trophy hunting can provide to local communities and society at large [12, 49, 51].

To determine whether the messages resonated as intended, we asked respondents to indicate their agreement with a series of seven induction statements regarding their assigned message using a five-point Likert scale with an additional “I don’t know” option [52]. We then asked respondents the post-test question, which rephrased the pre-test question about approval for “legal, regulated” trophy hunting to reflect our interest in respondents’ attitudes following their exposure to the messages. Additionally, we employed a post-only design [48] where respondents were asked to indicate the likelihood of their trust in the message that they just read if it came from various hypothetical sources using a three-point scale (1 = “less likely,” 2 = “neither more nor less likely,” and 3 = “more likely”) with an additional “I don’t know” option. Respondents also answered questions about various demographic characteristics (i.e., age, gender, race/ethnicity, region, setting, household income, and highest level of education).

We cognitively pretested the survey from December 2022 to January 2023 and made revisions based on participants’ comments. The final survey instrument (text in S2 Appendix) and protocol were approved by the University of Arkansas at Monticello Institutional Review Board (#FANR1002).

### Survey distribution

The Qualtrics survey platform hosted the survey, and respondents were randomly selected from an opt-in panel maintained by Qualtrics. Respondents were required to reside in the United States and be over the age of eighteen to be eligible for the survey. Qualtrics used respondents’ self-reported demographic characteristics (namely, gender, age, race/ethnicity, region, and highest level of education) as quotas to ensure the sample was representative of the U.S. population as reported in the 2020 U.S. Census [53]. As a note, the racial and ethnic categories used in this study were adapted from the most recent U.S. Census [53]. We acknowledge the limitations of this methodology, including the possible disparities between our sample and the overall U.S. population, as well as the potential bias introduced by the online distribution of our survey, as differences in attitudes regarding trophy hunting may exist between American residents with and without reliable internet access [47]. Responses were collected between January and February of 2023. During this period, Qualtrics solicited responses from the online panel, adjusting the targeted distribution of the survey as the pre-established demographic quotas were met. Throughout this process, the survey company provided updates to the researchers and allowed for periodic inspection of data quality. Upon completion of data collection, we performed a final quality check on all responses, including removal of some responses due to suspected “straight-lining” [54].

### Data analysis

We used R statistical software version 4.2.2. to analyze our data [55]. For both the pre- and post-test questions regarding approval for trophy hunting and the induction checks, all “I don’t know” responses were converted to neutral responses prior to analysis to allow for paired analyses [56]. For the question about trust in various hypothetical message sources, all “I don’t know” responses were removed prior to analysis. Shapiro-Wilk tests and Levene’s tests (R package “car” [57]) indicated that our data violated assumptions of normality and homogeneity of variance. However, given the size of our sample and the general robustness of parametric tests such as ANOVA to violations of their prerequisite assumptions when applied to large-scale Likert type survey data, we elected to use parametric analyses to answer our research questions [58, 59]. For purposes of comparison, we included results from nonparametric alternatives for each parametric test we conducted in S3 Appendix.

We used paired t-tests to compare respondents’ attitudes regarding trophy hunting before and after viewing their assigned message [60]. We assessed the effect size of pre- and post-message differences via Cohen’s d, which we determined with the “effsize” package in R [61]. Effect size thresholds were as follows: |d| < 0.2 = "negligible,” 0.2 ≤ |d| < 0.5 = "small,” 0.5 ≤ |d| < 0.8 = "medium,” and |d| ≥ 0.8 = "large" [62]. We used one-way analysis of variance (ANOVA) tests to determine statistically significant differences in attitudes toward trophy hunting based on treatment group. We measured effect size using eta squared (η^2^), interpreted as follows: η^2^ < 0.01 = “negligible,” 0.01 ≤ η^2^ < 0.06 = “small,” 0.06 ≤ η^2^ < 0.14 = “medium”, and η^2^ ≥ 0.14 = “large” [63, 64]. One-way ANOVA was also used to determine significant differences in the induction checks based on treatment group, trust in the message based on hypothetical message source, and attitudes toward trophy hunting based on political alignment and personal values. When the ANOVA test results were significant, we performed multiple pairwise comparisons with a Bonferroni correction to assess which specific differences were statistically significant [60].

To determine the effects of different message treatments on respondents with opposing political affiliations or different personal values, we sorted the responses to relevant questions into binary response bins (e.g., liberal versus conservative, individuals who highly value conservation versus individuals who place a low value on conservation, etc.). For political affiliation, we collapsed the 5-point Likert scale into two options: conservative versus liberal, excluding centrists from the analysis [32]. Similarly, for personal values, a rank of 1 or 2 for a given value area indicated "highly valued,” and 4 or 5 was "low value,” while ranks of 3 (“medium value”) were excluded. For this analysis, we focused on respondents who highly valued “conservation/stewardship” and “economic/financial success,” as these values were directly related to the two experimental message frames. While the political identity groups were mutually exclusive, it was possible for a respondent to be in both high-rank personal value groups, as the two values are not opposite ends of the same spectrum. We used paired t-tests to compare pre- and post-message attitudes toward trophy hunting within each group. We also used one-way ANOVA tests and multiple pairwise comparisons with a Bonferroni correction to examine treatment effect on the pre- and post-message attitudes among respondents in each group [60]. Data and code used for all analyses are found in S4 and S5 Appendices.

## Results

### Sample demographics

Our final sample size was 2000 responses, and the demographics of the sample were consistent with those of the 2020 U.S. Census [53]. Slightly more than half of our respondents identified as women, and the mean age of our sample was 46.73 years old (Table 2). Nearly 60% of respondents identified as White (Non-Hispanic) alone; the rest belonged to one or more other racial or ethnic groups (Table 2). Over 35% reported that their highest level of formal education was at least a bachelor’s degree, and less than half had a total annual household income of $60,000 or more (Table 2). Over three-quarters of respondents resided in urban or suburban areas based on their own self-reporting (Table 2). Nearly 40% identified as either strongly or moderately conservative while almost a third identified as either strongly or moderately liberal (Table 3). Regarding personal values, 45% of respondents ranked “family/friendship” as the most important of the provided options. Just over one-fifth indicated that “economic/financial success” was the most important, and approximately 15% listed “conservation/stewardship” as being most important. “Growth and learning” ranked as the most important value for slightly over 12% of respondents, while 7% considered “recreation/fun” to be most important (Table 3).

**Table 2:** Summary of sample demographics.

Demographic characteristic	% of respondents	Demographic characteristic	% of respondents
*Age*	n = 1995	*Race/Ethnicity*	n = 2000
18–34 years old	30.42	American Indian or Alaska Native	3.95
35–54 years old	30.98	Asian	5.25
≥ 55 years old	38.60	Black or African American	11.10
***Total household income***	n = 2000	Hispanic/Latino	12.65
< $20,000	15.20	Native Hawaiian or Pacific Islander	0.15
$20,000 - $39,999	21.80	White (Non-Hispanic)	57.85
$40,000 - $59,999	18.85	Other	0.75
$60,000 - $79,999	14.70	Two or more races	8.30
$80,000 - $99,999	9.50	***Region***	n = 2000
$100,000 -$119,999	6.25	Northeast	17.25
$120,000 or more	9.50	Southeast	38.50
Do not wish to answer	4.20	Midwest	21.50
***Gender***	n = 1987	West	22.75
Man	46.80	***Highest level of education***	n = 2000
Woman	53.20	Less than 12th grade	3.20
***Setting***	n = 2000	High school graduate/GED	27.60
Urban	30.60	Some college/associate or technical degree	33.50
Suburban	45.85	Bachelor’s degree	24.10
Semi-rural	7.45	Graduate degree	11.60
Rural	16.10		

Race/Ethnicity categories adapted from: U.S. Census Bureau. QuickFacts: United States. 2020 [cited 4 July 2023]. In: United States Census Bureau QuickFacts [Internet]. Washington DC: U.S. Department of Commerce. [about 4 screens]. Available from: https://www.census.gov/quickfacts/fact/table/US/PST045221

**Table 3:** Descriptive statistics and ANOVA results showing differences in approval for trophy hunting across identity groups.

Characteristic	% of respondents	Mean approval for trophy hunting (SD)	Characteristic	% of respondents	Mean approval for trophy hunting (SD)
***Political alignment*** (*F* = 18.56***, η^2^ = 0.04)			***Economic/financial success rank*** (*F* = 3.74, η^2^ < 0.01)		
Strongly conservative	13.00	2.81^a^ (1.43)	1	20.35	2.48^a^ (1.29)
Moderately conservative	26.80	2.57^a^^b^ (1.24)	2	22.35	2.49^a^ (1.27)
Centrist	27.25	2.42^b^ (1.19)	3	21.80	2.31^a^(1.27)
Moderately liberal	21.95	2.19^c^ (1.23)	4	17.15	2.51^a^ (1.31)
Strongly liberal	11.00	1.98^c^ (1.25)	5	18.35	2.28^a^ (1.21)
***Family/friendship rank*** (*F* = 2.80, η^2^ < 0.01)			***Growth and learning rank*** (*F* = 0.11, η^2^ < 0.01)		
1	45.00	2.38^a^ (1.24)	1	12.20	2.50^a^ (1.30)
2	20.70	2.40^a^ (1.30)	2	24.05	2.29^a^ (1.25)
3	13.75	2.44^a^ (1.30)	3	27.50	2.49^a^ (1.27)
4	12.20	2.39^a^ (1.27)	4	21.10	2.36^a^ (1.28)
5	8.35	2.62^a^ (1.32)	5	15.15	2.46^a^ (1.25)
***Conservation/stewardship rank*** ***(****F* = 23.61***, η^2^ = 0.01)			***Recreation/fun rank*** (*F* = 28.70***, η^2^ = 0.01)		
1	15.45	2.19^a^ (1.26)	1	7.00	2.76^a^ (1.30)
2	17.75	2.35^a^ (1.25)	2	15.15	2.59^a^^b^ (1.28)
3	18.70	2.34^a^ (1.26)	3	18.25	2.47^a^^b^^c^ (1.27)
4	22.80	2.43^a^^b^ (1.24)	4	26.75	2.39^b^^c^ (1.27)
5	25.30	2.63^b^ (1.31)	5	32.85	2.25^c^ (1.25)

^a^Means sharing a letter are not significantly different at α = 0.05

^b^*** indicates significance at p < 0.001

^c^Scale is 1 = “Strongly disapprove” to 5 = “Strongly approve”

### Induction check

Overall, respondents tended to disagree that their assigned message aligned with their personal views, and they generally indicated that they wanted to “argue back” with what they read (Table 4). Respondents were relatively neutral regarding both the statement that the message they read addressed their concerns about trophy hunting (indicating that the messages did not adequately respond to such concerns) and the statement that the message provided factual evidence about the benefits of trophy hunting (suggesting that the message contents were generally seen as at least somewhat factual, though not overwhelmingly so) (Table 4). Overall, respondents also tended to be fairly neutral when asked whether their assigned message made them believe that trophy hunting could be good for conservation or for local people, indicating that the messages did not provoke strong feelings about the positive impacts of trophy hunting (Table 4). Finally, on average, respondents neither agreed nor disagreed that the message they read was well-reasoned (Table 4).

**Table 4:** ANOVA results showing differences in response to induction checks by treatment.

	Treatment group									
	1. No prime control	2. Social identity control	3.Values control	4. No prime wildlife	5. Social identity wildlife	6. Values wildlife	7. No prime socioeconomic	8. Social identity socioeconomic	9. Values socioeconomic	All respondents
**Induction check statement**	**Mean agreement (SD)**									
*The message that I just read*.* *.* *.										
is aligned with my personal views.	2.51a (1.29)	2.73ab (1.40)	2.50a (1.32)	3.06bcd (1.21)	3.19ce (1.13)	3.25cfg (1.17)	2.96bef (1.23)	2.83ade (1.17)	2.87adeg (1.25)	2.88 (1.27)
makes me feel like trophy hunting can be good for conservation	2.54a (1.27)	2.60ab (1.35)	2.63ab (1.31)	3.24c (1.22)	3.23c (1.13)	3.20c (1.22)	3.10c (1.21)	2.96bc (1.27)	2.87abc (1.29)	2.93 (1.28)
makes me feel like I wanted to "argue back" to what was stated in the message	3.31ab (1.25)	3.46a (1.15)	3.26ab (1.23)	3.05b (1.15)	3.03b (1.17)	3.16ab (1.13)	3.26ab (1.18)	3.33ab (1.19)	3.24ab (1.16)	3.23 (1.18)
addressed my concerns about trophy hunting	2.90ab (1.33)	2.95ab (1.30)	2.86a (1.26)	3.18abc (1.16)	3.26bc (1.16)	3.37c (1.18)	3.20abc (1.26)	3.09abc (1.19)	3.12abc (1.20)	3.10 (1.24)
provided factual evidence about the benefits of trophy hunting	2.87ab (1.28)	2.96abc (1.29)	2.85ad (1.21)	3.32c (1.06)	3.29c (1.09)	3.29c (1.16)	3.29c (1.18)	3.23bc (1.19)	3.05bcd (1.18)	3.13 (1.20)
makes me feel like trophy hunting can be good for local people	2.54a (1.24)	2.72ab (1.38)	2.66a (1.28)	3.23c (1.21)	3.21c (1.22)	3.23c (1.22)	3.27c (1.26)	3.18c (1.28)	3.06bc (1.26)	3.01 (1.29)
is well-reasoned	2.66a (1.25)	2.80ab (1.33)	2.69ac (1.31)	3.19d (1.18)	3.26d (1.15)	3.22d (1.15)	3.19d (1.22)	3.11bd (1.20)	3.06bcd (1.26)	3.02 (1.25)

^a^Means sharing a letter are not significant different at α = 0.05

^b^Scale is 1 = “Strongly disagree” to 5 = “Strongly agree”

One-way ANOVA tests and post-hoc multiple pairwise comparisons with a Bonferroni correction, indicated that the various treatments produced significant differences in the messages’ resonance with respondents. The responses of those who saw the conservation message (Treatments 4–6) generally differed from those of their counterparts who viewed the control message (Treatments 1–3) (Table 4). For example, while respondents who saw the conservation message (Treatments 4–6) tended to perceive the message as more in line with their personal views than those who saw the control message (Treatments 1–3) (*F*(8, 1990) = 10.56, *p* < 0.001, η^2^ = 0.04), mean agreement between Treatment 4 and Treatment 2 did not significantly differ (Table 4). Mean agreement that the messages provided factual evidence of the benefits of trophy hunting also generally adhered to this pattern (*F*(8, 1989) = 5.98, *p* < 0.001, η^2^ = 0.02), as did responses to statements about the messages being well-reasoned (*F*(8, 1987) = 8.40, *p* < 0.001, η^2^ = 0.03) and able to convince respondents that trophy hunting could be good for conservation (*F*(8, 1990) = 11.52, *p* < 0.001, η^2^ = 0.04) (Table 4). Respondents who viewed either the conservation or socioeconomic benefits messages (Treatments 4–9) tended to be slightly more willing to agree that the message convinced them about potential socioeconomic benefits of trophy hunting than those who saw the control message (Treatments 1–3) (*F*(8, 1988) = 11.66, *p* < 0.001, η^2^ = 0.04) (Table 4). Other induction checks did not follow the common pattern: the only significant differences regarding respondents’ desire to “argue back” with the messages were that those in Treatment 2 were, on average, significantly more likely to agree with this sentiment than those who received Treatment 4 or Treatment 5 (*F*(8, 1990) = 2.99, *p* = 0.003, η^2^ = 0.01) (Table 4). Finally, while the nine treatments produced some significant differences in mean agreement with the statement about addressing concerns (*F*(8, 1990) = 4.37, *p* < 0.001, η^2^ = 0.02), there were no clear patterns based on either priming item or message version (Table 4).

### Message treatment effect on trophy hunting attitudes

Among all respondents, mean approval for trophy hunting prior to viewing any message was low ($\bar{x}$ (SD) = 2.41 (1.27)). The total number of respondents randomly assigned to each treatment group ranged from 203 to 231, and a one-way ANOVA test found no significant differences in average pre-treatment support for trophy hunting based on treatment group (Table 5). Supporting H1, all treatments including either the conservation or the socioeconomic benefits message significantly increased mean approval for trophy hunting, though the effect size varied (Table 5). A one-way ANOVA and post-hoc multiple pairwise comparisons with a Bonferroni correction revealed some significant differences in post-treatment mean approval for trophy hunting between treatments (Table 5). However, inconsistent with H2 and H3, we found no significant differences in post-message approval for trophy hunting between respondents who viewed the conservation benefits message and those who viewed the socioeconomic benefits message, nor did we observe any patterns in post-message attitudes toward the practice based on the priming items (Table 5).

**Table 5:** ANOVA and paired t-test results showing treatment effect on pre- and post-message approval for trophy hunting.

Treatment	n	Pre-test mean (SD)	Post-test mean (SD)	t Statistic	Cohen’s d
***All respondents***		*F* = 1.38, η^2^ < 0.01	*F* = 6.28***, η^2^ = 0.02		
1. No prime control	226	2.38a (1.30)	2.42a (1.31)	-0.50	-0.03
2. Social identity control	225	2.56a (1.35)	2.59ab (1.39)	-0.44	-0.03
3. Values control	227	2.39a (1.21)	2.52ac (1.31)	-2.20*	-0.15
4. No prime wildlife	227	2.44a (1.23)	3.00d (1.24)	-7.75***	-0.51
5. Social identity wildlife	231	2.52a (1.30)	2.94bd (1.32)	-5.09***	-0.34
6. Values wildlife	227	2.44a (1.28)	3.00d (1.25)	-6.86***	-0.46
7. No prime socioeconomic	203	2.21a (1.25)	2.89bcd (1.28)	-8.13***	-0.57
8. Social identity socioeconomic	224	2.40a (1.22)	2.81bcd (1.26)	-5.78***	-0.39
9. Values socioeconomic	210	2.34a (1.29)	2.71abcd (1.26)	-4.70***	-0.32
***Conservative respondents only***		*F* = 0.52, η^2^ < 0.01	*F* = 2.26*, η^2^ = 0.02		
1. No prime control	82	2.66a (1.32)	2.70a (1.31)	-0.27	-0.03
2. Social identity control	97	2.72a (1.40)	2.75a (1.49)	-0.33	-0.03
3. Values control	88	2.64a (1.30)	2.91a (1.34)	-2.68**	-0.29
4. No prime wildlife	91	2.63a (1.23)	3.30a (1.12)	-5.89***	-0.62
5. Social identity wildlife	89	2.75a (1.35)	3.17a (1.36)	-3.45***	-0.37
6. Values wildlife	91	2.74a (1.29)	3.23a (1.21)	-3.55***	-0.37
7. No prime socioeconomic	89	2.42a (1.30)	2.99a (1.27)	-4.88***	-0.52
8. Social identity socioeconomic	89	2.64a (1.29)	2.96a (1.19)	-2.77**	-0.29
9. Values socioeconomic	80	2.63a (1.31)	2.99a (1.24)	-2.63*	-0.29
***Liberal respondents only***		*F* = 1.28, η^2^ = 0.02	*F* = 2.66**, η^2^ = 0.03		
1. No prime control	92	2.18a (1.28)	2.20ab (1.38)	-0.11	-0.01
2. Social identity control	73	2.49a (1.40)	2.58ab (1.44)	-0.76	-0.09
3. Values control	78	2.01a (1.13)	2.06a (1.19)	-0.60	-0.07
4. No prime wildlife	65	2.08a (1.20)	2.52ab (1.32)	-3.65***	-0.45
5. Social identity wildlife	69	2.19a (1.23)	2.67ab (1.29)	-3.22**	-0.39
6. Values wildlife	72	2.07a (1.26)	2.74ab (1.28)	-5.02***	-0.59
7. No prime socioeconomic	60	1.90a (1.16)	2.83b (1.38)	-5.16***	-0.67
8. Social identity socioeconomic	74	2.11a (1.18)	2.57ab (1.25)	-3.55***	-0.41
9. Values socioeconomic	76	2.03a (1.26)	2.47ab (1.27)	-3.30**	-0.38
***High conservation only***		*F* = 0.26, η^2^ < 0.01	*F* = 4.90***, η^2^ = 0.06		
1. No prime control	78	2.26a (1.29)	2.13a (1.23)	1.11	0.13
2. Social identity control	69	2.39a (1.22)	2.46ab (1.37)	-0.65	-0.08
3. Values control	84	2.27a (1.25)	2.29ac (1.28)	-0.14	-0.02
4. No prime wildlife	65	2.20a (1.15)	3.14b (1.16)	-6.19***	-0.77
5. Social identity wildlife	74	2.35a (1.34)	2.93bc (1.24)	-4.15***	-0.48
6. Values wildlife	73	2.36a (1.32)	2.86bc (1.26)	-3.22**	-0.38
7. No prime socioeconomic	67	2.16a (1.27)	2.87bc (1.38)	-4.03***	-0.49
8. Social identity socioeconomic	80	2.23a (1.17)	2.53ab (1.23)	-2.65**	-0.30
9. Values socioeconomic	74	2.27a (1.29)	2.58ab (1.32)	-2.26*	-0.26
***High economic only***		*F* = 1.33, η^2^ = 0.01	*F* = 2.59**, η^2^ = 0.02		
1. No prime control	98	2.64a (1.27)	2.70ab (1.36)	-0.60	-0.06
2. Social identity control	109	2.59a (1.40)	2.63ab (1.42)	-0.48	-0.05
3. Values control	92	2.30a (1.15)	2.48a (1.32)	-1.91	-0.20
4. No prime wildlife	95	2.55a (1.23)	3.00ab (1.24)	-4.20***	-0.43
5. Social identity wildlife	85	2.41a (1.32)	2.86ab (1.32)	-3.01**	-0.33
6. Values wildlife	92	2.58a (1.24)	3.17b (1.23)	-5.08***	-0.53
7. No prime socioeconomic	93	2.18a (1.26)	2.96ab (1.28)	-6.00***	-0.62
8. Social identity socioeconomic	104	2.55a (1.26)	2.92ab (1.27)	-3.84***	-0.38
9. Values socioeconomic	86	2.53a (1.34)	3.00ab (1.27)	-3.70***	-0.40

^a^Means sharing a letter are not significantly different at α = 0.05

^b^* indicates significance at *p* < 0.05, ** indicates significance at *p* < 0.01, *** indicates significance at *p* < 0.001

^c^Scale is 1 = “Strongly disapprove” to 5 = “Strongly approve”

^d^“High conservation” refers to respondents who ranked “Conservation/Stewardship” as one of their top two personal values; “High economic” refers to respondents who ranked “Economic/Financial success” as one of their top two personal values

Based on the results of a one-way ANOVA test and a post-hoc multiple pairwise comparison with a Bonferroni correction, we found that conservative respondents were more supportive of trophy hunting than their liberal counterparts, but those identifying as moderately conservative did not significantly differ from centrists (Table 3). After dividing the sample into liberals and conservatives, we found that both groups generally disapproved of trophy hunting before viewing any message (Table 5). The number of conservative respondents randomly assigned to each treatment group ranged from 80 to 97 while treatment group size for liberal respondents ranged from 60 to 92 (Table 5). One-way ANOVA tests indicated that pre-test mean approval for trophy hunting did not significantly differ by treatment for conservatives or liberals (Table 5). For conservative respondents, all treatments aside from Treatment 1 and Treatment 2 significantly improved attitudes towards trophy hunting (Table 5). Similarly, paired t-tests found that all treatments including either the conservation or socioeconomic benefits message (Treatments 4–9) significantly increased mean approval for trophy hunting among liberal respondents while those including the control message (Treatments 1–3) produced no change (Table 5). Although a one-way ANOVA test indicated that conservatives’ post-test approval differed significantly by treatment, post-hoc multiple pairwise comparisons with a Bonferroni correction did not find any such differences; meanwhile, the only significant difference in liberals’ post-test attitudes was that those in Treatment 7 tended to be more approving than those in Treatment 3 (Table 5).

One-way ANOVA tests and post-hoc multiple pairwise comparisons with a Bonferroni correction indicated that the only significant differences in attitudes regarding trophy hunting based on the relative importance of respondents’ personal values were that those who deemed “conservation/stewardship” the least important value from among the provided options tended to be more supportive of trophy hunting than those who reported it as being of greater importance, and those who ranked “recreation/fun” as being of the highest importance were more approving of the practice than those who considered it to be either their lowest or second-lowest priority (Table 3). Respondents who highly valued “conservation/stewardship” (approximately 33% of the sample) were largely unsupportive of trophy hunting before viewing any messages ($\bar{x}$ (SD) = 2.28 (1.25)), and pre-test approval for the practice among those who highly valued “economic/financial success” (just under 43% of respondents) was likewise low ($\bar{x}$ (SD) = 2.49 (1.28)) (Table 5). The number of respondents who highly valued “conservation/stewardship” who were randomly assigned to each treatment group ranged from 65 to 84 while treatment group size for respondents who highly valued “economic/financial success” ranged from 85 to 109 (Table 5). One-way ANOVA tests found that pre-test mean approval for trophy hunting did not significantly differ by treatment for either group (Table 5). For both groups, paired t-tests showed that treatments including either the conservation or socioeconomic benefits message significantly increased mean approval for trophy hunting while those including the control message had no significant effect on attitudes toward the practice (Table 5). Among those who highly valued “conservation/stewardship,” Treatment 4 had the greatest effect, while for respondents who highly valued “economic/financial success”, Treatment 6 and Treatment 7 provoked the most substantial changes (Table 5). A one-way ANOVA and post-hoc multiple pairwise comparison with a Bonferroni correction found that among respondents who highly valued “conservation/stewardship”, Treatment 1 resulted in a significantly lower post-test mean approval level for trophy hunting than treatments including the conservation message (Treatments 4–6) and Treatment 7, and Treatment 3 had a significantly lower post-test mean approval than Treatment 4 (Table 5). Meanwhile, the only significant difference in post-test attitudes toward trophy hunting among respondents who highly valued “economic/financial success” was that those in Treatment 6 tended to be more approving than those in Treatment 3 (Table 5).

### Trust in message sources

Consistent with H4, a one-way ANOVA and post-hoc multiple pairwise comparison with a Bonferroni correction indicated that respondents found Safari Club International (SCI) to be the least trustworthy provider of information among the available sources (Table 6). This was the only entity for which respondents reported a decreased likelihood of trust in hypothetical messaging (i.e., mean likelihood < 2.0) (Table 6). Notably, respondents tended to view information from the South African Department of Environmental Affairs (SADEA) as neither more nor less likely to be trusted, yet average trust placed in messages from the U.S. Fish & Wildlife Service (USFWS) was significantly higher (Table 6). The highest level of trust was reserved for USFWS or conservation-focused organizations such as the World Wildlife Fund (WWF) or The Nature Conservancy (TNC) (Table 6).

**Table 6:** Descriptive statistics and ANOVA results showing differences in mean likelihood of trust in message sources.

Message source (*F* = 126.30***, η^2^ = 0.06)	Less likely	Neither more nor less likely	More likely	I don’t know	Mean trust (SD)
A scientific journal	10.86%	36.74%	41.99%	10.41%	2.35a (0.69)
South African Department of Environmental Affairs (SADEA)	18.47%	40.34%	30.13%	11.06%	2.13b (0.73)
Safari Club International (SCI)	24.90%	39.80%	23.30%	12.00%	1.98c (0.74)
The Nature Conservancy (TNC)	9.00%	32.47%	48.72%	9.80%	2.44d (0.67)
U.S. Fish and Wildlife Service (USFWS)	10.60%	34.50%	47.15%	7.75%	2.40ad (0.69)
World Wildlife Fund (WWF)	10.72%	31.45%	48.77%	9.06%	2.42d (0.69)

^a^Means sharing a letter are not significantly different at α = 0.05

^b^Scale is 1 = “Less likely” to 3 = “More likely”

## Discussion

Following previous studies, our respondents were generally unsupportive of trophy hunting before viewing any messaging [3, 5, 6, 65]. Further, consistent with earlier research and our initial hypothesis (H1), messages framed around environmental or socioeconomic benefits of managed trophy hunting significantly improved respondents’ attitudes toward the practice [4]. However, in contrast to H2, both types of benefits-focused message frames appeared equally effective in positively influencing respondents’ support for trophy hunting. Even so, this improvement was not substantial enough to counter established negative attitudes toward the practice: even after viewing their assigned message, all respondents remained largely unsupportive of trophy hunting. Benefits-focused messages tended to reduce the intensity of their disapproval for the practice rather than prompting a shift to outright approval. Communications professionals recognize the difficulty of changing ingrained negative attitudes; it is far easier to reinforce positive attitudes or improve neutral views [66]. Mitigating public hostility to trophy hunting may therefore be more feasible than building widespread support for the practice.

There are several potential explanations for the limited effect of the messages on respondents’ attitudes regarding trophy hunting. Communications research emphasizes the importance of repetition in changing attitudes toward controversial or emotionally distressing topics, particularly when misconceptions abound [20, 21]. Considering the negative beliefs and strong feelings surrounding discussions of trophy hunting, it is unsurprising that our respondents remained unsupportive of trophy hunting after viewing a single, brief message [1, 4, 15, 16]. Multiple, mutually-reinforcing communications may produce more appreciable changes in public attitudes regarding the practice. To explore this possibility, future research might use a repeated measures design to examine how attitudes toward trophy hunting may change following exposure to several corroborative messages over a longer duration than that which the present study was able to evaluate using its simple pre-post design. Successful challenges to established misbeliefs should also include credible rebuttals to anticipated arguments against the messages’ contents within the messages themselves [21]. Failure to incorporate such counterarguments into a messaging campaign can limit its efficacy. For example, a study exploring attitudes toward a possible wolf reintroduction found that messages promoting wolf reintroduction that ignored opposing arguments were perceived as more extreme, and therefore less persuasive, than those acknowledging the opposition’s concerns [44]. In our study, the messages framed around the benefits of managed trophy hunting challenged beliefs that the practice invariably threatens wildlife conservation or the social order [16], but neither message explicitly addressed potential counterclaims. After reading their assigned message, many respondents indicated that they wished to “argue back.” Direct engagement with possible counterarguments may have reduced this sentiment. Future research could evaluate the efficacy of repetition, preemptive refutation of counterclaims, and similar strategies in improving informative trophy hunting communications [21].

Although our benefits-focused messages could be improved via different communication techniques, it is also possible that the frames we selected were not optimally suited for changing attitudes about trophy hunting. While concerns about environmental and economic impacts of trophy hunting do feature in discussions regarding the practice, ethical issues often dominate such discourse [1, 4, 6, 15, 16, 18]. Emotionally-charged depictions of trophy hunting as cruel, wasteful, or an illegitimate use of wildlife powerfully influence popular sentiments, overshadowing data-driven communications [1, 16, 18]. Our messages were not made to appeal to emotion, instead consisting of a basic definition of trophy hunting and, for the two experimental messages, descriptions of associated positive outcomes for local ecosystems [9, 50] or communities [12, 49, 51]. In a post-message induction check, respondents were somewhat ambivalent regarding these messages’ ability to address their concerns about trophy hunting. Given the frequency with which ethical issues arise in conversations about trophy hunting, some of the unaddressed concerns among our respondents are likely moralistic in nature [1, 6]. However, we were unable to directly evaluate this possibility due to our limited focus on the efficacy of exclusively facts-based messaging in influencing attitudes regarding trophy hunting. The inability to navigate emotional or ethical issues is well-documented among hunting outreach experts [1, 4]. For example, animal rights-based appeals are ubiquitous in wildlife use and trophy hunting debates, yet wildlife managers and hunting professionals often fail to manage them as skillfully as anti-hunting activists [1, 15, 67]. Messages that simultaneously confront ethical questions surrounding trophy hunting and educate recipients about the practice may bolster public support more than purely informative messages. Further research could expand upon our findings by exploring the effectiveness of messages that integrate emotional or ethical appeals alongside information about the benefits associated with well-managed trophy hunting. However, such communications must also provide balanced, scientific information to maintain their credibility [4].

While framing messages around the benefits of managed trophy hunting significantly improved respondents’ attitudes toward the practice, we did not observe similar patterns regarding the identity priming items, contrary to our initial expectations (H3). This absence of notable priming effects could stem from the overarching consensus within our sample concerning attitudes toward trophy hunting. In general, identity-based priming is most effective when there are clearly delineated behavioral or attitudinal norms associated with the relevant identities [23, 41]. In this case, we found consistently low approval for trophy hunting among the various identity groups we evaluated. Respondents across the political spectrum expressed disapproval for trophy hunting, differing only in the intensity of said disapproval. This suggests that attitudes toward trophy hunting are not closely tied to membership in social identity groups based on political alignment, and this absence of partisanship makes support for the practice a poor signal of affinity to one group over another [24, 25]. Similarly, among the personal value groups, any significant differences in attitudes toward trophy hunting tended to be in the degree to which they disapproved of the practice. These findings indicate that support for trophy hunting may not be substantively affected by the different aspects of values-driven personal identity evaluated in our survey. From this, we posit that U.S. residents may not consider their attitudes towards the practice to be an important component of their morally constructed self-concepts (i.e., regardless of the source or strength of their disdain for trophy hunting, they do not form an identity as a “person opposed to trophy hunting”) [33, 34].

Ultimately, unlike other ecological issues, trophy hunting was not subject to strong ideologically-motivated cognitions based on political alignment or personal values [25, 28]. This lack of group-based polarization meant that priming respondents to consider these identities did not create differences in their attitudes toward the practice. These results suggest that targeting trophy hunting communications to political affiliation or the personal value areas used in this experiment may be ineffective, as they do not substantially influence perceptions of the practice. Other research has indicated that identity-based appeals are most effective when the invoked identities are relevant to the subject at hand and genuinely important to recipients [43]. Given the limited influence of the identities explored in this study on respondents’ attitudes regarding trophy hunting, targeted messages may be more effective if they appeal to other facets of audiences’ identities. Future research might explore priming for these other identities, concentrating on those most directly related to trophy hunting (e.g., hunter status), as these would be the most likely to influence attitudes toward the practice [29].

A final takeaway from this research is that audience trust in trophy hunting messages depends on the source of those messages. In general, our respondents expressed limited trust in trophy hunting messaging from Safari Club International (SCI). Given SCI’s status as a hunting organization, respondents might have perceived information about trophy hunting from this source as biased, thereby reducing their trust and acceptance of said information [21]. In contrast, respondents reported a greater likelihood of trusting communications from the U.S. Fish and Wildlife Service (USFWS) and two prominent conservation organizations. Taken together, these findings support our earlier hypothesis regarding which institutions the public may be willing to trust for information about trophy hunting (H4). Interestingly, our respondents’ confidence in USFWS was high despite the American public’s growing distrust in the federal government [68]. It may be that USFWS is viewed as a credible authority when it comes to wildlife management, even if the government is generally not trusted. Research into climate change communications suggests that contentious scientific information from government agencies can be acceptable when presented in a nonpartisan manner [22]. However, other studies report widespread distrust in state wildlife management agencies, which would suggest that trust in USFWS should also be low [32]. Finally, messages from the South African Department of Environmental Affairs were not granted the same high trust as those from USFWS. This could be due to our American sample’s unfamiliarity with foreign government agencies, but it also fits established patterns of Westerners disregarding African nations’ right to manage their environments according to their own goals irrespective of the preferences of former colonial powers [18, 19, 69]. Regardless, this slight incongruity with H4 (which posited that respondents would generally perceive messages from government agencies as trustworthy) demonstrates that the American public does not view all governmental bodies as equally reliable sources of information about trophy hunting.

Altogether, our findings illustrate the prevalence and resilience of disapproval for trophy hunting among U.S. residents. Messages framed around the ecological and socioeconomic benefits of trophy hunting did improve respondents’ attitudes regarding the practice, but they were ultimately unable to provoke dramatic attitudinal shifts. Although the increase in support resulting from the benefits-focused messaging was small, it still represents an important development in the nascent field of trophy hunting communications. This finding demonstrates that it is possible to change negative attitudes toward trophy hunting, however slightly, through messaging. This casts doubt on the portrayal of trophy hunting as a “perfect villain” for anti-hunting activists that should be separated from broader discussions about hunting [1]: if disapproval for the practice can be mitigated by simple messaging, then trophy hunting is not such a “perfect villain.” Instead, public sentiments toward the practice can be shifted in a more positive direction given time and effort. However, it should be noted that due to our simple pre-post study design, we were not able to measure the persistence of the attitudinal change expressed by our respondents over the long term. Thus, it is possible that the observed reduction in respondents’ disapproval for trophy hunting after viewing messages that highlighted positive outcomes associated with the practice may represent a temporary effect. Additionally, the usage of an opt-in panel for this study may affect the generalizability of our findings, as differences may exist between the attitudes of respondents from online survey panels and those of the broader public [47, 70]. Further research can build on the foundations established by this study to ascertain whether exposure to positively framed trophy hunting communications results in lasting changes in public attitudes toward this contentious type of hunting.

Although we recognize that this study does not provide absolute recommendations for improving trophy hunting communication, we evaluated the potential utility of some message design options as possible starting points, providing suggestions for future research into this subject. Notably, while we observed some differences in how the nine message treatments were received by the identity groups included in this study, we did not discern any overall patterns regarding the relative influence of either the priming items or message frames between identity groups. Instead, messaging highlighting ecological or socioeconomic benefits associated with managed trophy hunting reduced disapproval for the practice across the sample. This finding illustrates the promise that benefits-focused messages might have for future trophy hunting outreach: to improve negative attitudes toward this controversial form of hunting, conservation practitioners should emphasize the potential positive outcomes that it can create for local communities and ecosystems in their public-facing communications. Further consideration of our results and other research suggests that pairing such informative communications with emotional appeals may be even more effective in changing attitudes regarding trophy hunting, though additional research is required to test this hypothesis. It is also important that wildlife professionals set realistic goals concerning the impacts of their trophy hunting outreach efforts. As previously noted, communications research has demonstrated the difficulty of challenging established negative attitudes through messaging [66]. The relatively minor attitudinal changes prompted by our messages suggest that this principle holds true for trophy hunting communications. Thus, an incremental shift toward more neutral attitudes toward trophy hunting may be a more achievable target for outreach efforts than striving to convert existing disapproval for the practice to outright approval. Finally, given our findings regarding the varying degree of trust respondents placed in messages about trophy hunting from different sources, we advise that wildlife professionals ensure that such messaging is delivered by entities that are likely to be perceived as trustworthy by audiences (e.g., conservation-focused organizations) in order to maximize its efficacy.

Considering the popularity of recent anti-trophy-hunting campaigns and the frequent calls for the U.S. government to enact legislation against the practice, the window of opportunity to practically apply trophy hunting communications research may soon close [11, 69]. Without intervention, public tolerance of trophy hunting will likely remain minimal or decline [40]. Wildlife managers, policymakers, and hunters should therefore act quickly to maintain trophy hunting as a viable conservation and management tool.

## Supporting information

S1 Appendix Full text of message contents. (DOCX)

S2 Appendix Example survey version (Treatment 3: Control message and values prime). (DOCX)

S3 Appendix Results of nonparametric testing. (DOCX)

S4 Appendix Exploring the efficacy of identity priming and message framing in influencing American attitudes toward trophy hunting data set. (XLSX)

S5 Appendix Exploring the efficacy of identity priming and message framing in influencing American attitudes toward trophy hunting R code for analyses. (PDF)
