# Supplementary material for: Exploring the efficacy of identity priming and message framing in influencing American attitudes toward trophy hunting
Source: PLoS One. 2024 Nov 7;19(11):e0312949. doi: 10.1371/journal.pone.0312949 (PMC11542780; doi:10.1371/journal.pone.0312949)
Supplement: S1 Appendix — (DOCX) [file pone.0312949.s001.docx]

**S1 Appendix. Full text of message contents**

| Definition (control) message |
| --- |
| "Well-managed trophy hunting can be defined as recreational hunting that involves the limited, regulated harvest of high-value wildlife species. The individual animals are usually chosen for their notable physical traits (e.g., large horns, tusks, or body size) for the purpose of displaying part or all of the hunted animal. Trophy hunters pay substantial fees for the ability to hunt, usually in the company of a professional hunting guide. The trophy hunting industry is run by hunting operators who market and sell hunts to clients (often at international hunting conventions), lease or own hunting areas (often private or community-owned lands), and employ the requisite staff (e.g., professional hunters, trackers, drivers, skinners, and camp staff)." |
| Wildlife conservation benefits message [shown in addition to control] |
| "The income from trophy hunting allows landowners to financially benefit from having wildlife and habitat on their land. In other words, trophy hunting provides an economic incentive for landowners to conserve wildlife and habitat.  Income from trophy hunting serves as a way landowners can afford to keep the habitat they have rather than developing their land or using it for pastureland or agriculture, reducing the amount of wildlife habitat lost to these activities. By maintaining their land for this purpose, landowners provide habitat for countless bird, insect, small mammal, reptile, and fish species in addition to the desired game species.  Additionally, revenue from trophy hunting is often reinvested into the land to fund wildlife conservation actions, including:  - habitat management (e.g., removing invasive plants from the land)  - wildlife population monitoring and wildlife research  - anti-poaching enforcement  Trophy hunting operations in Sub-Saharan Africa have provided incentives to conserve an area of wildlife habitat greater than 6x the size of the U.S. National Park System. In South Africa, specifically, nearly 50 million acres of wildlife habitat is conserved within private hunting lands (16.8% of the country's total land area). These private hunting lands have helped with endangered species recovery, including the rhinoceros, black wildebeest, and Cape Mountain zebra." |
| Socioeconomic benefits message [shown in addition to control] |
| "The income from trophy hunting can provide social and economic benefits to local people and the surrounding area.  Trophy hunting can help local people, communities, and the economy by promoting economic growth, job creation, and food security. By attracting international tourists, trophy hunting can insert new money into the local economy. Hunting operations can also create jobs within rural communities, predominantly in the agricultural sector, but also in trade, hospitality, transport, and communication sectors. Meat from hunts is also often donated or sold to nearby communities and can be highly valued locally.  Additionally, revenue from trophy hunting can be reinvested to fund community needs, including:  - education  - sanitation  - development of infrastructure  As an example, in 2018 it was estimated that trophy hunting contributes more than US$341 million to the South African economy each year. It was also estimated that the trophy hunting industry supports more than 17,000 jobs across the country. The agricultural sector benefits the most, which is helpful for rural development and poverty alleviation goals." |
