# Supplementary material for: Exploring the efficacy of identity priming and message framing in influencing American attitudes toward trophy hunting
Source: PLoS One. 2024 Nov 7;19(11):e0312949. doi: 10.1371/journal.pone.0312949 (PMC11542780; doi:10.1371/journal.pone.0312949)
Supplement: S2 Appendix — (DOCX) [file pone.0312949.s002.docx]

**S2 Appendix. Example survey version (Treatment 3: control message and values prime)**

Q1. Are you a resident of the United States?

- Yes
- No

Now, we'll ask you about your thoughts specifically related to trophy hunting.

Q2. Please indicate your level of approval for legal, regulated trophy hunting as of right now.

- Strongly Disapprove
- Somewhat Disapprove
- Neither Approve Nor Disapprove
- Somewhat Approve
- Strongly Approve
- I Don't Know

We'll now ask you a bit more about yourself and what is important to you, followed by a few more questions related to trophy hunting.

Q3. For each of the areas listed below, consider how you would most like for people (including yourself) to live. Then, drag and drop to rank the importance of each area, with 1 being the most important.

______ Family/Friendship: Spending time with close relatives and loved ones

______ Conservation/Stewardship: Taking care of Earth and nature

______ Economic/Financial Success: Ensuring that people have enough money to live well

______ Growth and Learning: Having the opportunity to learn new skills or gain new knowledge

______ Recreation/Fun: Having time for enjoyment and leisure activities

Well-managed trophy hunting can be defined as recreational hunting that involves the limited, regulated harvest of high-value wildlife species. The individual animals are usually chosen for their notable physical traits (e.g., large horns, tusks, or body size) for the purpose of displaying part or all of the hunted animal. Trophy hunters pay substantial fees for the ability to hunt, usually in the company of a professional hunting guide. The trophy hunting industry is run by hunting operators who market and sell hunts to clients (often at international hunting conventions), lease or own hunting areas (often private or community-owned lands), and employ the requisite staff (e.g., professional hunters, trackers, drivers, skinners, and camp staff).

Q4. Please indicate your level of agreement or disagreement with the following statements. 
**"The message that I just read..."**

|  | Strongly Disagree | Somewhat  Disagree | Neither Agree Nor Disagree | Somewhat  Agree | Strongly  Agree | I Don't  Know |
| --- | --- | --- | --- | --- | --- | --- |
| …is aligned with my personal views |  |  |  |  |  |  |
| …makes me feel like trophy hunting can be good for conservation |  |  |  |  |  |  |
| …makes me feel like I wanted to "argue back" to what was stated in the message |  |  |  |  |  |  |
| …addressed my concerns about trophy hunting |  |  |  |  |  |  |
| …provided factual evidence about the benefits of trophy hunting |  |  |  |  |  |  |
| …makes me feel like trophy hunting can be good for local people |  |  |  |  |  |  |
| …is well-reasoned |  |  |  |  |  |  |

Q5. Please indicate your level of approval for legal, regulated trophy hunting after reading that message.

- Strongly Disapprove
- Somewhat Disapprove
- Neither Approve Nor Disapprove
- Somewhat Approve
- Strongly Approve
- I Don't Know

Q6. Would you be more or less likely to trust the message you just saw if it was published by...

|  | Less Likely | Neither More Nor Less Likely | More Likely | I Don't Know |
| --- | --- | --- | --- | --- |
| The U.S. Fish and Wildlife Service |  |  |  |  |
| The South African Department of Environmental Affairs |  |  |  |  |
| Safari Club International |  |  |  |  |
| World Wildlife Fund (WWF) |  |  |  |  |
| The Nature Conservancy |  |  |  |  |
| A scientific journal |  |  |  |  |

This final section will ask you a few demographic questions that will only be used for statistical and comparative purposes.

Q7. In what type of setting is your main residence?

- Urban
- Suburban
- Semi-rural
- Rural

Q8. In what region do you currently reside?

- Northeast
- Southeast
- Midwest
- West

Q9. What is your age?

_________________________________________________________

Q10. What is your gender?

- Man
- Woman
- Other

Q11. What is your race? (Select all that apply.)

- White (Non-Hispanic)
- Black or African American
- Hispanic/Latino
- American Indian or Alaska Native
- Asian
- Native Hawaiian or Pacific Islander
- Other

Q12. Which of the following best describes  your political alignment?

- Strongly Conservative
- Moderately Conservative
- Centrist
- Moderately Liberal
- Strongly Liberal

Q13. What is the highest degree or level of school you have completed?

- Less than 12th grade
- High school graduate/GED
- Some college/associate or technical degree
- Bachelor’s degree
- Graduate degree

Q14. Which of these categories best describes your total household income?

- Under $20,000
- $20,000 - $39,999
- $40,000 - $59,999
- $60,000 - $79,999
- $80,000 - $99,999
- $100,000 - $119,999
- $120,000 or More
- Do Not Wish to Answer

Q15. Thank you for participating in this survey! Do you have any comments you wish to add?

________________________________________________________________

________________________________________________________________
