# Supplementary material for: Exploring the efficacy of identity priming and message framing in influencing American attitudes toward trophy hunting
Source: PLoS One. 2024 Nov 7;19(11):e0312949. doi: 10.1371/journal.pone.0312949 (PMC11542780; doi:10.1371/journal.pone.0312949)
Supplement: S3 Appendix — (DOCX) [file pone.0312949.s003.docx]

**S3 Appendix. Results of Nonparametric Tests**

**Table 1. Descriptive statistics and Kruskal-Wallis results showing identity-based differences in approval for trophy hunting**

| **Characteristic** | **% of respondents** | **Median approval for**  **trophy hunting** | **Characteristic** | **% of respondents** | **Median approval for**  **trophy hunting** |
| --- | --- | --- | --- | --- | --- |
| ***Political alignment***  (*H* = 74.19***) |  |  | ***Economic/financial success rank***  (*H* = 11.13*) |  |  |
| Strongly conservative | 13.00 | 3.00a | 1 | 20.35 | 2.00a |
| Moderately conservative | 26.80 | 3.00ab | 2 | 22.35 | 2.00a |
| Centrist | 27.25 | 2.00b | 3 | 21.80 | 2.00a |
| Moderately liberal | 21.95 | 2.00c | 4 | 17.15 | 2.00a |
| Strongly liberal | 11.00 | 1.00c | 5 | 18.35 | 2.00a |
| ***Family/friendship rank***  (*H* = 4.74) |  |  | ***Growth and learning rank***  (*H* = 9.33) |  |  |
| 1 | 45.00 | 2.00a | 1 | 12.20 | 2.00a |
| 2 | 20.70 | 2.00a | 2 | 24.05 | 2.00a |
| 3 | 13.75 | 2.00a | 3 | 27.50 | 2.00a |
| 4 | 12.20 | 2.00a | 4 | 21.10 | 2.00a |
| 5 | 8.35 | 3.00a | 5 | 15.15 | 2.00a |
| ***Conservation/stewardship rank***  ***(****H* = 26.45***) |  |  | ***Recreation/fun rank***  (*H* = 29.53***) |  |  |
| 1 | 15.45 | 2.00a | 1 | 7.00 | 3.00a |
| 2 | 17.75 | 2.00a | 2 | 15.15 | 3.00ab |
| 3 | 18.70 | 2.00a | 3 | 18.25 | 2.00ab |
| 4 | 22.80 | 2.00ab | 4 | 26.75 | 2.00bc |
| 5 | 25.30 | 3.00b | 5 | 32.85 | 2.00c |

^a^Medians sharing a letter are not significantly different at α = 0.05

^b^* indicates significance at *p* < 0.05, *** indicates significance at p < 0.001

^c^Scale is 1 = “Strongly disapprove” to 5 = “Strongly approve

**Table 2. Kruskal-Wallis results showing differences in response to induction checks by treatment**

|  | **Treatment group** | | | | | | | | | |
| --- | --- | --- | --- | --- | --- | --- | --- | --- | --- | --- |
|  | 1. No prime control | 2. Social identity control | 3.Values control | 4. No prime wildlife | 5. Social identity wildlife | 6. Values wildlife | 7. No prime socioeconomic | 8. Social identity socioeconomic | 9. Values socioeconomic | All respondents |
| **Induction check statement** | **Median agreement** | | | | | | | | | |
| *The message that I just read...* |  |  |  |  |  |  |  |  |  |  |
| is aligned with my personal views. | 2.00a | 3.00ab | 2.00a | 3.00bcd | 3.00cd | 3.00c | 3.00bcd | 3.00ad | 3.00ad | 3.00 |
| makes me feel like trophy hunting can be good for conservation | 3.00a | 3.00ab | 3.00ab | 4.00c | 3.00c | 3.00c | 3.00c | 3.00bc | 3.00abc | 3.00 |
| makes me feel like I wanted to "argue back" to what was stated in the message | 3.00ab | 3.00a | 3.00ab | 3.00b | 3.00b | 3.00ab | 3.00ab | 3.00ab | 3.00ab | 3.00 |
| addressed my concerns about trophy hunting | 3.00ab | 3.00ab | 3.00a | 3.00abc | 3.00bc | 4.00c | 3.00abc | 3.00abc | 3.00abc | 3.00 |
| provided factual evidence about the benefits of trophy hunting | 3.00ab | 3.00abc | 3.00a | 3.00c | 3.00c | 3.00c | 3.00c | 3.00bc | 3.00abc | 3.00 |
| makes me feel like trophy hunting can be good for local people | 3.00a | 3.00ab | 3.00a | 3.00c | 3.00c | 3.00c | 3.00c | 3.00c | 3.00bc | 3.00 |
| is well-reasoned | 3.00a | 3.00abc | 3.00ac | 3.00d | 3.00d | 3.00d | 3.00bd | 3.00bd | 3.00bcd | 3.00 |

^a^Medians sharing a letter are not significant different at α = 0.05

^b^Scale is 1 = “Strongly disagree” to 5 = “Strongly agree”

**Table 3. Kruskal-Wallis and paired Wilcoxon signed-rank results showing treatment effect on pre- and post-message approval for trophy hunting**

| **Treatment** | **n** | **Pre-test median** | **Post-test median** | ***V* statistic** | **r_B_** |
| --- | --- | --- | --- | --- | --- |
| ***All respondents*** |  | *H* = 11.17 | *H* = 50.82*** |  |  |
| 1. No prime control | 226 | 2.00a | 2.00a | 2303.50 | -0.07 |
| 2. Social identity control | 225 | 3.00a | 2.00ab | 1434.00 | -0.07 |
| 3. Values control | 227 | 2.00a | 3.00ac | 1779.00* | -0.24 |
| 4. No prime wildlife | 227 | 2.00a | 3.00d | 897.50*** | -0.72 |
| 5. Social identity wildlife | 231 | 3.00a | 3.00bd | 1307.00*** | -0.53 |
| 6. Values wildlife | 227 | 2.00a | 3.00d | 1331.50*** | -0.64 |
| 7. No prime socioeconomic | 203 | 2.00a | 3.00bcd | 965.50*** | -0.72 |
| 8. Social identity socioeconomic | 224 | 2.00a | 3.00bcd | 1460.00*** | -0.55 |
| 9. Values socioeconomic | 210 | 2.00a | 3.00abcd | 1348.50*** | -0.51 |
| ***Conservative respondents only*** |  | *H* = 4.30 | *H* = 17.76* |  |  |
| 1. No prime control | 82 | 3.00a | 3.00a | 403.00 | -0.06 |
| 2. Social identity control | 97 | 3.00a | 2.00a | 265.50 | -0.11 |
| 3. Values control | 88 | 3.00a | 3.00a | 231.00* | -0.44 |
| 4. No prime wildlife | 91 | 3.00a | 4.00a | 44.00*** | -0.89 |
| 5. Social identity wildlife | 89 | 3.00a | 3.00a | 118.50** | -0.60 |
| 6. Values wildlife | 91 | 3.00a | 3.00a | 340.50** | -0.51 |
| 7. No prime socioeconomic | 89 | 2.00a | 3.00a | 183.50*** | -0.69 |
| 8. Social identity socioeconomic | 89 | 3.00a | 3.00a | 288.00* | -0.42 |
| 9. Values socioeconomic | 80 | 2.50a | 3.00a | 234.50** | -0.48 |
| ***Liberal respondents only*** |  | *H* = 9.06 | *H* = 22.41** |  |  |
| 1. No prime control | 92 | 2.00a | 2.00ab | 292.00 | -0.02 |
| 2. Social identity control | 73 | 2.00a | 3.00ab | 147.50 | -0.16 |
| 3. Values control | 78 | 2.00a | 2.00a | 191.50 | -0.12 |
| 4. No prime wildlife | 65 | 2.00a | 2.00ab | 72.00** | -0.67 |
| 5. Social identity wildlife | 69 | 2.00a | 3.00ab | 144.00** | -0.59 |
| 6. Values wildlife | 72 | 2.00a | 3.00ab | 57.00*** | -0.83 |
| 7. No prime socioeconomic | 60 | 2.00a | 3.00b | 92.00*** | -0.76 |
| 8. Social identity socioeconomic | 74 | 2.00a | 3.00ab | 104.00*** | -0.65 |
| 9. Values socioeconomic | 76 | 1.50a | 2.00ab | 131.00** | -0.58 |
| ***High conservation only*** |  | *H* = 2.01 | *H* = 38.74*** |  |  |
| 1. No prime control | 78 | 2.00a | 2.00a | 265.50 | 0.22 |
| 2. Social identity control | 69 | 2.00a | 2.00abc | 162.50 | -0.14 |
| 3. Values control | 84 | 2.00a | 2.00ac | 199.00 | -0.02 |
| 4. No prime wildlife | 65 | 2.00a | 3.00b | 37.50*** | -0.89 |
| 5. Social identity wildlife | 74 | 2.00a | 3.00bc | 102.00*** | -0.69 |
| 6. Values wildlife | 73 | 2.00a | 3.00bc | 256.00** | -0.51 |
| 7. No prime socioeconomic | 67 | 2.00a | 3.00bc | 130.00*** | -0.65 |
| 8. Social identity socioeconomic | 80 | 2.00a | 3.00abc | 190.00** | -0.49 |
| 9. Values socioeconomic | 74 | 2.00a | 3.00abc | 179.00* | -0.43 |
| ***High economic only*** |  | *H* = 10.89 | *H* = 20.35** |  |  |
| 1. No prime control | 98 | 3.00a | 3.00ab | 615.00 | -0.07 |
| 2. Social identity control | 109 | 2.00a | 3.00ab | 405.50 | -0.10 |
| 3. Values control | 92 | 2.00a | 2.00a | 306.00 | -0.32 |
| 4. No prime wildlife | 95 | 3.00a | 3.00ab | 182.00*** | -0.65 |
| 5. Social identity wildlife | 85 | 2.00a | 3.00ab | 265.50** | -0.46 |
| 6. Values wildlife | 92 | 2.50a | 3.00b | 199.00*** | -0.70 |
| 7. No prime socioeconomic | 93 | 2.00a | 3.00ab | 217.50*** | -0.75 |
| 8. Social identity socioeconomic | 104 | 2.50a | 3.00ab | 293.50*** | -0.57 |
| 9. Values socioeconomic | 86 | 2.00a | 3.00ab | 155.00*** | -0.62 |

^a^Medians sharing a letter are not significantly different at α = 0.05

^b^* indicates significance at *p* < 0.05, ** indicates significance at *p* < 0.01, *** indicates significance at *p* < 0.001

^c^Scale is 1 = “Strongly disapprove” to 5 = “Strongly approve”

^d^“High conservation” refers to respondents who ranked “Conservation/Stewardship” as one of their top two personal values; “High economic” refers to respondents who ranked “Economic/Financial success” as one of their top two personal values

**Table 4. Descriptive statistics and Kruskal-Wallis results showing differences in median likelihood of trust in message sources**

| **Message source**  (*H* = 589.70***) | **Less likely** | **Neither more nor less likely** | **More likely** | **I don't know** | **Median trust** |
| --- | --- | --- | --- | --- | --- |
| A scientific journal | 10.86% | 36.74% | 41.99% | 10.41% | 2.00a |
| South African Department of Environmental Affairs (SADEA) | 18.47% | 40.34% | 30.13% | 11.06% | 2.00b |
| Safari Club International (SCI) | 24.90% | 39.80% | 23.30% | 12.00% | 2.00c |
| The Nature Conservancy (TNC) | 9.00% | 32.47% | 48.72% | 9.80% | 3.00d |
| U.S. Fish and Wildlife Service (USFWS) | 10.60% | 34.50% | 47.15% | 7.75% | 3.00ad |
| World Wildlife Fund (WWF) | 10.72% | 31.45% | 48.77% | 9.06% | 3.00d |

^a^Medians sharing a letter are not significantly different at α = 0.05

^b^Scale is 1 = “Less likely” to 3 = “More likely”
