# Supplementary material for: Exploring the efficacy of identity priming and message framing in influencing American attitudes toward trophy hunting
Source: PLoS One. 2024 Nov 7;19(11):e0312949. doi: 10.1371/journal.pone.0312949 (PMC11542780; doi:10.1371/journal.pone.0312949)
Supplement: S5 Appendix — (PDF) [file pone.0312949.s005.pdf]

```
#S5 Appendix. Exploring the efficacy of identity priming and message framing in
#influencing American attitudes toward trophy hunting R code for analyses
#Required packages "readxl" "effsize" "effectsize" "car" "rstatix"
setwd("")
library(readxl)
THData <- read_xlsx("TH Data Sheet Labelled.xlsx", sheet = "Sheet1")
head(THData)

###Demographics Summary Stats

##Age
Age <- THData$Age
Age
table(Age)
length(Age)
mean(Age, na.rm = TRUE)

####Fixing Age into 3 Categories
##Sort Age into bins: 17-34, 35-54, 55+
THData["age_group"] = cut(THData$Age, c(17, 34, 54, Inf),
                          c("1", "2", "3"), include.lowest=TRUE)
AgeFix <- THData$age_group
AgeFix
length(AgeFix)
table(AgeFix)

###Income
Income <- THData$Income
Income
table(Income)
length(Income)

###Gender
##Need to Remove 3 from Gender
Gender <- THData$Gender
Gender
table(Gender)
THData$Gender[THData$Gender == 3] <- NA
THData
Gender <- THData$Gender
Gender
table(Gender)

###Region
Region <- THData$Region
Region
table(Region)

###Setting
Setting <- THData$Setting
Setting
table(Setting)

###Education
Education <- THData$Education
Education
table(Education)

###Race/Ethnicity
###Race Adjustments
THData$Race <- paste(THData$RaceWhite, THData$RaceBlack, THData$RaceLatino,
                     THData$RaceNative, THData$RaceAsian, THData$RaceHawaiianPI,
                     THData$RaceOther, sep = '-')
```

```
Race <- THData$Race
Race
table(Race)
####Sort Race Into Bins
THData$RaceBin <- ifelse(THData$Race == '1-NA-NA-NA-NA-NA-NA', 'White Alone',
ifelse(THData$Race == 'NA-1-NA-NA-NA-NA-NA', 'Black Alone',
ifelse(THData$Race == 'NA-NA-1-NA-NA-NA-NA', 'Latino Alone',
ifelse(THData$Race == 'NA-NA-NA-1-NA-NA-NA', 'AmInd Alone',
ifelse(THData$Race == 'NA-NA-NA-NA-1-NA-NA', 'Asian Alone',
ifelse(THData$Race == 'NA-NA-NA-NA-NA-1-NA', 'NatHawaii Alone',
ifelse(THData$Race == 'NA-NA-NA-NA-NA-NA-1', 'Other Alone', 'Two or More')))))))
RaceBinned <- THData$RaceBin
RaceBinned
table(RaceBinned)


#####Induction Statement Testing

#####Across Groups

##Personal Views
PersView <- THData$MCViews
PersView
table(PersView)
length(PersView)
propPers<- table(PersView)/sum(table(PersView))
perPers <- propPers*100
perPers
## Remove 6
THData$MCViews[THData$MCViews == 6] <- 3
THData$MCViews <- as.numeric(THData$MCViews)
PersView <- THData$MCViews
PersView
table(PersView)
length(PersView)
mean(PersView, na.rm = TRUE)
sd(PersView, na.rm = TRUE)
median(PersView, na.rm = TRUE)

##Test Differences Across Messages
###Get Personal Views By Version
table(THData$MCViews, THData$Version)
aggregate(MCViews ~ Version, data = THData, FUN = mean)
aggregate(MCViews ~ Version, data = THData, FUN = sd)

##ANOVA Model
PersViewMod <- aov(MCViews ~ Version, data = THData)
summary(PersViewMod)

library(effectsize)
eta_squared(PersViewMod, partial = FALSE)

##Bonferroni
pairwise.t.test(THData$MCViews, THData$Version, p.adj = "bonf")

##Make table with Bonf significance
PersViewApp <- aggregate(MCViews ~ Version, data = THData, FUN = mean)
PersViewApp
#define new column
BonSigPersView <- c('a','ab','a', 'bcd', 'ce', 'cfg', 'bef', 'ade', 'adeg')
#add column
PersViewSig <- cbind(PersViewApp, BonSigPersView)
PersViewSig

## Check normality of the residuals with the Shapiro-Wilk test
```

```
shapiro.test(PersViewMod$residuals)
###Check for equality of variance
library(car)
leveneTest(MCViews ~Version,
           data = THData)

###Get Personal Views By Version
aggregate(MCViews ~ Version, data = THData, FUN = median)

##Kruskal Wallis Test
library(rstatix)
kruskal.test(MCViews ~ Version, data = THData)

# pairwise using dunn test
DunnPersView <- dunn_test(MCViews ~ Version, data = THData,
                          p.adjust = "bonferroni")
DunnPersView
print(DunnPersView, n = 36)

##Make table with Bonf significance
NPPersViewApp <- aggregate(MCViews ~ Version, data = THData, FUN = median)
NPPersViewApp
#define new column to add
NPBonSigPersView <- c('a','ab','a', 'bcd', 'cd', 'c', 'bcd', 'ad', 'ad')
#add column called 'new'
NPPersViewSig <- cbind(NPPersViewApp, NPBonSigPersView)
#view new data frame
NPPersViewSig

##Good for Conservation
Goodcon <- THData$MCConservation
Goodcon
table(Goodcon)
length(Goodcon)
propCon<- table(Goodcon)/sum(table(Goodcon))
perCon <- propCon*100
perCon
## Remove 6
THData$MCConservation[THData$MCConservation == 6] <- 3
THData$MCConservation <- as.numeric(THData$MCConservation)
Goodcon <- THData$MCConservation
table(Goodcon)
length(Goodcon)
mean(Goodcon, na.rm = TRUE)
sd(Goodcon, na.rm = TRUE)
median(Goodcon, na.rm = TRUE)
aggregate(MCConservation ~ Version, data = THData, FUN = mean)
aggregate(MCConservation ~ Version, data = THData, FUN = sd)
ggregate(MCConservation ~ Version, data = THData, FUN = median)

##ANOVA Model
GoodConMod <- aov(MCConservation ~ Version, data = THData)
summary(GoodConMod)

library(effectsize)
eta_squared(GoodConMod, partial = FALSE)

##Bonferroni
pairwise.t.test(THData$MCConservation, THData$Version, p.adj = "bonf")

##Make table with Bonf significance
GoodConApp <- aggregate(MCConservation ~ Version, data = THData, FUN = mean)
GoodConApp
#define new column
BonSigGoodCon <- c('a','ab','ab', 'c', 'c', 'c', 'c', 'bc', 'abc')
```

```
#add column
GoodConSig <- cbind(GoodConApp, BonSigGoodCon)
GoodConSig

## Check normality of the residuals with the Shapiro-Wilk test
shapiro.test(GoodConMod$residuals)
###Check for equality of variance
library(car)
leveneTest(MCConservation ~Version,
           data = THData)

##Kruskal Wallis Test
library(rstatix)
kruskal.test(MCConservation ~ Version, data = THData)

# pairwise using dunn test
DunnGoodCon <- dunn_test(MCConservation ~ Version, data = THData,
                         p.adjust = "bonferroni")
DunnGoodCon
print(DunnGoodCon, n = 36)

##Make table with Bonf significance
NPGoodConApp <- aggregate(MCConservation ~ Version, data = THData, FUN = median)
NPGoodConApp
#define new column to add
NPBonSigGoodCon <- c('a','ab','ab', 'c', 'c', 'c', 'c', 'bc', 'abc')
#add column called 'new'
NPGoodConSig <- cbind(NPGoodConApp, NPBonSigGoodCon)
#view new data frame
NPGoodConSig

##Argue Back
Argue <- THData$MCArgue
Argue
table(Argue)
length(Argue)
propArg<- table(Argue)/sum(table(Argue))
perArg <- propArg*100
perArg
## Remove 6
THData$MCArgue[THData$MCArgue == 6] <- 3
THData$MCArgue <- as.numeric(THData$MCArgue)
Argue <- THData$MCArgue
Argue
table(Argue)
length(Argue)
mean(Argue, na.rm = TRUE)
sd(Argue, na.rm = TRUE)
median(Argue, na.rm = TRUE)
aggregate(MCArgue ~ Version, data = THData, FUN = mean)
aggregate(MCArgue ~ Version, data = THData, FUN = sd)
aggregate(MCArgue ~ Version, data = THData, FUN = median)

##ANOVA Model
ArgueMod <- aov(MCArgue ~ Version, data = THData)
summary(ArgueMod)

library(effectsize)
eta_squared(ArgueMod, partial = FALSE)

##Bonferroni
pairwise.t.test(THData$MCArgue, THData$Version, p.adj = "bonf")

##Make table with Bonf significance
ArgueApp <- aggregate(MCArgue ~ Version, data = THData, FUN = mean)
```

```
ArgueApp
#define new column
BonSigArgue <- c('ab','a','ab', 'b', 'b', 'ab', 'ab', 'ab', 'ab')
#add column
ArgueSig <- cbind(ArgueApp, BonSigArgue)
ArgueSig

## Check normality of the residuals with the Shapiro-Wilk test
shapiro.test(ArgueMod$residuals)
###Check for equality of variance
library(car)
leveneTest(MCArgue ~Version,
           data = THData)

##Kruskal Wallis Test
library(rstatix)
kruskal.test(MCArgue ~ Version, data = THData)

# pairwise using dunn test
DunnArgue <- dunn_test(MCArgue ~ Version, data = THData,
                       p.adjust = "bonferroni")
DunnArgue
print(DunnArgue, n = 36)

##Make table with Bonf significance
NPArgueApp <- aggregate(MCArgue ~ Version, data = THData, FUN = median)
NPArgueApp
#define new column to add
NPBonSigArgue <- c('ab','a','ab', 'b', 'b', 'ab', 'ab', 'ab', 'ab')
#add column called 'new'
NPArgueSig <- cbind(NPArgueApp, NPBonSigArgue)
#view new data frame
NPArgueSig

##Addressed Concerns
Concern <- THData$MCConcern
Concern
table(Concern)
length(Concern)
propConcern<- table(Concern)/sum(table(Concern))
perConcern <- propConcern*100
perConcern
## Remove 6
THData$MCConcern[THData$MCConcern == 6] <- 3
THData$MCConcern <- as.numeric(THData$MCConcern)
Concern <- THData$MCConcern
Concern
table(Concern)
length(Concern)
mean(Concern, na.rm = TRUE)
sd(Concern, na.rm = TRUE)
median(Concern, na.rm = TRUE)
aggregate(MCConcern ~ Version, data = THData, FUN = mean)
aggregate(MCConcern ~ Version, data = THData, FUN = sd)
aggregate(MCConcern ~ Version, data = THData, FUN = median)

##ANOVA Model
ConcernMod <- aov(MCConcern ~ Version, data = THData)
summary(ConcernMod)

library(effectsize)
eta_squared(ConcernMod, partial = FALSE)

##Bonferroni
pairwise.t.test(THData$MCConcern, THData$Version, p.adj = "bonf")
```

```
##Make table with Bonf significance
ConcernApp <- aggregate(MCConcern ~ Version, data = THData, FUN = mean)
ConcernApp
#define new column
BonSigConcern <- c('ab','ab','a', 'abc', 'bc', 'c', 'abc', 'abc', 'abc')
#add column
ConcernSig <- cbind(ConcernApp, BonSigConcern)
ConcernSig

## Check normality of the residuals with the Shapiro-Wilk test
shapiro.test(ConcernMod$residuals)
###Check for equality of variance
library(car)
leveneTest(MCConcern ~Version,
           data = THData)

##Kruskal Wallis Test
library(rstatix)
kruskal.test(MCConcern ~ Version, data = THData)

# pairwise using dunn test
DunnConcern <- dunn_test(MCConcern ~ Version, data = THData,
                        p.adjust = "bonferroni")
DunnConcern
print(DunnConcern, n = 36)

##Make table with Bonf significance
NPConcernApp <- aggregate(MCConcern ~ Version, data = THData, FUN = median)
NPConcernApp
#define new column to add
NPBonSigConcern <- c('ab','ab','a', 'abc', 'bc', 'c', 'abc', 'abc', 'abc')
#add column called 'new'
NPConcernSig <- cbind(NPConcernApp, NPBonSigConcern)
#view new data frame
NPConcernSig

##Factual Evidence
Factevd <- THData$MCFacts
Factevd
table(Factevd)
length(Factevd)
propFact<- table(Factevd)/sum(table(Factevd))
perFact <- propFact*100
perFact
## Remove 6
THData$MCFacts[THData$MCFacts == 6] <- 3
THData$MCFacts <- as.numeric(THData$MCFacts)
Factevd <- THData$MCFacts
Factevd
table(Factevd)
length(Factevd)
mean(Factevd, na.rm = TRUE)
sd(Factevd, na.rm = TRUE)
median(Factevd, na.rm = TRUE)
aggregate(MCFacts ~ Version, data = THData, FUN = mean)
aggregate(MCFacts ~ Version, data = THData, FUN = sd)
aggregate(MCFacts ~ Version, data = THData, FUN = median)

##ANOVA Model
FactMod <- aov(MCFacts ~ Version, data = THData)
summary(FactMod)

library(effectsize)
eta_squared(FactMod, partial = FALSE)
```

```
##Bonferroni
pairwise.t.test(THData$MCFacts, THData$Version, p.adj = "bonf")

##Make table with Bonf significance
FactApp <- aggregate(MCFacts ~ Version, data = THData, FUN = mean)
FactApp
#define new column
BonSigFact <- c('ab','abc','ad', 'c', 'c', 'c', 'c', 'bc', 'bcd')
#add column
FactSig <- cbind(FactApp, BonSigFact)
FactSig

## Check normality of the residuals with the Shapiro-Wilk test
shapiro.test(FactMod$residuals)
###Check for equality of variance
library(car)
leveneTest(MCFacts ~Version,
           data = THData)

##Kruskal Wallis Test
library(rstatix)
kruskal.test(MCFacts ~ Version, data = THData)

# pairwise using dunn test
DunnFact <- dunn_test(MCFacts ~ Version, data = THData,
                      p.adjust = "bonferroni")
DunnFact
print(DunnFact, n = 36)

##Make table with Bonf significance
NPFactApp <- aggregate(MCFacts ~ Version, data = THData, FUN = median)
NPFactApp
#define new column to add
NPBonSigFact <- c('ab','abc','a', 'c', 'c', 'c', 'c', 'bc', 'abc')
#add column called 'new'
NPFactSig <- cbind(NPFactApp, NPBonSigFact)
#view new data frame
NPFactSig

##Good for Local People
Localp <- THData$MCLocal
Localp
table(Localp)
length(Localp)
propLoc<- table(Localp)/sum(table(Localp))
perLoc <- propLoc*100
perLoc
## Remove 6
THData$MCLocal[THData$MCLocal == 6] <- 3
THData$MCLocal <- as.numeric(THData$MCLocal)
Localp <- THData$MCLocal
Localp
table(Localp)
length(Localp)
mean(Localp, na.rm = TRUE)
sd(Localp, na.rm = TRUE)
median(Localp, na.rm = TRUE)
aggregate(MCLocal ~ Version, data = THData, FUN = mean)
aggregate(MCLocal ~ Version, data = THData, FUN = sd)
aggregate(MCLocal ~ Version, data = THData, FUN = median)

##ANOVA Model
LocalMod <- aov(MCLocal ~ Version, data = THData)
summary(LocalMod)
```

```
library(effectsize)
eta_squared(LocalMod, partial = FALSE)

##Bonferroni
pairwise.t.test(THData$MCLocal, THData$Version, p.adj = "bonf")

##Make table with Bonf significance
LocalApp <- aggregate(MCLocal ~ Version, data = THData, FUN = mean)
LocalApp
#define new column
BonSigLocal <- c('a','ab','a', 'c', 'c', 'c', 'c', 'c', 'bc')
#add column
LocalSig <- cbind(LocalApp, BonSigLocal)
LocalSig

## Check normality of the residuals with the Shapiro-Wilk test
shapiro.test(LocalMod$residuals)
###Check for equality of variance
library(car)
leveneTest(MCLocal ~Version,
           data = THData)

##Kruskal Wallis Test
library(rstatix)
kruskal.test(MCLocal ~ Version, data = THData)

# pairwise using dunn test
DunnLocal <- dunn_test(MCLocal ~ Version, data = THData,
                       p.adjust = "bonferroni")
DunnLocal
print(DunnLocal, n = 36)

##Make table with Bonf significance
NPLocalApp <- aggregate(MCLocal ~ Version, data = THData, FUN = median)
NPLocalApp
#define new column to add
NPBonSigLocal <- c('a','ab','a', 'c', 'c', 'c', 'c', 'c', 'bc')
#add column called 'new'
NPLocalSig <- cbind(NPLocalApp, NPBonSigLocal)
#view new data frame
NPLocalSig

##Well-Reasoned
WellRea <- THData$MCReason
WellRea
table(WellRea)
length(WellRea)
propWell<- table(WellRea)/sum(table(WellRea))
perWell <- propWell*100
perWell
## Remove 6
THData$MCReason[THData$MCReason == 6] <- 3
THData$MCReason <- as.numeric(THData$MCReason)
WellRea <- THData$MCReason
WellRea
table(WellRea)
length(WellRea)
mean(WellRea, na.rm = TRUE)
sd(WellRea, na.rm = TRUE)
median(WellRea, na.rm = TRUE)
aggregate(MCReason ~ Version, data = THData, FUN = mean)
aggregate(MCReason ~ Version, data = THData, FUN = sd)
aggregate(MCReason ~ Version, data = THData, FUN = median)
```

```
##ANOVA Model
ReasonMod <- aov(MCReason ~ Version, data = THData)
summary(ReasonMod)

library(effectsize)
eta_squared(ReasonMod, partial = FALSE)

##Bonferroni
pairwise.t.test(THData$MCReason, THData$Version, p.adj = "bonf")

##Make table with Bonf significance
ReasonApp <- aggregate(MCReason ~ Version, data = THData, FUN = mean)
ReasonApp
#define new column
BonSigReason <- c('a','ab','ac', 'd', 'd', 'd', 'd', 'bd', 'bcd')
#add column
ReasonSig <- cbind(ReasonApp, BonSigReason)
ReasonSig

## Check normality of the residuals with the Shapiro-Wilk test
shapiro.test(ReasonMod$residuals)
###Check for equality of variance
library(car)
leveneTest(MCReason ~Version,
           data = THData)

##Kruskal Wallis Test
library(rstatix)
kruskal.test(MCReason ~ Version, data = THData)

# pairwise using dunn test
DunnReason <- dunn_test(MCReason ~ Version, data = THData,
                        p.adjust = "bonferroni")
DunnReason
print(DunnReason, n = 36)

##Make table with Bonf significance
NPReasonApp <- aggregate(MCReason ~ Version, data = THData, FUN = median)
NPReasonApp
#define new column to add
NPBonSigReason <- c('a','abc','ac', 'd', 'd', 'd', 'bd', 'bd', 'bcd')
#add column called 'new'
NPReasonSig <- cbind(NPReasonApp, NPBonSigReason)
#view new data frame
NPReasonSig

########Difference in TH Attitudes Pre and Post Message

######Pre Message

###Approval Pre Message Summary
table(THData$THPreTot)
length(THData$THPreTot)
propPreAll<- table(THData$THPreTot)/sum(table(THData$THPreTot))
perPreAll <- propPreAll*100
perPreAll
THData$THPreTot <- as.numeric(THData$THPreTot)

#Get TH Approval by Message
table(THData$THPreTot, THData$Version)
aggregate(THPreTot ~ Version, data = THData, FUN = mean)

## Remove 6 From Trophy Hunting Pre
THData$THPreTot[THData$THPreTot == 6] <- 3
table(THData$THPreTot)
```

```
#Get TH Approval by Message
table(THData$THPreTot, THData$Version)
aggregate(THPreTot ~ Version, data = THData, FUN = mean)
aggregate(THPreTot ~ Version, data = THData, FUN = sd)
aggregate(THPreTot ~ Version, data = THData, FUN = median)
mean(THData$THPreTot, na.rm = TRUE)
sd(THData$THPreTot, na.rm = TRUE)
median(THData$THPreTot, na.rm = TRUE)

##Determine Differences by Message Group
##ANOVA Model
PreMesMod <- aov(THPreTot ~ Version, data = THData)
summary(PreMesMod)

library(effectsize)
eta_squared(PreMesMod, partial = FALSE)

## Check normality of the residuals with the Shapiro-Wilk test
shapiro.test(PreMesMod$residuals)
###Check for equality of variance
library(car)
leveneTest(THPreTot ~Version,
           data = THData)

##Kruskal Wallis Test
library(rstatix)
kruskal.test(THPreTot ~ Version, data = THData)

#####Post Message

###Approval Post Message Summary
table(THData$THPostTot)
length(THData$THPostTot)
propPostAll<- table(THData$THPostTot)/sum(table(THData$THPostTot))
perPostAll <- propPostAll*100
perPostAll
THData$THPostTot <- as.numeric(THData$THPostTot)
#Get TH Approval by Message
table(THData$THPostTot, THData$Version)
aggregate(THPostTot ~ Version, data = THData, FUN = mean)

## Remove 6 From Trophy Hunting Post
THData$THPostTot[THData$THPostTot == 6] <- 3
table(THData$THPostTot)
#Get TH Approval by Message
table(THData$THPostTot, THData$Version)
aggregate(THPostTot ~ Version, data = THData, FUN = mean)
aggregate(THPostTot ~ Version, data = THData, FUN = sd)
aggregate(THPostTot ~ Version, data = THData, FUN = median)
mean(THData$THPostTot, na.rm = TRUE)
sd(THData$THPostTot, na.rm = TRUE)
median(THData$THPostTot, na.rm = TRUE)

##Determine Differences by Message Group
##ANOVA Model
PostMesMod <- aov(THPostTot ~ Version, data = THData)
summary(PostMesMod)

library(effectsize)
eta_squared(PostMesMod, partial = FALSE)

##Bonferroni
pairwise.t.test(THData$THPostTot, THData$Version, p.adj = "bonf")

##Make table with Bonf significance
```

```
AllPostApp <- aggregate(THPostTot ~ Version, data = THData, FUN = mean)
AllPostApp
#define new column
BonSigAllPost <- c('a','ab','ac', 'd', 'bd', 'd', 'bcd', 'bcd', 'abcd')
#add column
AllPostSig <- cbind(AllPostApp, BonSigAllPost)
AllPostSig

## Check normality of the residuals with the Shapiro-Wilk test
shapiro.test(PostMesMod$residuals)
###Check for equality of variance
library(car)
leveneTest(THPostTot ~Version,
           data = THData)

##Test Differences Across Message Version
##Kruskal Wallis Test
library(rstatix)
kruskal.test(THPostTot ~ Version, data = THData)

# pairwise using dunn test
DunnAllPost <- dunn_test(THPostTot ~ Version, data = THData,
                         p.adjust = "bonferroni")
DunnAllPost
print(DunnAllPost, n = 36)

##Make table with Bonf significance
NPAllPostApp <- aggregate(THPostTot ~ Version, data = THData, FUN = median)
NPAllPostApp
#define new column to add
NPBonSigAllPost <- c('a','ab','ac', 'd', 'bd', 'd', 'bcd', 'bcd', 'abcd')
#add column called 'new'
NPAllPostSig <- cbind(NPAllPostApp, NPBonSigAllPost)
#view new data frame
NPAllPostSig

#########Difference across all groups
t.test(THData$THPreTot, THData$THPostTot, paired = TRUE)

#########Separated By Message Treatment
#Check distribution
table(THData$Version)
propVers<- table(THData$Version)/sum(table(THData$Version))
perVers <- propVers*100
perVers

##Message 1: Control Control
#Remove Other Versions
THDataCC<- subset(THData, Version != 2)
THDataCC<- subset(THDataCC, Version != 3)
THDataCC<- subset(THDataCC, Version != 4)
THDataCC<- subset(THDataCC, Version != 5)
THDataCC<- subset(THDataCC, Version != 6)
THDataCC<- subset(THDataCC, Version != 7)
THDataCC<- subset(THDataCC, Version != 8)
THDataCC<- subset(THDataCC, Version != 9)
table(THDataCC$Version)

#Get Pre and Post Values
###Approval Pre Message Summary
table(THDataCC$THPreTot)
length(THDataCC$THPreTot)
propPreCC<- table(THDataCC$THPreTot)/sum(table(THDataCC$THPreTot))
perPreCC <- propPreCC*100
perPreCC
```

```
## Remove 6 From Trophy Hunting Pre
THDataCC$THPreTot[THDataCC$THPreTot == 6] <- 3
THDataCC$THPreTot <- as.numeric(THDataCC$THPreTot)
table(THDataCC$THPreTot)
length(THDataCC$THPreTot)
mean(THDataCC$THPreTot, na.rm = TRUE)
sd(THDataCC$THPreTot, na.rm = TRUE)
median(THDataCC$THPreTot, na.rm = TRUE)

###Approval Post Message Summary
table(THDataCC$THPostTot)
length(THDataCC$THPostTot)
propPostCC<- table(THDataCC$THPostTot)/sum(table(THDataCC$THPostTot))
perPostCC <- propPostCC*100
perPostCC
## Remove 6 From Trophy Hunting Post
THDataCC$THPostTot[THDataCC$THPostTot == 6] <- 3
THDataCC$THPostTot <- as.numeric(THDataCC$THPostTot)
table(THDataCC$THPostTot)
mean(THDataCC$THPostTot, na.rm = TRUE)
sd(THDataCC$THPostTot, na.rm = TRUE)
median(THDataCC$THPostTot, na.rm = TRUE)

###Difference
t.test(THDataCC$THPreTot, THDataCC$THPostTot, paired = TRUE)
##Effect Size
library(effsize)
cohen.d(THDataCC$THPreTot, THDataCC$THPostTot, paired = TRUE, within = FALSE)

###Test Difference Using Wilcoxon Signed Rank Test
wilcox.test(THDataCC$THPreTot, THDataCC$THPostTot, paired = TRUE)

###Effect Size Rank Biserial Correlation
library(effectsize)
rank_biserial(THDataCC$THPreTot, THDataCC$THPostTot, paired = TRUE, ci = 0.95)

##Message 2: Social Identity Control
#Remove Other Versions
THDataSC<- subset(THData, Version != 1)
THDataSC<- subset(THDataSC, Version != 3)
THDataSC<- subset(THDataSC, Version != 4)
THDataSC<- subset(THDataSC, Version != 5)
THDataSC<- subset(THDataSC, Version != 6)
THDataSC<- subset(THDataSC, Version != 7)
THDataSC<- subset(THDataSC, Version != 8)
THDataSC<- subset(THDataSC, Version != 9)
table(THDataSC$Version)

#Get Pre and Post Values
###Approval Pre Message Summary
table(THDataSC$THPreTot)
length(THDataSC$THPreTot)
propPreSC<- table(THDataSC$THPreTot)/sum(table(THDataSC$THPreTot))
perPreSC <- propPreSC*100
perPreSC
## Remove 6 From Trophy Hunting Pre
THDataSC$THPreTot[THDataSC$THPreTot == 6] <- 3
THDataSC$THPreTot <- as.numeric(THDataSC$THPreTot)
table(THDataSC$THPreTot)
mean(THDataSC$THPreTot, na.rm = TRUE)
sd(THDataSC$THPreTot, na.rm = TRUE)
median(THDataSC$THPreTot, na.rm = TRUE)

###Approval Post Message Summary
table(THDataSC$THPostTot)
```

```
length(THDataSC$THPostTot)
propPostSC<- table(THDataSC$THPostTot)/sum(table(THDataSC$THPostTot))
perPostSC <- propPostSC*100
perPostSC
## Remove 6 From Trophy Hunting Post
THDataSC$THPostTot[THDataSC$THPostTot == 6] <- 3
THDataSC$THPostTot <- as.numeric(THDataSC$THPostTot)
table(THDataSC$THPostTot)
mean(THDataSC$THPostTot, na.rm = TRUE)
sd(THDataSC$THPostTot, na.rm = TRUE)
median(THDataSC$THPostTot, na.rm = TRUE)

###Difference
t.test(THDataSC$THPreTot, THDataSC$THPostTot, paired = TRUE)
##Effect Size
library(effsize)
cohen.d(THDataSC$THPreTot, THDataSC$THPostTot, paired = TRUE, within = FALSE)

###Test Difference Using Wilcoxon Signed Rank Test
wilcox.test(THDataSC$THPreTot, THDataSC$THPostTot, paired = TRUE)

###Effect Size Rank Biserial Correlation
library(effectsize)
rank_biserial(THDataSC$THPreTot, THDataSC$THPostTot, paired = TRUE, ci = 0.95)

##Message 3: Value Control
#Remove Other Versions
THDataVC<- subset(THData, Version != 1)
THDataVC<- subset(THDataVC, Version != 2)
THDataVC<- subset(THDataVC, Version != 4)
THDataVC<- subset(THDataVC, Version != 5)
THDataVC<- subset(THDataVC, Version != 6)
THDataVC<- subset(THDataVC, Version != 7)
THDataVC<- subset(THDataVC, Version != 8)
THDataVC<- subset(THDataVC, Version != 9)
table(THDataVC$Version)

#Get Pre and Post Values
###Approval Pre Message Summary
table(THDataVC$THPreTot)
length(THDataVC$THPreTot)
propPreVC<- table(THDataVC$THPreTot)/sum(table(THDataVC$THPreTot))
perPreVC <- propPreVC*100
perPreVC
## Remove 6 From Trophy Hunting Pre
THDataVC$THPreTot[THDataVC$THPreTot == 6] <- 3
THDataVC$THPreTot <- as.numeric(THDataVC$THPreTot)
table(THDataVC$THPreTot)
mean(THDataVC$THPreTot, na.rm = TRUE)
sd(THDataVC$THPreTot, na.rm = TRUE)
median(THDataVC$THPreTot, na.rm = TRUE)

###Approval Post Message Summary
table(THDataVC$THPostTot)
length(THDataVC$THPostTot)
propPostVC<- table(THDataVC$THPostTot)/sum(table(THDataVC$THPostTot))
perPostVC <- propPostVC*100
perPostVC
## Remove 6 From Trophy Hunting Post
THDataVC$THPostTot[THDataVC$THPostTot == 6] <- 3
THDataVC$THPostTot <- as.numeric(THDataVC$THPostTot)
table(THDataVC$THPostTot)
mean(THDataVC$THPostTot, na.rm = TRUE)
sd(THDataVC$THPostTot, na.rm = TRUE)
median(THDataVC$THPostTot, na.rm = TRUE)
```

```
###Difference
t.test(THDataVC$THPreTot, THDataVC$THPostTot, paired = TRUE)
##Effect Size
library(effsize)
cohen.d(THDataVC$THPreTot, THDataVC$THPostTot, paired = TRUE, within = FALSE)

###Test Difference Using Wilcoxon Signed Rank Test
wilcox.test(THDataVC$THPreTot, THDataVC$THPostTot, paired = TRUE)

###Effect Size Rank Biserial Correlation
library(effectsize)
rank_biserial(THDataVC$THPreTot, THDataVC$THPostTot, paired = TRUE, ci = 0.95)

##Message 4: Control Wildlife
#Remove Other Versions
THDataCW<- subset(THData, Version != 1)
THDataCW<- subset(THDataCW, Version != 2)
THDataCW<- subset(THDataCW, Version != 3)
THDataCW<- subset(THDataCW, Version != 5)
THDataCW<- subset(THDataCW, Version != 6)
THDataCW<- subset(THDataCW, Version != 7)
THDataCW<- subset(THDataCW, Version != 8)
THDataCW<- subset(THDataCW, Version != 9)
table(THDataCW$Version)

#Get Pre and Post Values
###Approval Pre Message Summary
table(THDataCW$THPreTot)
length(THDataCW$THPreTot)
propPreCW<- table(THDataCW$THPreTot)/sum(table(THDataCW$THPreTot))
perPreCW <- propPreCW*100
perPreCW
## Remove 6 From Trophy Hunting Pre
THDataCW$THPreTot[THDataCW$THPreTot == 6] <- 3
THDataCW$THPreTot <- as.numeric(THDataCW$THPreTot)
table(THDataCW$THPreTot)
mean(THDataCW$THPreTot, na.rm = TRUE)
sd(THDataCW$THPreTot, na.rm = TRUE)
median(THDataCW$THPreTot, na.rm = TRUE)

###Approval Post Message Summary
table(THDataCW$THPostTot)
length(THDataCW$THPostTot)
propPostCW<- table(THDataCW$THPostTot)/sum(table(THDataCW$THPostTot))
perPostCW <- propPostCW*100
perPostCW
## Remove 6 From Trophy Hunting Post
THDataCW$THPostTot[THDataCW$THPostTot == 6] <- 3
THDataCW$THPostTot <- as.numeric(THDataCW$THPostTot)
table(THDataCW$THPostTot)
mean(THDataCW$THPostTot, na.rm = TRUE)
sd(THDataCW$THPostTot, na.rm = TRUE)
median(THDataCW$THPostTot, na.rm = TRUE)

###Difference
t.test(THDataCW$THPreTot, THDataCW$THPostTot, paired = TRUE)
##Effect Size
library(effsize)
cohen.d(THDataCW$THPreTot, THDataCW$THPostTot, paired = TRUE, within = FALSE)

###Test Difference Using Wilcoxon Signed Rank Test
wilcox.test(THDataCW$THPreTot, THDataCW$THPostTot, paired = TRUE)

###Effect Size Rank Biserial Correlation
```

```
library(effectsize)
rank_biserial(THDataCW$THPreTot, THDataCW$THPostTot, paired = TRUE, ci = 0.95)

##Message 5: Social Identity Wildlife
#Remove Other Versions
THDataSW<- subset(THData, Version != 1)
THDataSW<- subset(THDataSW, Version != 2)
THDataSW<- subset(THDataSW, Version != 3)
THDataSW<- subset(THDataSW, Version != 4)
THDataSW<- subset(THDataSW, Version != 6)
THDataSW<- subset(THDataSW, Version != 7)
THDataSW<- subset(THDataSW, Version != 8)
THDataSW<- subset(THDataSW, Version != 9)
table(THDataSW$Version)

#Get Pre and Post Values
###Approval Pre Message Summary
table(THDataSW$THPreTot)
length(THDataSW$THPreTot)
propPreSW<- table(THDataSW$THPreTot)/sum(table(THDataSW$THPreTot))
perPreSW <- propPreSW*100
perPreSW
## Remove 6 From Trophy Hunting Pre
THDataSW$THPreTot[THDataSW$THPreTot == 6] <- 3
THDataSW$THPreTot <- as.numeric(THDataSW$THPreTot)
table(THDataSW$THPreTot)
mean(THDataSW$THPreTot, na.rm = TRUE)
sd(THDataSW$THPreTot, na.rm = TRUE)
median(THDataSW$THPreTot, na.rm = TRUE)

###Approval Post Message Summary
table(THDataSW$THPostTot)
length(THDataSW$THPostTot)
propPostSW<- table(THDataSW$THPostTot)/sum(table(THDataSW$THPostTot))
perPostSW <- propPostSW*100
perPostSW
## Remove 6 From Trophy Hunting Post
THDataSW$THPostTot[THDataSW$THPostTot == 6] <- 3
THDataSW$THPostTot <- as.numeric(THDataSW$THPostTot)
table(THDataSW$THPostTot)
mean(THDataSW$THPostTot, na.rm = TRUE)
sd(THDataSW$THPostTot, na.rm = TRUE)
median(THDataSW$THPostTot, na.rm = TRUE)

###Difference
t.test(THDataSW$THPreTot, THDataSW$THPostTot, paired = TRUE)
##Effect Size
library(effsize)
cohen.d(THDataSW$THPreTot, THDataSW$THPostTot, paired = TRUE, within = FALSE)

###Test Difference Using Wilcoxon Signed Rank Test
wilcox.test(THDataSW$THPreTot, THDataSW$THPostTot, paired = TRUE)

###Effect Size Rank Biserial Correlation
library(effectsize)
rank_biserial(THDataSW$THPreTot, THDataSW$THPostTot, paired = TRUE, ci = 0.95)

##Message 6: Value Wildlife
#Remove Other Versions
THDataVW<- subset(THData, Version != 1)
THDataVW<- subset(THDataVW, Version != 2)
THDataVW<- subset(THDataVW, Version != 3)
THDataVW<- subset(THDataVW, Version != 4)
THDataVW<- subset(THDataVW, Version != 5)
THDataVW<- subset(THDataVW, Version != 7)
```

```
THDataVW<- subset(THDataVW, Version != 8)
THDataVW<- subset(THDataVW, Version != 9)
table(THDataVW$Version)

#Get Pre and Post Values
###Approval Pre Message Summary
table(THDataVW$THPreTot)
length(THDataVW$THPreTot)
propPreVW<- table(THDataVW$THPreTot)/sum(table(THDataVW$THPreTot))
perPreVW <- propPreVW*100
perPreVW
## Remove 6 From Trophy Hunting Pre
THDataVW$THPreTot[THDataVW$THPreTot == 6] <- 3
THDataVW$THPreTot <- as.numeric(THDataVW$THPreTot)
table(THDataVW$THPreTot)
mean(THDataVW$THPreTot, na.rm = TRUE)
sd(THDataVW$THPreTot, na.rm = TRUE)
median(THDataVW$THPreTot, na.rm = TRUE)

###Approval Post Message Summary
table(THDataVW$THPostTot)
length(THDataVW$THPostTot)
propPostVW<- table(THDataVW$THPostTot)/sum(table(THDataVW$THPostTot))
perPostVW <- propPostVW*100
perPostVW
## Remove 6 From Trophy Hunting Post
THDataVW$THPostTot[THDataVW$THPostTot == 6] <- 3
THDataVW$THPostTot <- as.numeric(THDataVW$THPostTot)
table(THDataVW$THPostTot)
mean(THDataVW$THPostTot, na.rm = TRUE)
sd(THDataVW$THPostTot, na.rm = TRUE)
median(THDataVW$THPostTot, na.rm = TRUE)

###Difference
t.test(THDataVW$THPreTot, THDataVW$THPostTot, paired = TRUE)
##Effect Size
library(effsize)
cohen.d(THDataVW$THPreTot, THDataVW$THPostTot, paired = TRUE, within = FALSE)

###Test Difference Using Wilcoxon Signed Rank Test
wilcox.test(THDataVW$THPreTot, THDataVW$THPostTot, paired = TRUE)

###Effect Size Rank Biserial Correlation
library(effectsize)
rank_biserial(THDataVW$THPreTot, THDataVW$THPostTot, paired = TRUE, ci = 0.95)

##Message 7: Control Socioeconomic
#Remove Other Versions
THDataCS<- subset(THData, Version != 1)
THDataCS<- subset(THDataCS, Version != 2)
THDataCS<- subset(THDataCS, Version != 3)
THDataCS<- subset(THDataCS, Version != 4)
THDataCS<- subset(THDataCS, Version != 5)
THDataCS<- subset(THDataCS, Version != 6)
THDataCS<- subset(THDataCS, Version != 8)
THDataCS<- subset(THDataCS, Version != 9)
table(THDataCS$Version)

#Get Pre and Post Values
###Approval Pre Message Summary
table(THDataCS$THPreTot)
length(THDataCS$THPreTot)
propPreCS<- table(THDataCS$THPreTot)/sum(table(THDataCS$THPreTot))
perPreCS <- propPreCS*100
perPreCS
```

```
## Remove 6 From Trophy Hunting Pre
THDataCS$THPreTot[THDataCS$THPreTot == 6] <- 3
THDataCS$THPreTot <- as.numeric(THDataCS$THPreTot)
table(THDataCS$THPreTot)
mean(THDataCS$THPreTot, na.rm = TRUE)
sd(THDataCS$THPreTot, na.rm = TRUE)
median(THDataCS$THPreTot, na.rm = TRUE)

###Approval Post Message Summary
table(THDataCS$THPostTot)
length(THDataCS$THPostTot)
propPostCS<- table(THDataCS$THPostTot)/sum(table(THDataCS$THPostTot))
perPostCS <- propPostCS*100
perPostCS
## Remove 6 From Trophy Hunting Post
THDataCS$THPostTot[THDataCS$THPostTot == 6] <- 3
THDataCS$THPostTot <- as.numeric(THDataCS$THPostTot)
table(THDataCS$THPostTot)
mean(THDataCS$THPostTot, na.rm = TRUE)
sd(THDataCS$THPostTot, na.rm = TRUE)
median(THDataCS$THPostTot, na.rm = TRUE)

###Difference
t.test(THDataCS$THPreTot, THDataCS$THPostTot, paired = TRUE)
##Effect Size
library(effsize)
cohen.d(THDataCS$THPreTot, THDataCS$THPostTot, paired = TRUE, within = FALSE)

###Test Difference Using Wilcoxon Signed Rank Test
wilcox.test(THDataCS$THPreTot, THDataCS$THPostTot, paired = TRUE)

###Effect Size Rank Biserial Correlation
library(effectsize)
rank_biserial(THDataCS$THPreTot, THDataCS$THPostTot, paired = TRUE, ci = 0.95)

##Message 8: Social Identity Socioeconomic
#Remove Other Versions
THDataSS<- subset(THData, Version != 1)
THDataSS<- subset(THDataSS, Version != 2)
THDataSS<- subset(THDataSS, Version != 3)
THDataSS<- subset(THDataSS, Version != 4)
THDataSS<- subset(THDataSS, Version != 5)
THDataSS<- subset(THDataSS, Version != 6)
THDataSS<- subset(THDataSS, Version != 7)
THDataSS<- subset(THDataSS, Version != 9)
table(THDataSS$Version)

#Get Pre and Post Values
###Approval Pre Message Summary
table(THDataSS$THPreTot)
length(THDataSS$THPreTot)
propPreSS<- table(THDataSS$THPreTot)/sum(table(THDataSS$THPreTot))
perPreSS <- propPreSS*100
perPreSS
## Remove 6 From Trophy Hunting Pre
THDataSS$THPreTot[THDataSS$THPreTot == 6] <- 3
THDataSS$THPreTot <- as.numeric(THDataSS$THPreTot)
table(THDataSS$THPreTot)
mean(THDataSS$THPreTot, na.rm = TRUE)
sd(THDataSS$THPreTot, na.rm = TRUE)
median(THDataSS$THPreTot, na.rm = TRUE)

###Approval Post Message Summary
table(THDataSS$THPostTot)
length(THDataSS$THPostTot)
```

```
propPostSS<- table(THDataSS$THPostTot)/sum(table(THDataSS$THPostTot))
perPostSS <- propPostSS*100
perPostSS
## Remove 6 From Trophy Hunting Post
THDataSS$THPostTot[THDataSS$THPostTot == 6] <- 3
THDataSS$THPostTot <- as.numeric(THDataSS$THPostTot)
table(THDataSS$THPostTot)
mean(THDataSS$THPostTot, na.rm = TRUE)
sd(THDataSS$THPostTot, na.rm = TRUE)
median(THDataSS$THPostTot, na.rm = TRUE)

###Difference
t.test(THDataSS$THPreTot, THDataSS$THPostTot, paired = TRUE)
##Effect Size
library(effsize)
cohen.d(THDataSS$THPreTot, THDataSS$THPostTot, paired = TRUE, within = FALSE)

###Test Difference Using Wilcoxon Signed Rank Test
wilcox.test(THDataSS$THPreTot, THDataSS$THPostTot, paired = TRUE)

###Effect Size Rank Biserial Correlation
library(effectsize)
rank_biserial(THDataSS$THPreTot, THDataSS$THPostTot, paired = TRUE, ci = 0.95)

##Message 9: Value Socioeconomic
#Remove Other Versions
THDataVS<- subset(THData, Version != 1)
THDataVS<- subset(THDataVS, Version != 2)
THDataVS<- subset(THDataVS, Version != 3)
THDataVS<- subset(THDataVS, Version != 4)
THDataVS<- subset(THDataVS, Version != 5)
THDataVS<- subset(THDataVS, Version != 6)
THDataVS<- subset(THDataVS, Version != 7)
THDataVS<- subset(THDataVS, Version != 8)
table(THDataVS$Version)

#Get Pre and Post Values

###Approval Pre Message Summary
table(THDataVS$THPreTot)
length(THDataVS$THPreTot)
propPreVS<- table(THDataVS$THPreTot)/sum(table(THDataVS$THPreTot))
perPreVS <- propPreVS*100
perPreVS
## Remove 6 From Trophy Hunting Pre
THDataVS$THPreTot[THDataVS$THPreTot == 6] <- 3
THDataVS$THPreTot <- as.numeric(THDataVS$THPreTot)
table(THDataVS$THPreTot)
mean(THDataVS$THPreTot, na.rm = TRUE)
sd(THDataVS$THPreTot, na.rm = TRUE)
median(THDataVS$THPreTot, na.rm = TRUE)

###Approval Post Message Summary
table(THDataVS$THPostTot)
length(THDataVS$THPostTot)
propPostVS<- table(THDataVS$THPostTot)/sum(table(THDataVS$THPostTot))
perPostVS <- propPostVS*100
perPostVS
## Remove 6 From Trophy Hunting Post
THDataVS$THPostTot[THDataVS$THPostTot == 6] <- 3
THDataVS$THPostTot <- as.numeric(THDataVS$THPostTot)
table(THDataVS$THPostTot)
mean(THDataVS$THPostTot, na.rm = TRUE)
sd(THDataVS$THPostTot, na.rm = TRUE)
median(THDataVS$THPostTot, na.rm = TRUE)
```

```
###Difference
t.test(THDataVS$THPreTot, THDataVS$THPostTot, paired = TRUE)
##Effect Size
library(effsize)
cohen.d(THDataVS$THPreTot, THDataVS$THPostTot, paired = TRUE, within = FALSE)

###Test Difference Using Wilcoxon Signed Rank Test
wilcox.test(THDataVS$THPreTot, THDataVS$THPostTot, paired = TRUE)

###Effect Size Rank Biserial Correlation
library(effectsize)
rank_biserial(THDataVS$THPreTot, THDataVS$THPostTot, paired = TRUE, ci = 0.95)

######Analysis of TH Attitudes Using Political Identity and Personal Values
######Political Alignment

####Summary of Political Alignment
Politics <- THData$PoliticsTot
Politics
table(Politics)
length(Politics)
propPolitics<- table(Politics)/sum(table(Politics))
perPolitics <- propPolitics*100
perPolitics

###Differences in TH Approval by Politics (Use PRE to avoid confounding effect)
###Approval Pre Summary
table(THData$THPreTot)
length(THData$THPreTot)
THData$THPreTot <- as.numeric(THData$THPreTot)
#Get TH Approval by Politics
table(THData$THPreTot, THData$PoliticsTot)

## Remove 6 From Trophy Hunting Pre
THData$THPreTot[THData$THPreTot == 6] <- 3
table(THData$THPreTot)
length(THData$THPreTot)
#Get TH Approval by Politics
table(THData$THPreTot, THData$PoliticsTot)
aggregate(THPreTot ~ PoliticsTot, data = THData, FUN = mean)
aggregate(THPreTot ~ PoliticsTot, data = THData, FUN = sd)
aggregate(THPreTot ~ PoliticsTot, data = THData, FUN = median)
mean(THData$THPreTot, na.rm = TRUE)
sd(THData$THPreTot, na.rm = TRUE)
median(THData$THPreTot, na.rm = TRUE)

##Determine Differences by Politics Group
##ANOVA Model
PolMod <- aov(THPreTot ~ PoliticsTot, data = THData)
summary(PolMod)

library(effectsize)
eta_squared(PolMod, partial = FALSE)

##Bonferroni
pairwise.t.test(THData$THPreTot, THData$PoliticsTot, p.adj = "bonf")

##Make table with Bonf significance
PolApp <- aggregate(THPreTot ~ PoliticsTot, data = THData, FUN = mean)
PolApp
#define new column
BonSigPol <- c('a','ab','b', 'c', 'c')
#add column
PolSig <- cbind(PolApp, BonSigPol)
```

```
PolSig

## Check normality of the residuals with the Shapiro-Wilk test
shapiro.test(PolMod$residuals)
###Check for equality of variance
library(car)
leveneTest(THPreTot ~PoliticsTot,
           data = THData)

##Kruskal Wallis Test
library(rstatix)
kruskal.test(THPreTot ~ PoliticsTot, data = THData)

# pairwise using dunn test
dunn_test(THPreTot ~ PoliticsTot, data = THData, p.adjust = "bonferroni")

##Make table with Bonf significance
NPPolApp <- aggregate(THPreTot ~ PoliticsTot, data = THData, FUN = median)
NPPolApp
#define new column to add
NPBonSigPol <- c('a','ab','b', 'c', 'c')
#add column called 'new'
NPPolSig <- cbind(NPPolApp, NPBonSigPol)
#view new data frame
NPPolSig

####Values Summary

##Family and Friendship
THData$ValueFamilyTot <- as.numeric(THData$ValueFamilyTot)
Fam <- THData$ValueFamilyTot
Fam
table(Fam)
length(Fam)
propFam<- table(Fam)/sum(table(Fam))
perFam <- propFam*100
perFam
mean(Fam, na.rm = TRUE)
sd(Fam, na.rm = TRUE)

##Conservation and Stewardship
THData$ValueConservationTot <- as.numeric(THData$ValueConservationTot)
Constw <- THData$ValueConservationTot
Constw
table(Constw)
length(Constw)
propConst<- table(Constw)/sum(table(Constw))
perConst <- propConst*100
perConst
mean(Constw, na.rm = TRUE)
sd(Constw, na.rm = TRUE)

##Economic and Financial
THData$ValueEconomicTot <- as.numeric(THData$ValueEconomicTot)
Ecofin <- THData$ValueEconomicTot
Ecofin
table(Ecofin)
length(Ecofin)
propEco<- table(Ecofin)/sum(table(Ecofin))
perEco <- propEco*100
perEco
mean(Ecofin, na.rm = TRUE)
sd(Ecofin, na.rm = TRUE)

##Growth and Learning
```

```
THData$ValueGrowthTot <- as.numeric(THData$ValueGrowthTot)
Growth <- THData$ValueGrowthTot
Growth
table(Growth)
length(Growth)
propGro<- table(Growth)/sum(table(Growth))
perGro <- propGro*100
perGro
mean(Growth, na.rm = TRUE)
sd(Growth, na.rm = TRUE)

##Recreation and Fun
THData$ValueFunTot <- as.numeric(THData$ValueFunTot)
Recfun <- THData$ValueFunTot
Recfun
table(Recfun)
length(Recfun)
propFun<- table(Recfun)/sum(table(Recfun))
perFun <- propFun*100
perFun
mean(Recfun, na.rm = TRUE)
sd(Recfun, na.rm = TRUE)

##############Analysis of Values Effect on TH
###Approval Pre Summary
table(THData$THPreTot)
length(THData$THPreTot)
THData$THPreTot <- as.numeric(THData$THPreTot)
## Remove 6 From Trophy Hunting Pre
THData$THPreTot[THData$THPreTot == 6] <- 3
table(THData$THPreTot)
length(THData$THPreTot)

###Family/Friendship
#Get TH Approval by Family Rank
table(THData$THPreTot, THData$ValueFamilyTot)
aggregate(THPreTot ~ ValueFamilyTot, data = THData, FUN = mean)
aggregate(THPreTot ~ ValueFamilyTot, data = THData, FUN = sd)
aggregate(THPreTot ~ ValueFamilyTot, data = THData, FUN = median)

##Determine Differences by Family Rank
##ANOVA Model
FamMod <- aov(THPreTot ~ ValueFamilyTot, data = THData)
summary(FamMod)

library(effectsize)
eta_squared(FamMod, partial = FALSE)

## Check normality of the residuals with the Shapiro-Wilk test
shapiro.test(FamMod$residuals)
###Check for equality of variance
library(car)
leveneTest(THPreTot ~ValueFamilyTot,
           data = THData)

##Kruskal Wallis Test
library(rstatix)
kruskal.test(THPreTot ~ ValueFamilyTot, data = THData)

###Conservation/Stewardship
#Get TH Approval by Conservation Rank
table(THData$THPreTot, THData$ValueConservationTot)
aggregate(THPreTot ~ ValueConservationTot, data = THData, FUN = mean)
aggregate(THPreTot ~ ValueConservationTot, data = THData, FUN = sd)
aggregate(THPreTot ~ ValueConservationTot, data = THData, FUN = median)
```

```
##Determine Differences by Conservation Rank
##ANOVA Model
ConsMod <- aov(THPreTot ~ ValueConservationTot, data = THData)
summary(ConsMod)

library(effectsize)
eta_squared(ConsMod, partial = FALSE)

##Bonferroni
pairwise.t.test(THData$THPreTot, THData$ValueConservationTot, p.adj = "bonf")

##Make table with Bonf significance
ConsVApp <- aggregate(THPreTot ~ ValueConservationTot, data = THData,
                      FUN = mean)
ConsVApp
#define new column
BonSigConsV <- c('a','a','a', 'ab', 'b')
#add column
ConsVSig <- cbind(ConsVApp, BonSigConsV)
ConsVSig

## Check normality of the residuals with the Shapiro-Wilk test
shapiro.test(ConsMod$residuals)
###Check for equality of variance
library(car)
leveneTest(THPreTot ~ValueConservationTot,
           data = THData)

##Kruskal Wallis Test
library(rstatix)
kruskal.test(THPreTot ~ ValueConservationTot, data = THData)

# pairwise using dunn test
dunn_test(THPreTot ~ ValueConservationTot, data = THData,
          p.adjust = "bonferroni")

##Make table with Bonf significance
NPConsVApp <- aggregate(THPreTot ~ ValueConservationTot, data = THData,
                        FUN = median)
NPConsVApp
#define new column to add
NPBonSigConsV <- c('a','a','a', 'ab', 'b')
#add column called 'new'
NPConsVSig <- cbind(NPConsVApp, NPBonSigConsV)
#view new data frame
NPConsVSig

###Economic/Financial Success
#Get TH Approval by Economic Rank
table(THData$THPreTot, THData$ValueEconomicTot)
aggregate(THPreTot ~ ValueEconomicTot, data = THData, FUN = mean)
aggregate(THPreTot ~ ValueEconomicTot, data = THData, FUN = sd)
aggregate(THPreTot ~ ValueEconomicTot, data = THData, FUN = median)

##Determine Differences by Economic Rank
##ANOVA Model
EcoVMod <- aov(THPreTot ~ ValueEconomicTot, data = THData)
summary(EcoVMod)

library(effectsize)
eta_squared(EcoVMod, partial = FALSE)

## Check normality of the residuals with the Shapiro-Wilk test
shapiro.test(EcoVMod$residuals)
```

```
###Check for equality of variance
library(car)
leveneTest(THPreTot ~ValueEconomicTot,
           data = THData)

##Kruskal Wallis Test
library(rstatix)
kruskal.test(THPreTot ~ ValueEconomicTot, data = THData)

# pairwise using dunn test
dunn_test(THPreTot ~ ValueEconomicTot, data = THData,
          p.adjust = "bonferroni")
###Bonferroni correction = ns result

###Growth and Learning
#Get TH Approval by Growth Rank
table(THData$THPreTot, THData$ValueGrowthTot)
aggregate(THPreTot ~ ValueGrowthTot, data = THData, FUN = mean)
aggregate(THPreTot ~ ValueGrowthTot, data = THData, FUN = sd)
aggregate(THPreTot ~ ValueGrowthTot, data = THData, FUN = median)

##Determine Differences by Growth Rank
##ANOVA Model
GroMod <- aov(THPreTot ~ ValueGrowthTot, data = THData)
summary(GroMod)

library(effectsize)
eta_squared(GroMod, partial = FALSE)

## Check normality of the residuals with the Shapiro-Wilk test
shapiro.test(GroMod$residuals)
###Check for equality of variance
library(car)
leveneTest(THPreTot ~ValueGrowthTot,
           data = THData)

##Kruskal Wallis Test
library(rstatix)
kruskal.test(THPreTot ~ ValueGrowthTot, data = THData)

###Recreation/Fun
#Get TH Approval by Recreation Rank
table(THData$THPreTot, THData$ValueFunTot)
aggregate(THPreTot ~ ValueFunTot, data = THData, FUN = mean)
aggregate(THPreTot ~ ValueFunTot, data = THData, FUN = sd)
aggregate(THPreTot ~ ValueFunTot, data = THData, FUN = median)

##Determine Differences by Recreation Rank
##ANOVA Model
RecMod <- aov(THPreTot ~ ValueFunTot, data = THData)
summary(RecMod)

library(effectsize)
eta_squared(RecMod, partial = FALSE)

##Bonferroni
pairwise.t.test(THData$THPreTot, THData$ValueFunTot, p.adj = "bonf")

##Make table with Bonf significance
RecApp <- aggregate(THPreTot ~ ValueFunTot, data = THData, FUN = mean)
RecApp
#define new column
BonSigRec <- c('a','ab','abc', 'bc', 'c')
#add column
RecSig <- cbind(RecApp, BonSigRec)
```

```
RecSig

## Check normality of the residuals with the Shapiro-Wilk test
shapiro.test(RecMod$residuals)
###Check for equality of variance
library(car)
leveneTest(THPreTot ~ValueFunTot,
           data = THData)

##Kruskal Wallis Test
library(rstatix)
kruskal.test(THPreTot ~ ValueFunTot, data = THData)

# pairwise using dunn test
dunn_test(THPreTot ~ ValueFunTot, data = THData,
          p.adjust = "bonferroni")

##Make table with Bonf significance
NPRecApp <- aggregate(THPreTot ~ ValueFunTot, data = THData, FUN = median)
NPRecApp
#define new column to add
NPBonSigRec <- c('a','ab','ab', 'bc', 'c')
#add column called 'new'
NPRecSig <- cbind(NPRecApp, NPBonSigRec)
#view new data frame
NPRecSig

#####Politics Detailed Analysis
###Determining effects of different messages on liberals and conservatives

###Create Conservative and Liberal Extremes
#Conservative
THData$PoliticsTot[THData$PoliticsTot == 2] <- 1
#Liberal
THData$PoliticsTot[THData$PoliticsTot == 4] <- 2
THData$PoliticsTot[THData$PoliticsTot == 5] <- 2
#Exclude Centrists
THData$PoliticsTot[THData$PoliticsTot == 3] <- NA
table(THData$PoliticsTot)

###Create Conservative Only Sample
THDataRight<- subset(THData, PoliticsTot != 2)
table(THDataRight$PoliticsTot)

########Difference in TH Attitudes Pre and Post Message
######Pre Message
###Approval Pre Message Summary
table(THDataRight$THPreTot)
length(THDataRight$THPreTot)
propPreAllR<- table(THDataRight$THPreTot)/sum(table(THDataRight$THPreTot))
perPreAllR <- propPreAllR*100
perPreAllR
THDataRight$THPreTot <- as.numeric(THDataRight$THPreTot)

#Get TH Approval by Message
table(THDataRight$THPreTot, THDataRight$Version)

## Remove 6 From Trophy Hunting Pre
THDataRight$THPreTot[THDataRight$THPreTot == 6] <- 3
table(THDataRight$THPreTot)
#Get TH Approval by Message
table(THDataRight$THPreTot, THDataRight$Version)
aggregate(THPreTot ~ Version, data = THDataRight, FUN = mean)
aggregate(THPreTot ~ Version, data = THDataRight, FUN = sd)
aggregate(THPreTot ~ Version, data = THDataRight, FUN = median)
```

```
mean(THDataRight$THPreTot, na.rm = TRUE)
sd(THDataRight$THPreTot, na.rm = TRUE)
median(THDataRight$THPreTot, na.rm = TRUE)

##Determine Differences by Message Group
##ANOVA Model
RightPreMesMod <- aov(THPreTot ~ Version, data = THDataRight)
summary(RightPreMesMod)

library(effectsize)
eta_squared(RightPreMesMod, partial = FALSE)

## Check normality of the residuals with the Shapiro-Wilk test
shapiro.test(RightPreMesMod$residuals)
###Check for equality of variance
library(car)
leveneTest(THPreTot ~Version,
           data = THDataRight)

##Kruskal Wallis Test
library(rstatix)
kruskal.test(THPreTot ~ Version, data = THDataRight)

#####Post Message
###Approval Post Message Summary
table(THDataRight$THPostTot)
length(THDataRight$THPostTot)
propPostAllR<- table(THDataRight$THPostTot)/sum(table(THDataRight$THPostTot))
perPostAllR <- propPostAllR*100
perPostAllR
THDataRight$THPostTot <- as.numeric(THDataRight$THPostTot)
#Get TH Approval by Message
table(THDataRight$THPostTot, THDataRight$Version)

## Remove 6 From Trophy Hunting Post
THDataRight$THPostTot[THDataRight$THPostTot == 6] <- 3
table(THDataRight$THPostTot)
#Get TH Approval by Message
table(THDataRight$THPostTot, THDataRight$Version)
aggregate(THPostTot ~ Version, data = THDataRight, FUN = mean)
aggregate(THPostTot ~ Version, data = THDataRight, FUN = sd)
aggregate(THPostTot ~ Version, data = THDataRight, FUN = median)
mean(THDataRight$THPostTot, na.rm = TRUE)
sd(THDataRight$THPostTot, na.rm = TRUE)
median(THDataRight$THPostTot, na.rm = TRUE)

##Determine Differences by Message Group
##ANOVA Model
RightPostMesMod <- aov(THPostTot ~ Version, data = THDataRight)
summary(RightPostMesMod)

library(effectsize)
eta_squared(RightPostMesMod, partial = FALSE)

##Bonferroni
pairwise.t.test(THDataRight$THPostTot, THDataRight$Version, p.adj = "bonf")

#Bonferroni results came up non-significant, note in paper

## Check normality of the residuals with the Shapiro-Wilk test
shapiro.test(RightPostMesMod$residuals)
###Check for equality of variance
library(car)
leveneTest(THPostTot ~Version,
           data = THDataRight)
```

```
##Kruskal Wallis Test
library(rstatix)
kruskal.test(THPostTot ~ Version, data = THDataRight)

# pairwise using dunn test
DunnRightPost <- dunn_test(THPostTot ~ Version, data = THDataRight,
                           p.adjust = "bonferroni")
DunnRightPost
print(DunnRightPost, n = 36)

##Post hoc tests not significant

##Separate by Message
table(THDataRight$Version)

##Message 1: Control Control
#Remove Other Versions
THDataRCC<- subset(THDataRight, Version != 2)
THDataRCC<- subset(THDataRCC, Version != 3)
THDataRCC<- subset(THDataRCC, Version != 4)
THDataRCC<- subset(THDataRCC, Version != 5)
THDataRCC<- subset(THDataRCC, Version != 6)
THDataRCC<- subset(THDataRCC, Version != 7)
THDataRCC<- subset(THDataRCC, Version != 8)
THDataRCC<- subset(THDataRCC, Version != 9)
table(THDataRCC$Version)

#Get Pre and Post Values
###Approval Pre Message Summary
table(THDataRCC$THPreTot)
length(THDataRCC$THPreTot)
propPreRCC<- table(THDataRCC$THPreTot)/sum(table(THDataRCC$THPreTot))
perPreRCC <- propPreRCC*100
perPreRCC
## Remove 6 From Trophy Hunting Pre
THDataRCC$THPreTot[THDataRCC$THPreTot == 6] <- 3
THDataRCC$THPreTot <- as.numeric(THDataRCC$THPreTot)
table(THDataRCC$THPreTot)
length(THDataRCC$THPreTot)
mean(THDataRCC$THPreTot, na.rm = TRUE)
sd(THDataRCC$THPreTot, na.rm = TRUE)
median(THDataRCC$THPreTot, na.rm = TRUE)

###Approval Post Message Summary
table(THDataRCC$THPostTot)
length(THDataRCC$THPostTot)
propPostRCC<- table(THDataRCC$THPostTot)/sum(table(THDataRCC$THPostTot))
perPostRCC <- propPostRCC*100
perPostRCC
## Remove 6 From Trophy Hunting Post
THDataRCC$THPostTot[THDataRCC$THPostTot == 6] <- 3
THDataRCC$THPostTot <- as.numeric(THDataRCC$THPostTot)
table(THDataRCC$THPostTot)
mean(THDataRCC$THPostTot, na.rm = TRUE)
sd(THDataRCC$THPostTot, na.rm = TRUE)
median(THDataRCC$THPostTot, na.rm = TRUE)

###Difference
t.test(THDataRCC$THPreTot, THDataRCC$THPostTot, paired = TRUE)
##Effect Size
library(effsize)
cohen.d(THDataRCC$THPreTot, THDataRCC$THPostTot, paired = TRUE, within = FALSE)

###Test Difference Using Wilcoxon Signed Rank Test
```

```
wilcox.test(THDataRCC$THPreTot, THDataRCC$THPostTot, paired = TRUE)

###Effect Size Rank Biserial Correlation
library(effectsize)
rank_biserial(THDataRCC$THPreTot, THDataRCC$THPostTot, paired = TRUE, ci = 0.95)

##Message 2: Social Identity Control
#Remove Other Versions
THDataRSC<- subset(THDataRight, Version != 1)
THDataRSC<- subset(THDataRSC, Version != 3)
THDataRSC<- subset(THDataRSC, Version != 4)
THDataRSC<- subset(THDataRSC, Version != 5)
THDataRSC<- subset(THDataRSC, Version != 6)
THDataRSC<- subset(THDataRSC, Version != 7)
THDataRSC<- subset(THDataRSC, Version != 8)
THDataRSC<- subset(THDataRSC, Version != 9)
table(THDataRSC$Version)

#Get Pre and Post Values
###Approval Pre Message Summary
table(THDataRSC$THPreTot)
length(THDataRSC$THPreTot)
propPreRSC<- table(THDataRSC$THPreTot)/sum(table(THDataRSC$THPreTot))
perPreRSC <- propPreRSC*100
perPreRSC
## Remove 6 From Trophy Hunting Pre
THDataRSC$THPreTot[THDataRSC$THPreTot == 6] <- 3
THDataRSC$THPreTot <- as.numeric(THDataRSC$THPreTot)
table(THDataRSC$THPreTot)
mean(THDataRSC$THPreTot, na.rm = TRUE)
sd(THDataRSC$THPreTot, na.rm = TRUE)
median(THDataRSC$THPreTot, na.rm = TRUE)

###Approval Post Message Summary
table(THDataRSC$THPostTot)
length(THDataRSC$THPostTot)
propPostRSC<- table(THDataRSC$THPostTot)/sum(table(THDataRSC$THPostTot))
perPostRSC <- propPostRSC*100
perPostRSC
## Remove 6 From Trophy Hunting Post
THDataRSC$THPostTot[THDataRSC$THPostTot == 6] <- 3
THDataRSC$THPostTot <- as.numeric(THDataRSC$THPostTot)
table(THDataRSC$THPostTot)
mean(THDataRSC$THPostTot, na.rm = TRUE)
sd(THDataRSC$THPostTot, na.rm = TRUE)
median(THDataRSC$THPostTot, na.rm = TRUE)

###Difference
t.test(THDataRSC$THPreTot, THDataRSC$THPostTot, paired = TRUE)
##Effect Size
library(effsize)
cohen.d(THDataRSC$THPreTot, THDataRSC$THPostTot, paired = TRUE, within = FALSE)

###Test Difference Using Wilcoxon Signed Rank Test
wilcox.test(THDataRSC$THPreTot, THDataRSC$THPostTot, paired = TRUE)

###Effect Size Rank Biserial Correlation
library(effectsize)
rank_biserial(THDataRSC$THPreTot, THDataRSC$THPostTot, paired = TRUE, ci = 0.95)

##Message 3: Value Control
#Remove Other Versions
THDataRVC<- subset(THDataRight, Version != 1)
THDataRVC<- subset(THDataRVC, Version != 2)
THDataRVC<- subset(THDataRVC, Version != 4)
```

```
THDataRVC<- subset(THDataRVC, Version != 5)
THDataRVC<- subset(THDataRVC, Version != 6)
THDataRVC<- subset(THDataRVC, Version != 7)
THDataRVC<- subset(THDataRVC, Version != 8)
THDataRVC<- subset(THDataRVC, Version != 9)
table(THDataRVC$Version)

#Get Pre and Post Values
###Approval Pre Message Summary
table(THDataRVC$THPreTot)
length(THDataRVC$THPreTot)
propPreRVC<- table(THDataRVC$THPreTot)/sum(table(THDataRVC$THPreTot))
perPreRVC <- propPreRVC*100
perPreRVC
## Remove 6 From Trophy Hunting Pre
THDataRVC$THPreTot[THDataRVC$THPreTot == 6] <- 3
THDataRVC$THPreTot <- as.numeric(THDataRVC$THPreTot)
table(THDataRVC$THPreTot)
mean(THDataRVC$THPreTot, na.rm = TRUE)
sd(THDataRVC$THPreTot, na.rm = TRUE)
median(THDataRVC$THPreTot, na.rm = TRUE)

###Approval Post Message Summary
table(THDataRVC$THPostTot)
length(THDataRVC$THPostTot)
propPostRVC<- table(THDataRVC$THPostTot)/sum(table(THDataRVC$THPostTot))
perPostRVC <- propPostRVC*100
perPostRVC
## Remove 6 From Trophy Hunting Post
THDataRVC$THPostTot[THDataRVC$THPostTot == 6] <- 3
THDataRVC$THPostTot <- as.numeric(THDataRVC$THPostTot)
table(THDataRVC$THPostTot)
mean(THDataRVC$THPostTot, na.rm = TRUE)
sd(THDataRVC$THPostTot, na.rm = TRUE)
median(THDataRVC$THPostTot, na.rm = TRUE)

###Difference
t.test(THDataRVC$THPreTot, THDataRVC$THPostTot, paired = TRUE)
##Effect Size
library(effsize)
cohen.d(THDataRVC$THPreTot, THDataRVC$THPostTot, paired = TRUE, within = FALSE)

###Test Difference Using Wilcoxon Signed Rank Test
wilcox.test(THDataRVC$THPreTot, THDataRVC$THPostTot, paired = TRUE)

###Effect Size Rank Biserial Correlation
library(effectsize)
rank_biserial(THDataRVC$THPreTot, THDataRVC$THPostTot, paired = TRUE, ci = 0.95)

##Message 4: Control Wildlife
#Remove Other Versions
THDataRCW<- subset(THDataRight, Version != 1)
THDataRCW<- subset(THDataRCW, Version != 2)
THDataRCW<- subset(THDataRCW, Version != 3)
THDataRCW<- subset(THDataRCW, Version != 5)
THDataRCW<- subset(THDataRCW, Version != 6)
THDataRCW<- subset(THDataRCW, Version != 7)
THDataRCW<- subset(THDataRCW, Version != 8)
THDataRCW<- subset(THDataRCW, Version != 9)
table(THDataRCW$Version)

#Get Pre and Post Values
###Approval Pre Message Summary
table(THDataRCW$THPreTot)
length(THDataRCW$THPreTot)
```

```
propPreRCW<- table(THDataRCW$THPreTot)/sum(table(THDataRCW$THPreTot))
perPreRCW <- propPreRCW*100
perPreRCW
## Remove 6 From Trophy Hunting Pre
THDataRCW$THPreTot[THDataRCW$THPreTot == 6] <- 3
THDataRCW$THPreTot <- as.numeric(THDataRCW$THPreTot)
table(THDataRCW$THPreTot)
mean(THDataRCW$THPreTot, na.rm = TRUE)
sd(THDataRCW$THPreTot, na.rm = TRUE)
median(THDataRCW$THPreTot, na.rm = TRUE)

###Approval Post Message Summary
table(THDataRCW$THPostTot)
length(THDataRCW$THPostTot)
propPostRCW<- table(THDataRCW$THPostTot)/sum(table(THDataRCW$THPostTot))
perPostRCW <- propPostRCW*100
perPostRCW
## Remove 6 From Trophy Hunting Post
THDataRCW$THPostTot[THDataRCW$THPostTot == 6] <- 3
THDataRCW$THPostTot <- as.numeric(THDataRCW$THPostTot)
table(THDataRCW$THPostTot)
mean(THDataRCW$THPostTot, na.rm = TRUE)
sd(THDataRCW$THPostTot, na.rm = TRUE)
median(THDataRCW$THPostTot, na.rm = TRUE)

###Difference
t.test(THDataRCW$THPreTot, THDataRCW$THPostTot, paired = TRUE)
##Effect Size
library(effsize)
cohen.d(THDataRCW$THPreTot, THDataRCW$THPostTot, paired = TRUE, within = FALSE)

###Test Difference Using Wilcoxon Signed Rank Test
wilcox.test(THDataRCW$THPreTot, THDataRCW$THPostTot, paired = TRUE)

###Effect Size Rank Biserial Correlation
library(effectsize)
rank_biserial(THDataRCW$THPreTot, THDataRCW$THPostTot, paired = TRUE, ci = 0.95)

##Message 5: Social Identity Wildlife
#Remove Other Versions
THDataRSW<- subset(THDataRight, Version != 1)
THDataRSW<- subset(THDataRSW, Version != 2)
THDataRSW<- subset(THDataRSW, Version != 3)
THDataRSW<- subset(THDataRSW, Version != 4)
THDataRSW<- subset(THDataRSW, Version != 6)
THDataRSW<- subset(THDataRSW, Version != 7)
THDataRSW<- subset(THDataRSW, Version != 8)
THDataRSW<- subset(THDataRSW, Version != 9)
table(THDataRSW$Version)

#Get Pre and Post Values
###Approval Pre Message Summary
table(THDataRSW$THPreTot)
length(THDataRSW$THPreTot)
propPreRSW<- table(THDataRSW$THPreTot)/sum(table(THDataRSW$THPreTot))
perPreRSW <- propPreRSW*100
perPreRSW
## Remove 6 From Trophy Hunting Pre
THDataRSW$THPreTot[THDataRSW$THPreTot == 6] <- 3
THDataRSW$THPreTot <- as.numeric(THDataRSW$THPreTot)
table(THDataRSW$THPreTot)
mean(THDataRSW$THPreTot, na.rm = TRUE)
sd(THDataRSW$THPreTot, na.rm = TRUE)
median(THDataRSW$THPreTot, na.rm = TRUE)
```

```
###Approval Post Message Summary
table(THDataRSW$THPostTot)
length(THDataRSW$THPostTot)
propPostRSW<- table(THDataRSW$THPostTot)/sum(table(THDataRSW$THPostTot))
perPostRSW <- propPostRSW*100
perPostRSW
## Remove 6 From Trophy Hunting Post
THDataRSW$THPostTot[THDataRSW$THPostTot == 6] <- 3
THDataRSW$THPostTot <- as.numeric(THDataRSW$THPostTot)
table(THDataRSW$THPostTot)
mean(THDataRSW$THPostTot, na.rm = TRUE)
sd(THDataRSW$THPostTot, na.rm = TRUE)
median(THDataRSW$THPostTot, na.rm = TRUE)

###Difference
t.test(THDataRSW$THPreTot, THDataRSW$THPostTot, paired = TRUE)
##Effect Size
library(effsize)
cohen.d(THDataRSW$THPreTot, THDataRSW$THPostTot, paired = TRUE, within = FALSE)

###Test Difference Using Wilcoxon Signed Rank Test
wilcox.test(THDataRSW$THPreTot, THDataRSW$THPostTot, paired = TRUE)

###Effect Size Rank Biserial Correlation
library(effectsize)
rank_biserial(THDataRSW$THPreTot, THDataRSW$THPostTot, paired = TRUE, ci = 0.95)

##Message 6: Value Wildlife
#Remove Other Versions
THDataRVW<- subset(THDataRight, Version != 1)
THDataRVW<- subset(THDataRVW, Version != 2)
THDataRVW<- subset(THDataRVW, Version != 3)
THDataRVW<- subset(THDataRVW, Version != 4)
THDataRVW<- subset(THDataRVW, Version != 5)
THDataRVW<- subset(THDataRVW, Version != 7)
THDataRVW<- subset(THDataRVW, Version != 8)
THDataRVW<- subset(THDataRVW, Version != 9)
table(THDataRVW$Version)

#Get Pre and Post Values
###Approval Pre Message Summary
table(THDataRVW$THPreTot)
length(THDataRVW$THPreTot)
propPreRVW<- table(THDataRVW$THPreTot)/sum(table(THDataRVW$THPreTot))
perPreRVW <- propPreRVW*100
perPreRVW
## Remove 6 From Trophy Hunting Pre
THDataRVW$THPreTot[THDataRVW$THPreTot == 6] <- 3
THDataRVW$THPreTot <- as.numeric(THDataRVW$THPreTot)
table(THDataRVW$THPreTot)
mean(THDataRVW$THPreTot, na.rm = TRUE)
sd(THDataRVW$THPreTot, na.rm = TRUE)
median(THDataRVW$THPreTot, na.rm = TRUE)

###Approval Post Message Summary
table(THDataRVW$THPostTot)
length(THDataRVW$THPostTot)
propPostRVW<- table(THDataRVW$THPostTot)/sum(table(THDataRVW$THPostTot))
perPostRVW <- propPostRVW*100
perPostRVW
## Remove 6 From Trophy Hunting Post
THDataRVW$THPostTot[THDataRVW$THPostTot == 6] <- 3
THDataRVW$THPostTot <- as.numeric(THDataRVW$THPostTot)
table(THDataRVW$THPostTot)
mean(THDataRVW$THPostTot, na.rm = TRUE)
```

```
sd(THDataRVW$THPostTot, na.rm = TRUE)
median(THDataRVW$THPostTot, na.rm = TRUE)

###Difference
t.test(THDataRVW$THPreTot, THDataRVW$THPostTot, paired = TRUE)
##Effect Size
library(effsize)
cohen.d(THDataRVW$THPreTot, THDataRVW$THPostTot, paired = TRUE, within = FALSE)

###Test Difference Using Wilcoxon Signed Rank Test
wilcox.test(THDataRVW$THPreTot, THDataRVW$THPostTot, paired = TRUE)

###Effect Size Rank Biserial Correlation
library(effectsize)
rank_biserial(THDataRVW$THPreTot, THDataRVW$THPostTot, paired = TRUE, ci = 0.95)

##Message 7: Control Socioeconomic
#Remove Other Versions
THDataRCS<- subset(THDataRight, Version != 1)
THDataRCS<- subset(THDataRCS, Version != 2)
THDataRCS<- subset(THDataRCS, Version != 3)
THDataRCS<- subset(THDataRCS, Version != 4)
THDataRCS<- subset(THDataRCS, Version != 5)
THDataRCS<- subset(THDataRCS, Version != 6)
THDataRCS<- subset(THDataRCS, Version != 8)
THDataRCS<- subset(THDataRCS, Version != 9)
table(THDataRCS$Version)

#Get Pre and Post Values
###Approval Pre Message Summary
table(THDataRCS$THPreTot)
length(THDataRCS$THPreTot)
propPreRCS<- table(THDataRCS$THPreTot)/sum(table(THDataRCS$THPreTot))
perPreRCS <- propPreRCS*100
perPreRCS
## Remove 6 From Trophy Hunting Pre
THDataRCS$THPreTot[THDataRCS$THPreTot == 6] <- 3
THDataRCS$THPreTot <- as.numeric(THDataRCS$THPreTot)
table(THDataRCS$THPreTot)
mean(THDataRCS$THPreTot, na.rm = TRUE)
sd(THDataRCS$THPreTot, na.rm = TRUE)
median(THDataRCS$THPreTot, na.rm = TRUE)

###Approval Post Message Summary
table(THDataRCS$THPostTot)
length(THDataRCS$THPostTot)
propPostRCS<- table(THDataRCS$THPostTot)/sum(table(THDataRCS$THPostTot))
perPostRCS <- propPostRCS*100
perPostRCS
## Remove 6 From Trophy Hunting Post
THDataRCS$THPostTot[THDataRCS$THPostTot == 6] <- 3
THDataRCS$THPostTot <- as.numeric(THDataRCS$THPostTot)
table(THDataRCS$THPostTot)
mean(THDataRCS$THPostTot, na.rm = TRUE)
sd(THDataRCS$THPostTot, na.rm = TRUE)
median(THDataRCS$THPostTot, na.rm = TRUE)

###Difference
t.test(THDataRCS$THPreTot, THDataRCS$THPostTot, paired = TRUE)
##Effect Size
library(effsize)
cohen.d(THDataRCS$THPreTot, THDataRCS$THPostTot, paired = TRUE, within = FALSE)

###Test Difference Using Wilcoxon Signed Rank Test
wilcox.test(THDataRCS$THPreTot, THDataRCS$THPostTot, paired = TRUE)
```

```
###Effect Size Rank Biserial Correlation
library(effectsize)
rank_biserial(THDataRCS$THPreTot, THDataRCS$THPostTot, paired = TRUE, ci = 0.95)

##Message 8: Social Identity Socioeconomic
#Remove Other Versions
THDataRSS<- subset(THDataRight, Version != 1)
THDataRSS<- subset(THDataRSS, Version != 2)
THDataRSS<- subset(THDataRSS, Version != 3)
THDataRSS<- subset(THDataRSS, Version != 4)
THDataRSS<- subset(THDataRSS, Version != 5)
THDataRSS<- subset(THDataRSS, Version != 6)
THDataRSS<- subset(THDataRSS, Version != 7)
THDataRSS<- subset(THDataRSS, Version != 9)
table(THDataRSS$Version)

#Get Pre and Post Values
###Approval Pre Message Summary
table(THDataRSS$THPreTot)
length(THDataRSS$THPreTot)
propPreRSS<- table(THDataRSS$THPreTot)/sum(table(THDataRSS$THPreTot))
perPreRSS <- propPreRSS*100
perPreRSS
## Remove 6 From Trophy Hunting Pre
THDataRSS$THPreTot[THDataRSS$THPreTot == 6] <- 3
THDataRSS$THPreTot <- as.numeric(THDataRSS$THPreTot)
table(THDataRSS$THPreTot)
mean(THDataRSS$THPreTot, na.rm = TRUE)
sd(THDataRSS$THPreTot, na.rm = TRUE)
median(THDataRSS$THPreTot, na.rm = TRUE)

###Approval Post Message Summary
table(THDataRSS$THPostTot)
length(THDataRSS$THPostTot)
propPostRSS<- table(THDataRSS$THPostTot)/sum(table(THDataRSS$THPostTot))
perPostRSS <- propPostRSS*100
perPostRSS
## Remove 6 From Trophy Hunting Post
THDataRSS$THPostTot[THDataRSS$THPostTot == 6] <- 3
THDataRSS$THPostTot <- as.numeric(THDataRSS$THPostTot)
table(THDataRSS$THPostTot)
mean(THDataRSS$THPostTot, na.rm = TRUE)
sd(THDataRSS$THPostTot, na.rm = TRUE)
median(THDataRSS$THPostTot, na.rm = TRUE)

###Difference
t.test(THDataRSS$THPreTot, THDataRSS$THPostTot, paired = TRUE)
##Effect Size
library(effsize)
cohen.d(THDataRSS$THPreTot, THDataRSS$THPostTot, paired = TRUE, within = FALSE)

###Test Difference Using Wilcoxon Signed Rank Test
wilcox.test(THDataRSS$THPreTot, THDataRSS$THPostTot, paired = TRUE)

###Effect Size Rank Biserial Correlation
library(effectsize)
rank_biserial(THDataRSS$THPreTot, THDataRSS$THPostTot, paired = TRUE, ci = 0.95)

##Message 9: Value Socioeconomic
#Remove Other Versions
THDataRVS<- subset(THDataRight, Version != 1)
THDataRVS<- subset(THDataRVS, Version != 2)
THDataRVS<- subset(THDataRVS, Version != 3)
THDataRVS<- subset(THDataRVS, Version != 4)
```

```
THDataRVS<- subset(THDataRVS, Version != 5)
THDataRVS<- subset(THDataRVS, Version != 6)
THDataRVS<- subset(THDataRVS, Version != 7)
THDataRVS<- subset(THDataRVS, Version != 8)
table(THDataRVS$Version)

#Get Pre and Post Values
###Approval Pre Message Summary
table(THDataRVS$THPreTot)
length(THDataRVS$THPreTot)
propPreRVS<- table(THDataRVS$THPreTot)/sum(table(THDataRVS$THPreTot))
perPreRVS <- propPreRVS*100
perPreRVS
## Remove 6 From Trophy Hunting Pre
THDataRVS$THPreTot[THDataRVS$THPreTot == 6] <- 3
THDataRVS$THPreTot <- as.numeric(THDataRVS$THPreTot)
table(THDataRVS$THPreTot)
mean(THDataRVS$THPreTot, na.rm = TRUE)
sd(THDataRVS$THPreTot, na.rm = TRUE)
median(THDataRVS$THPreTot, na.rm = TRUE)

###Approval Post Message Summary
table(THDataRVS$THPostTot)
length(THDataRVS$THPostTot)
propPostRVS<- table(THDataRVS$THPostTot)/sum(table(THDataRVS$THPostTot))
perPostRVS <- propPostRVS*100
perPostRVS
## Remove 6 From Trophy Hunting Post
THDataRVS$THPostTot[THDataRVS$THPostTot == 6] <- 3
THDataRVS$THPostTot <- as.numeric(THDataRVS$THPostTot)
table(THDataRVS$THPostTot)
mean(THDataRVS$THPostTot, na.rm = TRUE)
sd(THDataRVS$THPostTot, na.rm = TRUE)
median(THDataRVS$THPostTot, na.rm = TRUE)

###Difference
t.test(THDataRVS$THPreTot, THDataRVS$THPostTot, paired = TRUE)
##Effect Size
library(effsize)
cohen.d(THDataRVS$THPreTot, THDataRVS$THPostTot, paired = TRUE, within = FALSE)

###Test Difference Using Wilcoxon Signed Rank Test
wilcox.test(THDataRVS$THPreTot, THDataRVS$THPostTot, paired = TRUE)

###Effect Size Rank Biserial Correlation
library(effectsize)
rank_biserial(THDataRVS$THPreTot, THDataRVS$THPostTot, paired = TRUE, ci = 0.95)

###Create Liberal Only Sample
THDataLeft<- subset(THData, PoliticsTot != 1)
table(THDataLeft$PoliticsTot)

########Difference in TH Attitudes Pre and Post Message

######Pre Message
###Approval Pre Message Summary
table(THDataLeft$THPreTot)
length(THDataLeft$THPreTot)
propPreAllL<- table(THDataLeft$THPreTot)/sum(table(THDataLeft$THPreTot))
perPreAllL <- propPreAllL*100
perPreAllL
THDataLeft$THPreTot <- as.numeric(THDataLeft$THPreTot)

#Get TH Approval by Message
table(THDataLeft$THPreTot, THDataLeft$Version)
```

```
## Remove 6 From Trophy Hunting Pre
THDataLeft$THPreTot[THDataLeft$THPreTot == 6] <- 3
table(THDataLeft$THPreTot)
#Get TH Approval by Message
table(THDataLeft$THPreTot, THDataLeft$Version)
aggregate(THPreTot ~ Version, data = THDataLeft, FUN = mean)
aggregate(THPreTot ~ Version, data = THDataLeft, FUN = sd)
aggregate(THPreTot ~ Version, data = THDataLeft, FUN = median)
mean(THDataLeft$THPreTot, na.rm = TRUE)
sd(THDataLeft$THPreTot, na.rm = TRUE)
median(THDataLeft$THPreTot, na.rm = TRUE)

##Determine Differences by Message Group
##ANOVA Model
LeftPreMesMod <- aov(THPreTot ~ Version, data = THDataLeft)
summary(LeftPreMesMod)

library(effectsize)
eta_squared(LeftPreMesMod, partial = FALSE)

## Check normality of the residuals with the Shapiro-Wilk test
shapiro.test(LeftPreMesMod$residuals)
###Check for equality of variance
library(car)
leveneTest(THPreTot ~Version,
           data = THDataLeft)

##Kruskal Wallis Test
library(rstatix)
kruskal.test(THPreTot ~ Version, data = THDataLeft)

#####Post Message
###Approval Post Message Summary
table(THDataLeft$THPostTot)
length(THDataLeft$THPostTot)
propPostAllL<- table(THDataLeft$THPostTot)/sum(table(THDataLeft$THPostTot))
perPostAllL <- propPostAllL*100
perPostAllL
THDataLeft$THPostTot <- as.numeric(THDataLeft$THPostTot)
#Get TH Approval by Message
table(THDataLeft$THPostTot, THDataLeft$Version)

## Remove 6 From Trophy Hunting Post
THDataLeft$THPostTot[THDataLeft$THPostTot == 6] <- 3
table(THDataLeft$THPostTot)
#Get TH Approval by Message
table(THDataLeft$THPostTot, THDataLeft$Version)
aggregate(THPostTot ~ Version, data = THDataLeft, FUN = mean)
aggregate(THPostTot ~ Version, data = THDataLeft, FUN = sd)
aggregate(THPostTot ~ Version, data = THDataLeft, FUN = median)
mean(THDataLeft$THPostTot, na.rm = TRUE)
sd(THDataLeft$THPostTot, na.rm = TRUE)
median(THDataLeft$THPostTot, na.rm = TRUE)

##Determine Differences by Message Group
##ANOVA Model
LeftPostMesMod <- aov(THPostTot ~ Version, data = THDataLeft)
summary(LeftPostMesMod)

library(effectsize)
eta_squared(LeftPostMesMod, partial = FALSE)

##Bonferroni
pairwise.t.test(THDataLeft$THPostTot, THDataLeft$Version, p.adj = "bonf")
```

```
##Make table with Bonf significance
LeftAllPostApp <- aggregate(THPostTot ~ Version, data = THDataLeft, FUN = mean)
LeftAllPostApp
#define new column
BonSigAllPostLeft <- c('ab','ab','a', 'ab', 'ab', 'ab', 'b', 'ab', 'ab')
#add column
LeftAllPostSig <- cbind(LeftAllPostApp, BonSigAllPostLeft)
LeftAllPostSig

## Check normality of the residuals with the Shapiro-Wilk test
shapiro.test(LeftPostMesMod$residuals)
###Check for equality of variance
library(car)
leveneTest(THPostTot ~Version,
           data = THDataLeft)

##Kruskal Wallis Test
library(rstatix)
kruskal.test(THPostTot ~ Version, data = THDataLeft)

# pairwise using dunn test
DunnLeftPost <- dunn_test(THPostTot ~ Version, data = THDataLeft,
                          p.adjust = "bonferroni")
DunnLeftPost
print(DunnLeftPost, n = 36)

##Make table with Bonf significance
NPLeftAllPostApp <- aggregate(THPostTot ~ Version, data = THDataLeft,
                              FUN = median)
NPLeftAllPostApp
#define new column to add
NPBonSigAllPostLeft <- c('ab','ab','a', 'ab', 'ab', 'ab', 'b', 'ab', 'ab')
#add column called 'new'
NPLeftAllPostSig <- cbind(NPLeftAllPostApp, NPBonSigAllPostLeft)
#view new data frame
NPLeftAllPostSig

##Separate by Message
table(THDataLeft$Version)

##Message 1: Control Control
#Remove Other Versions
THDataLCC<- subset(THDataLeft, Version != 2)
THDataLCC<- subset(THDataLCC, Version != 3)
THDataLCC<- subset(THDataLCC, Version != 4)
THDataLCC<- subset(THDataLCC, Version != 5)
THDataLCC<- subset(THDataLCC, Version != 6)
THDataLCC<- subset(THDataLCC, Version != 7)
THDataLCC<- subset(THDataLCC, Version != 8)
THDataLCC<- subset(THDataLCC, Version != 9)
table(THDataLCC$Version)

#Get Pre and Post Values

###Approval Pre Message Summary
table(THDataLCC$THPreTot)
length(THDataLCC$THPreTot)
propPreLCC<- table(THDataLCC$THPreTot)/sum(table(THDataLCC$THPreTot))
perPreLCC <- propPreLCC*100
perPreLCC
## Remove 6 From Trophy Hunting Pre
THDataLCC$THPreTot[THDataLCC$THPreTot == 6] <- 3
THDataLCC$THPreTot <- as.numeric(THDataLCC$THPreTot)
table(THDataLCC$THPreTot)
```

```
length(THDataLCC$THPreTot)
mean(THDataLCC$THPreTot, na.rm = TRUE)
sd(THDataLCC$THPreTot, na.rm = TRUE)
median(THDataLCC$THPreTot, na.rm = TRUE)

###Approval Post Message Summary
table(THDataLCC$THPostTot)
length(THDataLCC$THPostTot)
propPostLCC<- table(THDataLCC$THPostTot)/sum(table(THDataLCC$THPostTot))
perPostLCC <- propPostLCC*100
perPostLCC
## Remove 6 From Trophy Hunting Post
THDataLCC$THPostTot[THDataLCC$THPostTot == 6] <- 3
THDataLCC$THPostTot <- as.numeric(THDataLCC$THPostTot)
table(THDataLCC$THPostTot)
mean(THDataLCC$THPostTot, na.rm = TRUE)
sd(THDataLCC$THPostTot, na.rm = TRUE)
median(THDataLCC$THPostTot, na.rm = TRUE)

###Difference
t.test(THDataLCC$THPreTot, THDataLCC$THPostTot, paired = TRUE)
##Effect Size
library(effsize)
cohen.d(THDataLCC$THPreTot, THDataLCC$THPostTot, paired = TRUE, within = FALSE)

###Test Difference Using Wilcoxon Signed Rank Test
wilcox.test(THDataLCC$THPreTot, THDataLCC$THPostTot, paired = TRUE)

###Effect Size Rank Biserial Correlation
library(effectsize)
rank_biserial(THDataLCC$THPreTot, THDataLCC$THPostTot, paired = TRUE, ci = 0.95)

##Message 2: Social Identity Control
#Remove Other Versions
THDataLSC<- subset(THDataLeft, Version != 1)
THDataLSC<- subset(THDataLSC, Version != 3)
THDataLSC<- subset(THDataLSC, Version != 4)
THDataLSC<- subset(THDataLSC, Version != 5)
THDataLSC<- subset(THDataLSC, Version != 6)
THDataLSC<- subset(THDataLSC, Version != 7)
THDataLSC<- subset(THDataLSC, Version != 8)
THDataLSC<- subset(THDataLSC, Version != 9)
table(THDataLSC$Version)

#Get Pre and Post Values

###Approval Pre Message Summary
table(THDataLSC$THPreTot)
length(THDataLSC$THPreTot)
propPreLSC<- table(THDataLSC$THPreTot)/sum(table(THDataLSC$THPreTot))
perPreLSC <- propPreLSC*100
perPreLSC
## Remove 6 From Trophy Hunting Pre
THDataLSC$THPreTot[THDataLSC$THPreTot == 6] <- 3
THDataLSC$THPreTot <- as.numeric(THDataLSC$THPreTot)
table(THDataLSC$THPreTot)
mean(THDataLSC$THPreTot, na.rm = TRUE)
sd(THDataLSC$THPreTot, na.rm = TRUE)
median(THDataLSC$THPreTot, na.rm = TRUE)

###Approval Post Message Summary
table(THDataLSC$THPostTot)
length(THDataLSC$THPostTot)
propPostLSC<- table(THDataLSC$THPostTot)/sum(table(THDataLSC$THPostTot))
perPostLSC <- propPostLSC*100
```

```
perPostLSC
## Remove 6 From Trophy Hunting Post
THDataLSC$THPostTot[THDataLSC$THPostTot == 6] <- 3
THDataLSC$THPostTot <- as.numeric(THDataLSC$THPostTot)
table(THDataLSC$THPostTot)
mean(THDataLSC$THPostTot, na.rm = TRUE)
sd(THDataLSC$THPostTot, na.rm = TRUE)
median(THDataLSC$THPostTot, na.rm = TRUE)

###Difference
t.test(THDataLSC$THPreTot, THDataLSC$THPostTot, paired = TRUE)
##Effect Size
library(effsize)
cohen.d(THDataLSC$THPreTot, THDataLSC$THPostTot, paired = TRUE, within = FALSE)

###Test Difference Using Wilcoxon Signed Rank Test
wilcox.test(THDataLSC$THPreTot, THDataLSC$THPostTot, paired = TRUE)

###Effect Size Rank Biserial Correlation
library(effectsize)
rank_biserial(THDataLSC$THPreTot, THDataLSC$THPostTot, paired = TRUE, ci = 0.95)

##Message 3: Value Control
#Remove Other Versions
THDataLVC<- subset(THDataLeft, Version != 1)
THDataLVC<- subset(THDataLVC, Version != 2)
THDataLVC<- subset(THDataLVC, Version != 4)
THDataLVC<- subset(THDataLVC, Version != 5)
THDataLVC<- subset(THDataLVC, Version != 6)
THDataLVC<- subset(THDataLVC, Version != 7)
THDataLVC<- subset(THDataLVC, Version != 8)
THDataLVC<- subset(THDataLVC, Version != 9)
table(THDataLVC$Version)

#Get Pre and Post Values

###Approval Pre Message Summary
table(THDataLVC$THPreTot)
length(THDataLVC$THPreTot)
propPreLVC<- table(THDataLVC$THPreTot)/sum(table(THDataLVC$THPreTot))
perPreLVC <- propPreLVC*100
perPreLVC
## Remove 6 From Trophy Hunting Pre
THDataLVC$THPreTot[THDataLVC$THPreTot == 6] <- 3
THDataLVC$THPreTot <- as.numeric(THDataLVC$THPreTot)
table(THDataLVC$THPreTot)
mean(THDataLVC$THPreTot, na.rm = TRUE)
sd(THDataLVC$THPreTot, na.rm = TRUE)
median(THDataLVC$THPreTot, na.rm = TRUE)

###Approval Post Message Summary
table(THDataLVC$THPostTot)
length(THDataLVC$THPostTot)
propPostLVC<- table(THDataLVC$THPostTot)/sum(table(THDataLVC$THPostTot))
perPostLVC <- propPostLVC*100
perPostLVC
## Remove 6 From Trophy Hunting Post
THDataLVC$THPostTot[THDataLVC$THPostTot == 6] <- 3
THDataLVC$THPostTot <- as.numeric(THDataLVC$THPostTot)
table(THDataLVC$THPostTot)
mean(THDataLVC$THPostTot, na.rm = TRUE)
sd(THDataLVC$THPostTot, na.rm = TRUE)
median(THDataLVC$THPostTot, na.rm = TRUE)

###Difference
```

```
t.test(THDataLVC$THPreTot, THDataLVC$THPostTot, paired = TRUE)
##Effect Size
library(effsize)
cohen.d(THDataLVC$THPreTot, THDataLVC$THPostTot, paired = TRUE, within = FALSE)

###Test Difference Using Wilcoxon Signed Rank Test
wilcox.test(THDataLVC$THPreTot, THDataLVC$THPostTot, paired = TRUE)

###Effect Size Rank Biserial Correlation
library(effectsize)
rank_biserial(THDataLVC$THPreTot, THDataLVC$THPostTot, paired = TRUE, ci = 0.95)

##Message 4: Control Wildlife
#Remove Other Versions
THDataLCW<- subset(THDataLeft, Version != 1)
THDataLCW<- subset(THDataLCW, Version != 2)
THDataLCW<- subset(THDataLCW, Version != 3)
THDataLCW<- subset(THDataLCW, Version != 5)
THDataLCW<- subset(THDataLCW, Version != 6)
THDataLCW<- subset(THDataLCW, Version != 7)
THDataLCW<- subset(THDataLCW, Version != 8)
THDataLCW<- subset(THDataLCW, Version != 9)
table(THDataLCW$Version)

#Get Pre and Post Values

###Approval Pre Message Summary
table(THDataLCW$THPreTot)
length(THDataLCW$THPreTot)
propPreLCW<- table(THDataLCW$THPreTot)/sum(table(THDataLCW$THPreTot))
perPreLCW <- propPreLCW*100
perPreLCW
## Remove 6 From Trophy Hunting Pre
THDataLCW$THPreTot[THDataLCW$THPreTot == 6] <- 3
THDataLCW$THPreTot <- as.numeric(THDataLCW$THPreTot)
table(THDataLCW$THPreTot)
mean(THDataLCW$THPreTot, na.rm = TRUE)
sd(THDataLCW$THPreTot, na.rm = TRUE)
median(THDataLCW$THPreTot, na.rm = TRUE)

###Approval Post Message Summary
table(THDataLCW$THPostTot)
length(THDataLCW$THPostTot)
propPostLCW<- table(THDataLCW$THPostTot)/sum(table(THDataLCW$THPostTot))
perPostLCW <- propPostLCW*100
perPostLCW
## Remove 6 From Trophy Hunting Post
THDataLCW$THPostTot[THDataLCW$THPostTot == 6] <- 3
THDataLCW$THPostTot <- as.numeric(THDataLCW$THPostTot)
table(THDataLCW$THPostTot)
mean(THDataLCW$THPostTot, na.rm = TRUE)
sd(THDataLCW$THPostTot, na.rm = TRUE)
median(THDataLCW$THPostTot, na.rm = TRUE)

###Difference
t.test(THDataLCW$THPreTot, THDataLCW$THPostTot, paired = TRUE)
##Effect Size
library(effsize)
cohen.d(THDataLCW$THPreTot, THDataLCW$THPostTot, paired = TRUE, within = FALSE)

###Test Difference Using Wilcoxon Signed Rank Test
wilcox.test(THDataLCW$THPreTot, THDataLCW$THPostTot, paired = TRUE)

###Effect Size Rank Biserial Correlation
library(effectsize)
```

```
rank_biserial(THDataLCW$THPreTot, THDataLCW$THPostTot, paired = TRUE, ci = 0.95)

##Message 5: Social Identity Wildlife
#Remove Other Versions
THDataLSW<- subset(THDataLeft, Version != 1)
THDataLSW<- subset(THDataLSW, Version != 2)
THDataLSW<- subset(THDataLSW, Version != 3)
THDataLSW<- subset(THDataLSW, Version != 4)
THDataLSW<- subset(THDataLSW, Version != 6)
THDataLSW<- subset(THDataLSW, Version != 7)
THDataLSW<- subset(THDataLSW, Version != 8)
THDataLSW<- subset(THDataLSW, Version != 9)
table(THDataLSW$Version)

#Get Pre and Post Values

###Approval Pre Message Summary
table(THDataLSW$THPreTot)
length(THDataLSW$THPreTot)
propPreLSW<- table(THDataLSW$THPreTot)/sum(table(THDataLSW$THPreTot))
perPreLSW <- propPreLSW*100
perPreLSW
## Remove 6 From Trophy Hunting Pre
THDataLSW$THPreTot[THDataLSW$THPreTot == 6] <- 3
THDataLSW$THPreTot <- as.numeric(THDataLSW$THPreTot)
table(THDataLSW$THPreTot)
mean(THDataLSW$THPreTot, na.rm = TRUE)
sd(THDataLSW$THPreTot, na.rm = TRUE)
median(THDataLSW$THPreTot, na.rm = TRUE)

###Approval Post Message Summary
table(THDataLSW$THPostTot)
length(THDataLSW$THPostTot)
propPostLSW<- table(THDataLSW$THPostTot)/sum(table(THDataLSW$THPostTot))
perPostLSW <- propPostLSW*100
perPostLSW
## Remove 6 From Trophy Hunting Post
THDataLSW$THPostTot[THDataLSW$THPostTot == 6] <- 3
THDataLSW$THPostTot <- as.numeric(THDataLSW$THPostTot)
table(THDataLSW$THPostTot)
mean(THDataLSW$THPostTot, na.rm = TRUE)
sd(THDataLSW$THPostTot, na.rm = TRUE)
median(THDataLSW$THPostTot, na.rm = TRUE)

###Difference
t.test(THDataLSW$THPreTot, THDataLSW$THPostTot, paired = TRUE)
##Effect Size
library(effsize)
cohen.d(THDataLSW$THPreTot, THDataLSW$THPostTot, paired = TRUE, within = FALSE)

###Test Difference Using Wilcoxon Signed Rank Test
wilcox.test(THDataLSW$THPreTot, THDataLSW$THPostTot, paired = TRUE)

###Effect Size Rank Biserial Correlation
library(effectsize)
rank_biserial(THDataLSW$THPreTot, THDataLSW$THPostTot, paired = TRUE, ci = 0.95)

##Message 6: Value Wildlife
#Remove Other Versions
THDataLVW<- subset(THDataLeft, Version != 1)
THDataLVW<- subset(THDataLVW, Version != 2)
THDataLVW<- subset(THDataLVW, Version != 3)
THDataLVW<- subset(THDataLVW, Version != 4)
THDataLVW<- subset(THDataLVW, Version != 5)
THDataLVW<- subset(THDataLVW, Version != 7)
```

```
THDataLVW<- subset(THDataLVW, Version != 8)
THDataLVW<- subset(THDataLVW, Version != 9)
table(THDataLVW$Version)

#Get Pre and Post Values

###Approval Pre Message Summary
table(THDataLVW$THPreTot)
length(THDataLVW$THPreTot)
propPreLVW<- table(THDataLVW$THPreTot)/sum(table(THDataLVW$THPreTot))
perPreLVW <- propPreLVW*100
perPreLVW
## Remove 6 From Trophy Hunting Pre
THDataLVW$THPreTot[THDataLVW$THPreTot == 6] <- 3
THDataLVW$THPreTot <- as.numeric(THDataLVW$THPreTot)
table(THDataLVW$THPreTot)
mean(THDataLVW$THPreTot, na.rm = TRUE)
sd(THDataLVW$THPreTot, na.rm = TRUE)
median(THDataLVW$THPreTot, na.rm = TRUE)

###Approval Post Message Summary
table(THDataLVW$THPostTot)
length(THDataLVW$THPostTot)
propPostLVW<- table(THDataLVW$THPostTot)/sum(table(THDataLVW$THPostTot))
perPostLVW <- propPostLVW*100
perPostLVW
## Remove 6 From Trophy Hunting Post
THDataLVW$THPostTot[THDataLVW$THPostTot == 6] <- 3
THDataLVW$THPostTot <- as.numeric(THDataLVW$THPostTot)
table(THDataLVW$THPostTot)
mean(THDataLVW$THPostTot, na.rm = TRUE)
sd(THDataLVW$THPostTot, na.rm = TRUE)
median(THDataLVW$THPostTot, na.rm = TRUE)

###Difference
t.test(THDataLVW$THPreTot, THDataLVW$THPostTot, paired = TRUE)
##Effect Size
library(effsize)
cohen.d(THDataLVW$THPreTot, THDataLVW$THPostTot, paired = TRUE, within = FALSE)

###Test Difference Using Wilcoxon Signed Rank Test
wilcox.test(THDataLVW$THPreTot, THDataLVW$THPostTot, paired = TRUE)

###Effect Size Rank Biserial Correlation
library(effectsize)
rank_biserial(THDataLVW$THPreTot, THDataLVW$THPostTot, paired = TRUE, ci = 0.95)

##Message 7: Control Socioeconomic
#Remove Other Versions
THDataLCS<- subset(THDataLeft, Version != 1)
THDataLCS<- subset(THDataLCS, Version != 2)
THDataLCS<- subset(THDataLCS, Version != 3)
THDataLCS<- subset(THDataLCS, Version != 4)
THDataLCS<- subset(THDataLCS, Version != 5)
THDataLCS<- subset(THDataLCS, Version != 6)
THDataLCS<- subset(THDataLCS, Version != 8)
THDataLCS<- subset(THDataLCS, Version != 9)
table(THDataLCS$Version)

#Get Pre and Post Values

###Approval Pre Message Summary
table(THDataLCS$THPreTot)
length(THDataLCS$THPreTot)
propPreLCS<- table(THDataLCS$THPreTot)/sum(table(THDataLCS$THPreTot))
```

```
perPreLCS <- propPreLCS*100
perPreLCS
## Remove 6 From Trophy Hunting Pre
THDataLCS$THPreTot[THDataLCS$THPreTot == 6] <- 3
THDataLCS$THPreTot <- as.numeric(THDataLCS$THPreTot)
table(THDataLCS$THPreTot)
mean(THDataLCS$THPreTot, na.rm = TRUE)
sd(THDataLCS$THPreTot, na.rm = TRUE)
median(THDataLCS$THPreTot, na.rm = TRUE)

###Approval Post Message Summary
table(THDataLCS$THPostTot)
length(THDataLCS$THPostTot)
propPostLCS<- table(THDataLCS$THPostTot)/sum(table(THDataLCS$THPostTot))
perPostLCS <- propPostLCS*100
perPostLCS
## Remove 6 From Trophy Hunting Post
THDataLCS$THPostTot[THDataLCS$THPostTot == 6] <- 3
THDataLCS$THPostTot <- as.numeric(THDataLCS$THPostTot)
table(THDataLCS$THPostTot)
mean(THDataLCS$THPostTot, na.rm = TRUE)
sd(THDataLCS$THPostTot, na.rm = TRUE)
median(THDataLCS$THPostTot, na.rm = TRUE)

###Difference
t.test(THDataLCS$THPreTot, THDataLCS$THPostTot, paired = TRUE)
##Effect Size
library(effsize)
cohen.d(THDataLCS$THPreTot, THDataLCS$THPostTot, paired = TRUE, within = FALSE)

###Test Difference Using Wilcoxon Signed Rank Test
wilcox.test(THDataLCS$THPreTot, THDataLCS$THPostTot, paired = TRUE)

###Effect Size Rank Biserial Correlation
library(effectsize)
rank_biserial(THDataLCS$THPreTot, THDataLCS$THPostTot, paired = TRUE, ci = 0.95)

##Message 8: Social Identity Socioeconomic
#Remove Other Versions
THDataLSS<- subset(THDataLeft, Version != 1)
THDataLSS<- subset(THDataLSS, Version != 2)
THDataLSS<- subset(THDataLSS, Version != 3)
THDataLSS<- subset(THDataLSS, Version != 4)
THDataLSS<- subset(THDataLSS, Version != 5)
THDataLSS<- subset(THDataLSS, Version != 6)
THDataLSS<- subset(THDataLSS, Version != 7)
THDataLSS<- subset(THDataLSS, Version != 9)
table(THDataLSS$Version)

#Get Pre and Post Values

###Approval Pre Message Summary
table(THDataLSS$THPreTot)
length(THDataLSS$THPreTot)
propPreLSS<- table(THDataLSS$THPreTot)/sum(table(THDataLSS$THPreTot))
perPreLSS <- propPreLSS*100
perPreLSS
## Remove 6 From Trophy Hunting Pre
THDataLSS$THPreTot[THDataLSS$THPreTot == 6] <- 3
THDataLSS$THPreTot <- as.numeric(THDataLSS$THPreTot)
table(THDataLSS$THPreTot)
mean(THDataLSS$THPreTot, na.rm = TRUE)
sd(THDataLSS$THPreTot, na.rm = TRUE)
median(THDataLSS$THPreTot, na.rm = TRUE)
```

```
###Approval Post Message Summary
table(THDataLSS$THPostTot)
length(THDataLSS$THPostTot)
propPostLSS<- table(THDataLSS$THPostTot)/sum(table(THDataLSS$THPostTot))
perPostLSS <- propPostLSS*100
perPostLSS
## Remove 6 From Trophy Hunting Post
THDataLSS$THPostTot[THDataLSS$THPostTot == 6] <- 3
THDataLSS$THPostTot <- as.numeric(THDataLSS$THPostTot)
table(THDataLSS$THPostTot)
mean(THDataLSS$THPostTot, na.rm = TRUE)
sd(THDataLSS$THPostTot, na.rm = TRUE)
median(THDataLSS$THPostTot, na.rm = TRUE)

###Difference
t.test(THDataLSS$THPreTot, THDataLSS$THPostTot, paired = TRUE)
##Effect Size
library(effsize)
cohen.d(THDataLSS$THPreTot, THDataLSS$THPostTot, paired = TRUE, within = FALSE)

###Test Difference Using Wilcoxon Signed Rank Test
wilcox.test(THDataLSS$THPreTot, THDataLSS$THPostTot, paired = TRUE)

###Effect Size Rank Biserial Correlation
library(effectsize)
rank_biserial(THDataLSS$THPreTot, THDataLSS$THPostTot, paired = TRUE, ci = 0.95)

##Message 9: Value Socioeconomic
#Remove Other Versions
THDataLVS<- subset(THDataLeft, Version != 1)
THDataLVS<- subset(THDataLVS, Version != 2)
THDataLVS<- subset(THDataLVS, Version != 3)
THDataLVS<- subset(THDataLVS, Version != 4)
THDataLVS<- subset(THDataLVS, Version != 5)
THDataLVS<- subset(THDataLVS, Version != 6)
THDataLVS<- subset(THDataLVS, Version != 7)
THDataLVS<- subset(THDataLVS, Version != 8)
table(THDataLVS$Version)

#Get Pre and Post Values

###Approval Pre Message Summary
table(THDataLVS$THPreTot)
length(THDataLVS$THPreTot)
propPreLVS<- table(THDataLVS$THPreTot)/sum(table(THDataLVS$THPreTot))
perPreLVS <- propPreLVS*100
perPreLVS
## Remove 6 From Trophy Hunting Pre
THDataLVS$THPreTot[THDataLVS$THPreTot == 6] <- 3
THDataLVS$THPreTot <- as.numeric(THDataLVS$THPreTot)
table(THDataLVS$THPreTot)
mean(THDataLVS$THPreTot, na.rm = TRUE)
sd(THDataLVS$THPreTot, na.rm = TRUE)
median(THDataLVS$THPreTot, na.rm = TRUE)

###Approval Post Message Summary
table(THDataLVS$THPostTot)
length(THDataLVS$THPostTot)
propPostLVS<- table(THDataLVS$THPostTot)/sum(table(THDataLVS$THPostTot))
perPostLVS <- propPostLVS*100
perPostLVS
## Remove 6 From Trophy Hunting Post
THDataLVS$THPostTot[THDataLVS$THPostTot == 6] <- 3
THDataLVS$THPostTot <- as.numeric(THDataLVS$THPostTot)
table(THDataLVS$THPostTot)
```

```
mean(THDataLVS$THPostTot, na.rm = TRUE)
sd(THDataLVS$THPostTot, na.rm = TRUE)
median(THDataLVS$THPostTot, na.rm = TRUE)

###Difference
t.test(THDataLVS$THPreTot, THDataLVS$THPostTot, paired = TRUE)
##Effect Size
library(effsize)
cohen.d(THDataLVS$THPreTot, THDataLVS$THPostTot, paired = TRUE, within = FALSE)

###Test Difference Using Wilcoxon Signed Rank Test
wilcox.test(THDataLVS$THPreTot, THDataLVS$THPostTot, paired = TRUE)

###Effect Size Rank Biserial Correlation
library(effectsize)
rank_biserial(THDataLVS$THPreTot, THDataLVS$THPostTot, paired = TRUE, ci = 0.95)

#####Values Detailed Analysis
###Determining effects of different messages on
#High Conservation and High Economic

##Conservation and Stewardship
THData$ValueConservationTot <- as.numeric(THData$ValueConservationTot)
Constw <- THData$ValueConservationTot
Constw
table(Constw)
length(Constw)

###Create High and Low Extremes
#High
THData$ValueConservationTot[THData$ValueConservationTot == 2] <- 1
#Low
THData$ValueConservationTot[THData$ValueConservationTot == 4] <- 2
THData$ValueConservationTot[THData$ValueConservationTot == 5] <- 2
#Exclude Middle
THData$ValueConservationTot[THData$ValueConservationTot == 3] <- NA
table(THData$ValueConservationTot)

#####Conservation and Stewardship Extremes
###Create Conservation High Rank Only Sample
THDataConH<- subset(THData, ValueConservationTot != 2)
table(THDataConH$ValueConservationTot)

########Difference in TH Attitudes Pre and Post Message
######Pre Message
###Approval Pre Message Summary
table(THDataConH$THPreTot)
length(THDataConH$THPreTot)
propPreAllConH<- table(THDataConH$THPreTot)/sum(table(THDataConH$THPreTot))
perPreAllConH <- propPreAllConH*100
perPreAllConH
THDataConH$THPreTot <- as.numeric(THDataConH$THPreTot)

#Get TH Approval by Message
table(THDataConH$THPreTot, THDataConH$Version)
aggregate(THPreTot ~ Version, data = THDataConH, FUN = mean)

## Remove 6 From Trophy Hunting Pre
THDataConH$THPreTot[THDataConH$THPreTot == 6] <- 3
table(THDataConH$THPreTot)
#Get TH Approval by Message
table(THDataConH$THPreTot, THDataConH$Version)
aggregate(THPreTot ~ Version, data = THDataConH, FUN = mean)
aggregate(THPreTot ~ Version, data = THDataConH, FUN = sd)
aggregate(THPreTot ~ Version, data = THDataConH, FUN = median)
```

```
mean(THDataConH$THPreTot, na.rm = TRUE)
sd(THDataConH$THPreTot, na.rm = TRUE)
median(THDataConH$THPreTot, na.rm = TRUE)

##Determine Differences by Message Group
##ANOVA Model
ConHPreMesMod <- aov(THPreTot ~ Version, data = THDataConH)
summary(ConHPreMesMod)

library(effectsize)
eta_squared(ConHPreMesMod, partial = FALSE)

## Check normality of the residuals with the Shapiro-Wilk test
shapiro.test(ConHPreMesMod$residuals)
###Check for equality of variance
library(car)
leveneTest(THPreTot ~Version,
           data = THDataConH)

##Test Differences Across Message Version
##Kruskal Wallis Test
library(rstatix)
kruskal.test(THPreTot ~ Version, data = THDataConH)

#####Post Message
###Approval Post Message Summary
table(THDataConH$THPostTot)
length(THDataConH$THPostTot)
propPostAllConH<- table(THDataConH$THPostTot)/sum(table(THDataConH$THPostTot))
perPostAllConH <- propPostAllConH*100
perPostAllConH
THDataConH$THPostTot <- as.numeric(THDataConH$THPostTot)
#Get TH Approval by Message
table(THDataConH$THPostTot, THDataConH$Version)
aggregate(THPostTot ~ Version, data = THDataConH, FUN = mean)

## Remove 6 From Trophy Hunting Post
THDataConH$THPostTot[THDataConH$THPostTot == 6] <- 3
table(THDataConH$THPostTot)
#Get TH Approval by Message
table(THDataConH$THPostTot, THDataConH$Version)
aggregate(THPostTot ~ Version, data = THDataConH, FUN = mean)
aggregate(THPostTot ~ Version, data = THDataConH, FUN = sd)
aggregate(THPostTot ~ Version, data = THDataConH, FUN = median)
mean(THDataConH$THPostTot, na.rm = TRUE)
sd(THDataConH$THPostTot, na.rm = TRUE)
median(THDataConH$THPostTot, na.rm = TRUE)

##Determine Differences by Message Group
##ANOVA Model
ConHPostMesMod <- aov(THPostTot ~ Version, data = THDataConH)
summary(ConHPostMesMod)

library(effectsize)
eta_squared(ConHPostMesMod, partial = FALSE)

##Bonferroni
pairwise.t.test(THDataConH$THPostTot, THDataConH$Version, p.adj = "bonf")

##Make table with Bonf significance
ConHAllPostApp <- aggregate(THPostTot ~ Version, data = THDataConH, FUN = mean)
ConHAllPostApp
#define new column
BonSigAllPostConH <- c('a','ab','ac', 'b', 'bc', 'bc', 'bc', 'ab', 'ab')
#add column
```

```
AllPostConHSig <- cbind(ConHAllPostApp, BonSigAllPostConH)
AllPostConHSig

## Check normality of the residuals with the Shapiro-Wilk test
shapiro.test(ConHPostMesMod$residuals)
###Check for equality of variance
library(car)
leveneTest(THPostTot ~Version,
           data = THDataConH)

##Kruskal Wallis Test
library(rstatix)
kruskal.test(THPostTot ~ Version, data = THDataConH)

# pairwise using dunn test
DunnConHPost <- dunn_test(THPostTot ~ Version, data = THDataConH,
                          p.adjust = "bonferroni")
DunnConHPost
print(DunnConHPost, n = 36)

##Make table with Bonf significance
NPConHAllPostApp <- aggregate(THPostTot ~ Version, data = THDataConH,
                              FUN = median)
NPConHAllPostApp
#define new column to add
NPBonSigAllPostConH <- c('a','abc','ac', 'b', 'bc', 'bc', 'bc', 'abc', 'abc')
#add column called 'new'
NPAllPostConHSig <- cbind(NPConHAllPostApp, NPBonSigAllPostConH)
#view new data frame
NPAllPostConHSig

##Separate by Message
table(THDataConH$Version)

##Message 1: Control Control
#Remove Other Versions
THDataCHCC<- subset(THDataConH, Version != 2)
THDataCHCC<- subset(THDataCHCC, Version != 3)
THDataCHCC<- subset(THDataCHCC, Version != 4)
THDataCHCC<- subset(THDataCHCC, Version != 5)
THDataCHCC<- subset(THDataCHCC, Version != 6)
THDataCHCC<- subset(THDataCHCC, Version != 7)
THDataCHCC<- subset(THDataCHCC, Version != 8)
THDataCHCC<- subset(THDataCHCC, Version != 9)
table(THDataCHCC$Version)

#Get Pre and Post Values
###Approval Pre Message Summary
table(THDataCHCC$THPreTot)
length(THDataCHCC$THPreTot)
propPreCHCC<- table(THDataCHCC$THPreTot)/sum(table(THDataCHCC$THPreTot))
perPreCHCC <- propPreCHCC*100
perPreCHCC
## Remove 6 From Trophy Hunting Pre
THDataCHCC$THPreTot[THDataCHCC$THPreTot == 6] <- 3
THDataCHCC$THPreTot <- as.numeric(THDataCHCC$THPreTot)
table(THDataCHCC$THPreTot)
length(THDataCHCC$THPreTot)
mean(THDataCHCC$THPreTot, na.rm = TRUE)
sd(THDataCHCC$THPreTot, na.rm = TRUE)
median(THDataCHCC$THPreTot, na.rm = TRUE)

###Approval Post Message Summary
table(THDataCHCC$THPostTot)
length(THDataCHCC$THPostTot)
```

```
propPostCHCC<- table(THDataCHCC$THPostTot)/sum(table(THDataCHCC$THPostTot))
perPostCHCC <- propPostCHCC*100
perPostCHCC
## Remove 6 From Trophy Hunting Post
THDataCHCC$THPostTot[THDataCHCC$THPostTot == 6] <- 3
THDataCHCC$THPostTot <- as.numeric(THDataCHCC$THPostTot)
table(THDataCHCC$THPostTot)
mean(THDataCHCC$THPostTot, na.rm = TRUE)
sd(THDataCHCC$THPostTot, na.rm = TRUE)
median(THDataCHCC$THPostTot, na.rm = TRUE)

###Difference
t.test(THDataCHCC$THPreTot, THDataCHCC$THPostTot, paired = TRUE)
##Effect Size
library(effsize)
cohen.d(THDataCHCC$THPreTot, THDataCHCC$THPostTot, paired = TRUE, within =
          FALSE)

###Test Difference Using Wilcoxon Signed Rank Test
wilcox.test(THDataCHCC$THPreTot, THDataCHCC$THPostTot, paired = TRUE)

###Effect Size Rank Biserial Correlation
library(effectsize)
rank_biserial(THDataCHCC$THPreTot, THDataCHCC$THPostTot, paired = TRUE,
              ci = 0.95)

##Message 2: Social Identity Control
#Remove Other Versions
THDataCHSC<- subset(THDataConH, Version != 1)
THDataCHSC<- subset(THDataCHSC, Version != 3)
THDataCHSC<- subset(THDataCHSC, Version != 4)
THDataCHSC<- subset(THDataCHSC, Version != 5)
THDataCHSC<- subset(THDataCHSC, Version != 6)
THDataCHSC<- subset(THDataCHSC, Version != 7)
THDataCHSC<- subset(THDataCHSC, Version != 8)
THDataCHSC<- subset(THDataCHSC, Version != 9)
table(THDataCHSC$Version)

#Get Pre and Post Values
###Approval Pre Message Summary
table(THDataCHSC$THPreTot)
length(THDataCHSC$THPreTot)
propPreCHSC<- table(THDataCHSC$THPreTot)/sum(table(THDataCHSC$THPreTot))
perPreCHSC <- propPreCHSC*100
perPreCHSC
## Remove 6 From Trophy Hunting Pre
THDataCHSC$THPreTot[THDataCHSC$THPreTot == 6] <- 3
THDataCHSC$THPreTot <- as.numeric(THDataCHSC$THPreTot)
table(THDataCHSC$THPreTot)
mean(THDataCHSC$THPreTot, na.rm = TRUE)
sd(THDataCHSC$THPreTot, na.rm = TRUE)
median(THDataCHSC$THPreTot, na.rm = TRUE)

###Approval Post Message Summary
table(THDataCHSC$THPostTot)
length(THDataCHSC$THPostTot)
propPostCHSC<- table(THDataCHSC$THPostTot)/sum(table(THDataCHSC$THPostTot))
perPostCHSC <- propPostCHSC*100
perPostCHSC
## Remove 6 From Trophy Hunting Post
THDataCHSC$THPostTot[THDataCHSC$THPostTot == 6] <- 3
THDataCHSC$THPostTot <- as.numeric(THDataCHSC$THPostTot)
table(THDataCHSC$THPostTot)
mean(THDataCHSC$THPostTot, na.rm = TRUE)
sd(THDataCHSC$THPostTot, na.rm = TRUE)
```

```
median(THDataCHSC$THPostTot, na.rm = TRUE)

###Difference
t.test(THDataCHSC$THPreTot, THDataCHSC$THPostTot, paired = TRUE)
##Effect Size
library(effsize)
cohen.d(THDataCHSC$THPreTot, THDataCHSC$THPostTot, paired = TRUE,
        within = FALSE)

###Test Difference Using Wilcoxon Signed Rank Test
wilcox.test(THDataCHSC$THPreTot, THDataCHSC$THPostTot, paired = TRUE)

###Effect Size Rank Biserial Correlation
library(effectsize)
rank_biserial(THDataCHSC$THPreTot, THDataCHSC$THPostTot, paired = TRUE,
              ci = 0.95)

##Message 3: Value Control
#Remove Other Versions
THDataCHVC<- subset(THDataConH, Version != 1)
THDataCHVC<- subset(THDataCHVC, Version != 2)
THDataCHVC<- subset(THDataCHVC, Version != 4)
THDataCHVC<- subset(THDataCHVC, Version != 5)
THDataCHVC<- subset(THDataCHVC, Version != 6)
THDataCHVC<- subset(THDataCHVC, Version != 7)
THDataCHVC<- subset(THDataCHVC, Version != 8)
THDataCHVC<- subset(THDataCHVC, Version != 9)
table(THDataCHVC$Version)

#Get Pre and Post Values
###Approval Pre Message Summary
table(THDataCHVC$THPreTot)
length(THDataCHVC$THPreTot)
propPreCHVC<- table(THDataCHVC$THPreTot)/sum(table(THDataCHVC$THPreTot))
perPreCHVC <- propPreCHVC*100
perPreCHVC
## Remove 6 From Trophy Hunting Pre
THDataCHVC$THPreTot[THDataCHVC$THPreTot == 6] <- 3
THDataCHVC$THPreTot <- as.numeric(THDataCHVC$THPreTot)
table(THDataCHVC$THPreTot)
mean(THDataCHVC$THPreTot, na.rm = TRUE)
sd(THDataCHVC$THPreTot, na.rm = TRUE)
median(THDataCHVC$THPreTot, na.rm = TRUE)

###Approval Post Message Summary
table(THDataCHVC$THPostTot)
length(THDataCHVC$THPostTot)
propPostCHVC<- table(THDataCHVC$THPostTot)/sum(table(THDataCHVC$THPostTot))
perPostCHVC <- propPostCHVC*100
perPostCHVC
## Remove 6 From Trophy Hunting Post
THDataCHVC$THPostTot[THDataCHVC$THPostTot == 6] <- 3
THDataCHVC$THPostTot <- as.numeric(THDataCHVC$THPostTot)
table(THDataCHVC$THPostTot)
mean(THDataCHVC$THPostTot, na.rm = TRUE)
sd(THDataCHVC$THPostTot, na.rm = TRUE)
median(THDataCHVC$THPostTot, na.rm = TRUE)

###Difference
t.test(THDataCHVC$THPreTot, THDataCHVC$THPostTot, paired = TRUE)
##Effect Size
library(effsize)
cohen.d(THDataCHVC$THPreTot, THDataCHVC$THPostTot, paired = TRUE,
        within = FALSE)
```

```
###Test Difference Using Wilcoxon Signed Rank Test
wilcox.test(THDataCHVC$THPreTot, THDataCHVC$THPostTot, paired = TRUE)

###Effect Size Rank Biserial Correlation
library(effectsize)
rank_biserial(THDataCHVC$THPreTot, THDataCHVC$THPostTot, paired = TRUE,
              ci = 0.95)

##Message 4: Control Wildlife
#Remove Other Versions
THDataCHCW<- subset(THDataConH, Version != 1)
THDataCHCW<- subset(THDataCHCW, Version != 2)
THDataCHCW<- subset(THDataCHCW, Version != 3)
THDataCHCW<- subset(THDataCHCW, Version != 5)
THDataCHCW<- subset(THDataCHCW, Version != 6)
THDataCHCW<- subset(THDataCHCW, Version != 7)
THDataCHCW<- subset(THDataCHCW, Version != 8)
THDataCHCW<- subset(THDataCHCW, Version != 9)
table(THDataCHCW$Version)

#Get Pre and Post Values
###Approval Pre Message Summary
table(THDataCHCW$THPreTot)
length(THDataCHCW$THPreTot)
propPreCHCW<- table(THDataCHCW$THPreTot)/sum(table(THDataCHCW$THPreTot))
perPreCHCW <- propPreCHCW*100
perPreCHCW
## Remove 6 From Trophy Hunting Pre
THDataCHCW$THPreTot[THDataCHCW$THPreTot == 6] <- 3
THDataCHCW$THPreTot <- as.numeric(THDataCHCW$THPreTot)
table(THDataCHCW$THPreTot)
mean(THDataCHCW$THPreTot, na.rm = TRUE)
sd(THDataCHCW$THPreTot, na.rm = TRUE)
median(THDataCHCW$THPreTot, na.rm = TRUE)

###Approval Post Message Summary
table(THDataCHCW$THPostTot)
length(THDataCHCW$THPostTot)
propPostCHCW<- table(THDataCHCW$THPostTot)/sum(table(THDataCHCW$THPostTot))
perPostCHCW <- propPostCHCW*100
perPostCHCW
## Remove 6 From Trophy Hunting Post
THDataCHCW$THPostTot[THDataCHCW$THPostTot == 6] <- 3
THDataCHCW$THPostTot <- as.numeric(THDataCHCW$THPostTot)
table(THDataCHCW$THPostTot)
mean(THDataCHCW$THPostTot, na.rm = TRUE)
sd(THDataCHCW$THPostTot, na.rm = TRUE)
median(THDataCHCW$THPostTot, na.rm = TRUE)

###Difference
t.test(THDataCHCW$THPreTot, THDataCHCW$THPostTot, paired = TRUE)
##Effect Size
library(effsize)
cohen.d(THDataCHCW$THPreTot, THDataCHCW$THPostTot, paired = TRUE,
        within = FALSE)

###Test Difference Using Wilcoxon Signed Rank Test
wilcox.test(THDataCHCW$THPreTot, THDataCHCW$THPostTot, paired = TRUE)

###Effect Size Rank Biserial Correlation
library(effectsize)
rank_biserial(THDataCHCW$THPreTot, THDataCHCW$THPostTot, paired = TRUE,
              ci = 0.95)

##Message 5: Social Identity Wildlife
```

```
#Remove Other Versions
THDataCHSW<- subset(THDataConH, Version != 1)
THDataCHSW<- subset(THDataCHSW, Version != 2)
THDataCHSW<- subset(THDataCHSW, Version != 3)
THDataCHSW<- subset(THDataCHSW, Version != 4)
THDataCHSW<- subset(THDataCHSW, Version != 6)
THDataCHSW<- subset(THDataCHSW, Version != 7)
THDataCHSW<- subset(THDataCHSW, Version != 8)
THDataCHSW<- subset(THDataCHSW, Version != 9)
table(THDataCHSW$Version)

#Get Pre and Post Values
###Approval Pre Message Summary
table(THDataCHSW$THPreTot)
length(THDataCHSW$THPreTot)
propPreCHSW<- table(THDataCHSW$THPreTot)/sum(table(THDataCHSW$THPreTot))
perPreCHSW <- propPreCHSW*100
perPreCHSW
## Remove 6 From Trophy Hunting Pre
THDataCHSW$THPreTot[THDataCHSW$THPreTot == 6] <- 3
THDataCHSW$THPreTot <- as.numeric(THDataCHSW$THPreTot)
table(THDataCHSW$THPreTot)
mean(THDataCHSW$THPreTot, na.rm = TRUE)
sd(THDataCHSW$THPreTot, na.rm = TRUE)
median(THDataCHSW$THPreTot, na.rm = TRUE)

###Approval Post Message Summary
table(THDataCHSW$THPostTot)
length(THDataCHSW$THPostTot)
propPostCHSW<- table(THDataCHSW$THPostTot)/sum(table(THDataCHSW$THPostTot))
perPostCHSW <- propPostCHSW*100
perPostCHSW
## Remove 6 From Trophy Hunting Post
THDataCHSW$THPostTot[THDataCHSW$THPostTot == 6] <- 3
THDataCHSW$THPostTot <- as.numeric(THDataCHSW$THPostTot)
table(THDataCHSW$THPostTot)
mean(THDataCHSW$THPostTot, na.rm = TRUE)
sd(THDataCHSW$THPostTot, na.rm = TRUE)
median(THDataCHSW$THPostTot, na.rm = TRUE)

###Difference
t.test(THDataCHSW$THPreTot, THDataCHSW$THPostTot, paired = TRUE)
##Effect Size
library(effsize)
cohen.d(THDataCHSW$THPreTot, THDataCHSW$THPostTot, paired = TRUE,
        within = FALSE)

###Test Difference Using Wilcoxon Signed Rank Test
wilcox.test(THDataCHSW$THPreTot, THDataCHSW$THPostTot, paired = TRUE)

###Effect Size Rank Biserial Correlation
library(effectsize)
rank_biserial(THDataCHSW$THPreTot, THDataCHSW$THPostTot, paired = TRUE,
              ci = 0.95)

##Message 6: Value Wildlife
#Remove Other Versions
THDataCHVW<- subset(THDataConH, Version != 1)
THDataCHVW<- subset(THDataCHVW, Version != 2)
THDataCHVW<- subset(THDataCHVW, Version != 3)
THDataCHVW<- subset(THDataCHVW, Version != 4)
THDataCHVW<- subset(THDataCHVW, Version != 5)
THDataCHVW<- subset(THDataCHVW, Version != 7)
THDataCHVW<- subset(THDataCHVW, Version != 8)
THDataCHVW<- subset(THDataCHVW, Version != 9)
```

```
table(THDataCHVW$Version)

#Get Pre and Post Values
###Approval Pre Message Summary
table(THDataCHVW$THPreTot)
length(THDataCHVW$THPreTot)
propPreCHVW<- table(THDataCHVW$THPreTot)/sum(table(THDataCHVW$THPreTot))
perPreCHVW <- propPreCHVW*100
perPreCHVW
## Remove 6 From Trophy Hunting Pre
THDataCHVW$THPreTot[THDataCHVW$THPreTot == 6] <- 3
THDataCHVW$THPreTot <- as.numeric(THDataCHVW$THPreTot)
table(THDataCHVW$THPreTot)
mean(THDataCHVW$THPreTot, na.rm = TRUE)
sd(THDataCHVW$THPreTot, na.rm = TRUE)
median(THDataCHVW$THPreTot, na.rm = TRUE)

###Approval Post Message Summary
table(THDataCHVW$THPostTot)
length(THDataCHVW$THPostTot)
propPostCHVW<- table(THDataCHVW$THPostTot)/sum(table(THDataCHVW$THPostTot))
perPostCHVW <- propPostCHVW*100
perPostCHVW
## Remove 6 From Trophy Hunting Post
THDataCHVW$THPostTot[THDataCHVW$THPostTot == 6] <- 3
THDataCHVW$THPostTot <- as.numeric(THDataCHVW$THPostTot)
table(THDataCHVW$THPostTot)
mean(THDataCHVW$THPostTot, na.rm = TRUE)
sd(THDataCHVW$THPostTot, na.rm = TRUE)
median(THDataCHVW$THPostTot, na.rm = TRUE)

###Difference
t.test(THDataCHVW$THPreTot, THDataCHVW$THPostTot, paired = TRUE)
##Effect Size
library(effsize)
cohen.d(THDataCHVW$THPreTot, THDataCHVW$THPostTot, paired = TRUE,
        within = FALSE)

###Test Difference Using Wilcoxon Signed Rank Test
wilcox.test(THDataCHVW$THPreTot, THDataCHVW$THPostTot, paired = TRUE)

###Effect Size Rank Biserial Correlation
library(effectsize)
rank_biserial(THDataCHVW$THPreTot, THDataCHVW$THPostTot, paired = TRUE,
              ci = 0.95)

##Message 7: Control Socioeconomic
#Remove Other Versions
THDataCHCS<- subset(THDataConH, Version != 1)
THDataCHCS<- subset(THDataCHCS, Version != 2)
THDataCHCS<- subset(THDataCHCS, Version != 3)
THDataCHCS<- subset(THDataCHCS, Version != 4)
THDataCHCS<- subset(THDataCHCS, Version != 5)
THDataCHCS<- subset(THDataCHCS, Version != 6)
THDataCHCS<- subset(THDataCHCS, Version != 8)
THDataCHCS<- subset(THDataCHCS, Version != 9)
table(THDataCHCS$Version)

#Get Pre and Post Values
###Approval Pre Message Summary
table(THDataCHCS$THPreTot)
length(THDataCHCS$THPreTot)
propPreCHCS<- table(THDataCHCS$THPreTot)/sum(table(THDataCHCS$THPreTot))
perPreCHCS <- propPreCHCS*100
perPreCHCS
```

```
## Remove 6 From Trophy Hunting Pre
THDataCHCS$THPreTot[THDataCHCS$THPreTot == 6] <- 3
THDataCHCS$THPreTot <- as.numeric(THDataCHCS$THPreTot)
table(THDataCHCS$THPreTot)
mean(THDataCHCS$THPreTot, na.rm = TRUE)
sd(THDataCHCS$THPreTot, na.rm = TRUE)
median(THDataCHCS$THPreTot, na.rm = TRUE)

###Approval Post Message Summary
table(THDataCHCS$THPostTot)
length(THDataCHCS$THPostTot)
propPostCHCS<- table(THDataCHCS$THPostTot)/sum(table(THDataCHCS$THPostTot))
perPostCHCS <- propPostCHCS*100
perPostCHCS
## Remove 6 From Trophy Hunting Post
THDataCHCS$THPostTot[THDataCHCS$THPostTot == 6] <- 3
THDataCHCS$THPostTot <- as.numeric(THDataCHCS$THPostTot)
table(THDataCHCS$THPostTot)
mean(THDataCHCS$THPostTot, na.rm = TRUE)
sd(THDataCHCS$THPostTot, na.rm = TRUE)
median(THDataCHCS$THPostTot, na.rm = TRUE)

###Difference
t.test(THDataCHCS$THPreTot, THDataCHCS$THPostTot, paired = TRUE)
##Effect Size
library(effsize)
cohen.d(THDataCHCS$THPreTot, THDataCHCS$THPostTot, paired = TRUE,
        within = FALSE)

###Test Difference Using Wilcoxon Signed Rank Test
wilcox.test(THDataCHCS$THPreTot, THDataCHCS$THPostTot, paired = TRUE)

###Effect Size Rank Biserial Correlation
library(effectsize)
rank_biserial(THDataCHCS$THPreTot, THDataCHCS$THPostTot, paired = TRUE,
              ci = 0.95)

##Message 8: Social Identity Socioeconomic
#Remove Other Versions
THDataCHSS<- subset(THDataConH, Version != 1)
THDataCHSS<- subset(THDataCHSS, Version != 2)
THDataCHSS<- subset(THDataCHSS, Version != 3)
THDataCHSS<- subset(THDataCHSS, Version != 4)
THDataCHSS<- subset(THDataCHSS, Version != 5)
THDataCHSS<- subset(THDataCHSS, Version != 6)
THDataCHSS<- subset(THDataCHSS, Version != 7)
THDataCHSS<- subset(THDataCHSS, Version != 9)
table(THDataCHSS$Version)

#Get Pre and Post Values
###Approval Pre Message Summary
table(THDataCHSS$THPreTot)
length(THDataCHSS$THPreTot)
propPreCHSS<- table(THDataCHSS$THPreTot)/sum(table(THDataCHSS$THPreTot))
perPreCHSS <- propPreCHSS*100
perPreCHSS
## Remove 6 From Trophy Hunting Pre
THDataCHSS$THPreTot[THDataCHSS$THPreTot == 6] <- 3
THDataCHSS$THPreTot <- as.numeric(THDataCHSS$THPreTot)
table(THDataCHSS$THPreTot)
mean(THDataCHSS$THPreTot, na.rm = TRUE)
sd(THDataCHSS$THPreTot, na.rm = TRUE)
median(THDataCHSS$THPreTot, na.rm = TRUE)

###Approval Post Message Summary
```

```
table(THDataCHSS$THPostTot)
length(THDataCHSS$THPostTot)
propPostCHSS<- table(THDataCHSS$THPostTot)/sum(table(THDataCHSS$THPostTot))
perPostCHSS <- propPostCHSS*100
perPostCHSS
## Remove 6 From Trophy Hunting Post
THDataCHSS$THPostTot[THDataCHSS$THPostTot == 6] <- 3
THDataCHSS$THPostTot <- as.numeric(THDataCHSS$THPostTot)
table(THDataCHSS$THPostTot)
mean(THDataCHSS$THPostTot, na.rm = TRUE)
sd(THDataCHSS$THPostTot, na.rm = TRUE)
median(THDataCHSS$THPostTot, na.rm = TRUE)

###Difference
t.test(THDataCHSS$THPreTot, THDataCHSS$THPostTot, paired = TRUE)

##Effect Size
library(effsize)
cohen.d(THDataCHSS$THPreTot, THDataCHSS$THPostTot, paired = TRUE,
        within = FALSE)

###Test Difference Using Wilcoxon Signed Rank Test
wilcox.test(THDataCHSS$THPreTot, THDataCHSS$THPostTot, paired = TRUE)

###Effect Size Rank Biserial Correlation
library(effectsize)
rank_biserial(THDataCHSS$THPreTot, THDataCHSS$THPostTot, paired = TRUE,
              ci = 0.95)

##Message 9: Value Socioeconomic
#Remove Other Versions
THDataCHVS<- subset(THDataConH, Version != 1)
THDataCHVS<- subset(THDataCHVS, Version != 2)
THDataCHVS<- subset(THDataCHVS, Version != 3)
THDataCHVS<- subset(THDataCHVS, Version != 4)
THDataCHVS<- subset(THDataCHVS, Version != 5)
THDataCHVS<- subset(THDataCHVS, Version != 6)
THDataCHVS<- subset(THDataCHVS, Version != 7)
THDataCHVS<- subset(THDataCHVS, Version != 8)
table(THDataCHVS$Version)

#Get Pre and Post Values
###Approval Pre Message Summary
table(THDataCHVS$THPreTot)
length(THDataCHVS$THPreTot)
propPreCHVS<- table(THDataCHVS$THPreTot)/sum(table(THDataCHVS$THPreTot))
perPreCHVS <- propPreCHVS*100
perPreCHVS
## Remove 6 From Trophy Hunting Pre
THDataCHVS$THPreTot[THDataCHVS$THPreTot == 6] <- 3
THDataCHVS$THPreTot <- as.numeric(THDataCHVS$THPreTot)
table(THDataCHVS$THPreTot)
mean(THDataCHVS$THPreTot, na.rm = TRUE)
sd(THDataCHVS$THPreTot, na.rm = TRUE)
median(THDataCHVS$THPreTot, na.rm = TRUE)

###Approval Post Message Summary
table(THDataCHVS$THPostTot)
length(THDataCHVS$THPostTot)
propPostCHVS<- table(THDataCHVS$THPostTot)/sum(table(THDataCHVS$THPostTot))
perPostCHVS <- propPostCHVS*100
perPostCHVS
## Remove 6 From Trophy Hunting Post
THDataCHVS$THPostTot[THDataCHVS$THPostTot == 6] <- 3
THDataCHVS$THPostTot <- as.numeric(THDataCHVS$THPostTot)
```

```
table(THDataCHVS$THPostTot)
mean(THDataCHVS$THPostTot, na.rm = TRUE)
sd(THDataCHVS$THPostTot, na.rm = TRUE)
median(THDataCHVS$THPostTot, na.rm = TRUE)

###Difference
t.test(THDataCHVS$THPreTot, THDataCHVS$THPostTot, paired = TRUE)
##Effect Size
library(effsize)
cohen.d(THDataCHVS$THPreTot, THDataCHVS$THPostTot, paired = TRUE,
        within = FALSE)

###Test Difference Using Wilcoxon Signed Rank Test
wilcox.test(THDataCHVS$THPreTot, THDataCHVS$THPostTot, paired = TRUE)

###Effect Size Rank Biserial Correlation
library(effectsize)
rank_biserial(THDataCHVS$THPreTot, THDataCHVS$THPostTot, paired = TRUE,
              ci = 0.95)

##Economic and Financial Success
THData$ValueEconomicTot <- as.numeric(THData$ValueEconomicTot)
Ecofin <- THData$ValueEconomicTot
Ecofin
table(Ecofin)
length(Ecofin)

###Create High and Low Extremes
#High
THData$ValueEconomicTot[THData$ValueEconomicTot == 2] <- 1
#Low
THData$ValueEconomicTot[THData$ValueEconomicTot == 4] <- 2
THData$ValueEconomicTot[THData$ValueEconomicTot == 5] <- 2
#Exclude Middle
THData$ValueEconomicTot[THData$ValueEconomicTot == 3] <- NA
table(THData$ValueEconomicTot)


#####Economic and Financial Extremes
###Create Economic High Rank Only Sample
THDataEcoH<- subset(THData, ValueEconomicTot != 2)
table(THDataEcoH$ValueEconomicTot)

########Difference in TH Attitudes Pre and Post Message

######Pre Message
###Approval Pre Message Summary
table(THDataEcoH$THPreTot)
length(THDataEcoH$THPreTot)
propPreAllEcoH<- table(THDataEcoH$THPreTot)/sum(table(THDataEcoH$THPreTot))
perPreAllEcoH <- propPreAllEcoH*100
perPreAllEcoH
THDataEcoH$THPreTot <- as.numeric(THDataEcoH$THPreTot)

#Get TH Approval by Message
table(THDataEcoH$THPreTot, THDataEcoH$Version)

## Remove 6 From Trophy Hunting Pre
THDataEcoH$THPreTot[THDataEcoH$THPreTot == 6] <- 3
table(THDataEcoH$THPreTot)
#Get TH Approval by Message
table(THDataEcoH$THPreTot, THDataEcoH$Version)
aggregate(THPreTot ~ Version, data = THDataEcoH, FUN = mean)
aggregate(THPreTot ~ Version, data = THDataEcoH, FUN = sd)
aggregate(THPreTot ~ Version, data = THDataEcoH, FUN = median)
```

```
mean(THDataEcoH$THPreTot, na.rm = TRUE)
sd(THDataEcoH$THPreTot, na.rm = TRUE)
median(THDataEcoH$THPreTot, na.rm = TRUE)

##Determine Differences by Message Group
##ANOVA Model
EcoHPreMesMod <- aov(THPreTot ~ Version, data = THDataEcoH)
summary(EcoHPreMesMod)

library(effectsize)
eta_squared(EcoHPreMesMod, partial = FALSE)

## Check normality of the residuals with the Shapiro-Wilk test
shapiro.test(EcoHPreMesMod$residuals)
###Check for equality of variance
library(car)
leveneTest(THPreTot ~Version,
           data = THDataEcoH)

##Kruskal Wallis Test
library(rstatix)
kruskal.test(THPreTot ~ Version, data = THDataEcoH)

#####Post Message
###Approval Post Message Summary
table(THDataEcoH$THPostTot)
length(THDataEcoH$THPostTot)
propPostAllEcoH<- table(THDataEcoH$THPostTot)/sum(table(THDataEcoH$THPostTot))
perPostAllEcoH <- propPostAllEcoH*100
perPostAllEcoH
THDataEcoH$THPostTot <- as.numeric(THDataEcoH$THPostTot)
#Get TH Approval by Message
table(THDataEcoH$THPostTot, THDataEcoH$Version)

## Remove 6 From Trophy Hunting Post
THDataEcoH$THPostTot[THDataEcoH$THPostTot == 6] <- 3
table(THDataEcoH$THPostTot)
#Get TH Approval by Message
table(THDataEcoH$THPostTot, THDataEcoH$Version)
aggregate(THPostTot ~ Version, data = THDataEcoH, FUN = mean)
aggregate(THPostTot ~ Version, data = THDataEcoH, FUN = sd)
aggregate(THPostTot ~ Version, data = THDataEcoH, FUN = median)
mean(THDataEcoH$THPostTot, na.rm = TRUE)
sd(THDataEcoH$THPostTot, na.rm = TRUE)
median(THDataEcoH$THPostTot, na.rm = TRUE)

##Determine Differences by Message Group
##ANOVA Model
EcoHPostMesMod <- aov(THPostTot ~ Version, data = THDataEcoH)
summary(EcoHPostMesMod)

library(effectsize)
eta_squared(EcoHPostMesMod, partial = FALSE)

##Bonferroni
pairwise.t.test(THDataEcoH$THPostTot, THDataEcoH$Version, p.adj = "bonf")

##Make table with Bonf significance
EcoHAllPostApp <- aggregate(THPostTot ~ Version, data = THDataEcoH, FUN = mean)
EcoHAllPostApp
#define new column
BonSigAllPostEcoH <- c('ab','ab','a', 'ab', 'ab', 'b', 'ab', 'ab', 'ab')
#add column
EcoHAllPostSig <- cbind(EcoHAllPostApp, BonSigAllPostEcoH)
EcoHAllPostSig
```

```
## Check normality of the residuals with the Shapiro-Wilk test
shapiro.test(EcoHPostMesMod$residuals)
###Check for equality of variance
library(car)
leveneTest(THPostTot ~Version,
           data = THDataEcoH)

##Kruskal Wallis Test
library(rstatix)
kruskal.test(THPostTot ~ Version, data = THDataEcoH)

# pairwise using dunn test
DunnEcoHPost <- dunn_test(THPostTot ~ Version, data = THDataEcoH,
                          p.adjust = "bonferroni")
DunnEcoHPost
print(DunnEcoHPost, n = 36)

##Make table with Bonf significance
NPEcoHAllPostApp <- aggregate(THPostTot ~ Version, data = THDataEcoH,
                              FUN = median)
NPEcoHAllPostApp
#define new column to add
NPBonSigAllPostEcoH <- c('ab','ab','a', 'ab', 'ab', 'b', 'ab', 'ab', 'ab')
#add column called 'new'
NPEcoHAllPostSig <- cbind(NPEcoHAllPostApp, NPBonSigAllPostEcoH)
#view new data frame
NPEcoHAllPostSig

##Separate by Message
table(THDataEcoH$Version)

##Message 1: Control Control
#Remove Other Versions
THDataEHCC<- subset(THDataEcoH, Version != 2)
THDataEHCC<- subset(THDataEHCC, Version != 3)
THDataEHCC<- subset(THDataEHCC, Version != 4)
THDataEHCC<- subset(THDataEHCC, Version != 5)
THDataEHCC<- subset(THDataEHCC, Version != 6)
THDataEHCC<- subset(THDataEHCC, Version != 7)
THDataEHCC<- subset(THDataEHCC, Version != 8)
THDataEHCC<- subset(THDataEHCC, Version != 9)
table(THDataEHCC$Version)

#Get Pre and Post Values
###Approval Pre Message Summary
table(THDataEHCC$THPreTot)
length(THDataEHCC$THPreTot)
propPreEHCC<- table(THDataEHCC$THPreTot)/sum(table(THDataEHCC$THPreTot))
perPreEHCC <- propPreEHCC*100
perPreEHCC
## Remove 6 From Trophy Hunting Pre
THDataEHCC$THPreTot[THDataEHCC$THPreTot == 6] <- 3
THDataEHCC$THPreTot <- as.numeric(THDataEHCC$THPreTot)
table(THDataEHCC$THPreTot)
length(THDataEHCC$THPreTot)
mean(THDataEHCC$THPreTot, na.rm = TRUE)
sd(THDataEHCC$THPreTot, na.rm = TRUE)
median(THDataEHCC$THPreTot, na.rm = TRUE)

###Approval Post Message Summary
table(THDataEHCC$THPostTot)
length(THDataEHCC$THPostTot)
propPostEHCC<- table(THDataEHCC$THPostTot)/sum(table(THDataEHCC$THPostTot))
perPostEHCC <- propPostEHCC*100
```

```
perPostEHCC
## Remove 6 From Trophy Hunting Post
THDataEHCC$THPostTot[THDataEHCC$THPostTot == 6] <- 3
THDataEHCC$THPostTot <- as.numeric(THDataEHCC$THPostTot)
table(THDataEHCC$THPostTot)
mean(THDataEHCC$THPostTot, na.rm = TRUE)
sd(THDataEHCC$THPostTot, na.rm = TRUE)
median(THDataEHCC$THPostTot, na.rm = TRUE)

###Difference
t.test(THDataEHCC$THPreTot, THDataEHCC$THPostTot, paired = TRUE)
##Effect Size
library(effsize)
cohen.d(THDataEHCC$THPreTot, THDataEHCC$THPostTot, paired = TRUE,
        within = FALSE)

###Test Difference Using Wilcoxon Signed Rank Test
wilcox.test(THDataEHCC$THPreTot, THDataEHCC$THPostTot, paired = TRUE)

###Effect Size Rank Biserial Correlation
library(effectsize)
rank_biserial(THDataEHCC$THPreTot, THDataEHCC$THPostTot, paired = TRUE,
              ci = 0.95)

##Message 2: Social Identity Control
#Remove Other Versions
THDataEHSC<- subset(THDataEcoH, Version != 1)
THDataEHSC<- subset(THDataEHSC, Version != 3)
THDataEHSC<- subset(THDataEHSC, Version != 4)
THDataEHSC<- subset(THDataEHSC, Version != 5)
THDataEHSC<- subset(THDataEHSC, Version != 6)
THDataEHSC<- subset(THDataEHSC, Version != 7)
THDataEHSC<- subset(THDataEHSC, Version != 8)
THDataEHSC<- subset(THDataEHSC, Version != 9)
table(THDataEHSC$Version)

#Get Pre and Post Values
###Approval Pre Message Summary
table(THDataEHSC$THPreTot)
length(THDataEHSC$THPreTot)
propPreEHSC<- table(THDataEHSC$THPreTot)/sum(table(THDataEHSC$THPreTot))
perPreEHSC <- propPreEHSC*100
perPreEHSC
## Remove 6 From Trophy Hunting Pre
THDataEHSC$THPreTot[THDataEHSC$THPreTot == 6] <- 3
THDataEHSC$THPreTot <- as.numeric(THDataEHSC$THPreTot)
table(THDataEHSC$THPreTot)
mean(THDataEHSC$THPreTot, na.rm = TRUE)
sd(THDataEHSC$THPreTot, na.rm = TRUE)
median(THDataEHSC$THPreTot, na.rm = TRUE)

###Approval Post Message Summary
table(THDataEHSC$THPostTot)
length(THDataEHSC$THPostTot)
propPostEHSC<- table(THDataEHSC$THPostTot)/sum(table(THDataEHSC$THPostTot))
perPostEHSC <- propPostEHSC*100
perPostEHSC
## Remove 6 From Trophy Hunting Post
THDataEHSC$THPostTot[THDataEHSC$THPostTot == 6] <- 3
THDataEHSC$THPostTot <- as.numeric(THDataEHSC$THPostTot)
table(THDataEHSC$THPostTot)
mean(THDataEHSC$THPostTot, na.rm = TRUE)
sd(THDataEHSC$THPostTot, na.rm = TRUE)
median(THDataEHSC$THPostTot, na.rm = TRUE)
```

```
###Difference
t.test(THDataEHSC$THPreTot, THDataEHSC$THPostTot, paired = TRUE)
##Effect Size
library(effsize)
cohen.d(THDataEHSC$THPreTot, THDataEHSC$THPostTot, paired = TRUE,
        within = FALSE)

###Test Difference Using Wilcoxon Signed Rank Test
wilcox.test(THDataEHSC$THPreTot, THDataEHSC$THPostTot, paired = TRUE)

###Effect Size Rank Biserial Correlation
library(effectsize)
rank_biserial(THDataEHSC$THPreTot, THDataEHSC$THPostTot, paired = TRUE,
              ci = 0.95)

##Message 3: Value Control
#Remove Other Versions
THDataEHVC<- subset(THDataEcoH, Version != 1)
THDataEHVC<- subset(THDataEHVC, Version != 2)
THDataEHVC<- subset(THDataEHVC, Version != 4)
THDataEHVC<- subset(THDataEHVC, Version != 5)
THDataEHVC<- subset(THDataEHVC, Version != 6)
THDataEHVC<- subset(THDataEHVC, Version != 7)
THDataEHVC<- subset(THDataEHVC, Version != 8)
THDataEHVC<- subset(THDataEHVC, Version != 9)
table(THDataEHVC$Version)

#Get Pre and Post Values
###Approval Pre Message Summary
table(THDataEHVC$THPreTot)
length(THDataEHVC$THPreTot)
propPreEHVC<- table(THDataEHVC$THPreTot)/sum(table(THDataEHVC$THPreTot))
perPreEHVC <- propPreEHVC*100
perPreEHVC
## Remove 6 From Trophy Hunting Pre
THDataEHVC$THPreTot[THDataEHVC$THPreTot == 6] <- 3
THDataEHVC$THPreTot <- as.numeric(THDataEHVC$THPreTot)
table(THDataEHVC$THPreTot)
mean(THDataEHVC$THPreTot, na.rm = TRUE)
sd(THDataEHVC$THPreTot, na.rm = TRUE)
median(THDataEHVC$THPreTot, na.rm = TRUE)

###Approval Post Message Summary
table(THDataEHVC$THPostTot)
length(THDataEHVC$THPostTot)
propPostEHVC<- table(THDataEHVC$THPostTot)/sum(table(THDataEHVC$THPostTot))
perPostEHVC <- propPostEHVC*100
perPostEHVC
## Remove 6 From Trophy Hunting Post
THDataEHVC$THPostTot[THDataEHVC$THPostTot == 6] <- 3
THDataEHVC$THPostTot <- as.numeric(THDataEHVC$THPostTot)
table(THDataEHVC$THPostTot)
mean(THDataEHVC$THPostTot, na.rm = TRUE)
sd(THDataEHVC$THPostTot, na.rm = TRUE)
median(THDataEHVC$THPostTot, na.rm = TRUE)

###Difference
t.test(THDataEHVC$THPreTot, THDataEHVC$THPostTot, paired = TRUE)
##Effect Size
library(effsize)
cohen.d(THDataEHVC$THPreTot, THDataEHVC$THPostTot, paired = TRUE,
        within = FALSE)

###Test Difference Using Wilcoxon Signed Rank Test
wilcox.test(THDataEHVC$THPreTot, THDataEHVC$THPostTot, paired = TRUE)
```

```
###Effect Size Rank Biserial Correlation
library(effectsize)
rank_biserial(THDataEHVC$THPreTot, THDataEHVC$THPostTot, paired = TRUE,
              ci = 0.95)

##Message 4: Control Wildlife
#Remove Other Versions
THDataEHCW<- subset(THDataEcoH, Version != 1)
THDataEHCW<- subset(THDataEHCW, Version != 2)
THDataEHCW<- subset(THDataEHCW, Version != 3)
THDataEHCW<- subset(THDataEHCW, Version != 5)
THDataEHCW<- subset(THDataEHCW, Version != 6)
THDataEHCW<- subset(THDataEHCW, Version != 7)
THDataEHCW<- subset(THDataEHCW, Version != 8)
THDataEHCW<- subset(THDataEHCW, Version != 9)
table(THDataEHCW$Version)

#Get Pre and Post Values
###Approval Pre Message Summary
table(THDataEHCW$THPreTot)
length(THDataEHCW$THPreTot)
propPreEHCW<- table(THDataEHCW$THPreTot)/sum(table(THDataEHCW$THPreTot))
perPreEHCW <- propPreEHCW*100
perPreEHCW
## Remove 6 From Trophy Hunting Pre
THDataEHCW$THPreTot[THDataEHCW$THPreTot == 6] <- 3
THDataEHCW$THPreTot <- as.numeric(THDataEHCW$THPreTot)
table(THDataEHCW$THPreTot)
mean(THDataEHCW$THPreTot, na.rm = TRUE)
sd(THDataEHCW$THPreTot, na.rm = TRUE)
median(THDataEHCW$THPreTot, na.rm = TRUE)

###Approval Post Message Summary
table(THDataEHCW$THPostTot)
length(THDataEHCW$THPostTot)
propPostEHCW<- table(THDataEHCW$THPostTot)/sum(table(THDataEHCW$THPostTot))
perPostEHCW <- propPostEHCW*100
perPostEHCW
## Remove 6 From Trophy Hunting Post
THDataEHCW$THPostTot[THDataEHCW$THPostTot == 6] <- 3
THDataEHCW$THPostTot <- as.numeric(THDataEHCW$THPostTot)
table(THDataEHCW$THPostTot)
mean(THDataEHCW$THPostTot, na.rm = TRUE)
sd(THDataEHCW$THPostTot, na.rm = TRUE)
median(THDataEHCW$THPostTot, na.rm = TRUE)

###Difference
t.test(THDataEHCW$THPreTot, THDataEHCW$THPostTot, paired = TRUE)
##Effect Size
library(effsize)
cohen.d(THDataEHCW$THPreTot, THDataEHCW$THPostTot, paired = TRUE,
        within = FALSE)

###Test Difference Using Wilcoxon Signed Rank Test
wilcox.test(THDataEHCW$THPreTot, THDataEHCW$THPostTot, paired = TRUE)

###Effect Size Rank Biserial Correlation
library(effectsize)
rank_biserial(THDataEHCW$THPreTot, THDataEHCW$THPostTot, paired = TRUE,
              ci = 0.95)

##Message 5: Social Identity Wildlife
#Remove Other Versions
THDataEHSW<- subset(THDataEcoH, Version != 1)
```

```
THDataEHSW<- subset(THDataEHSW, Version != 2)
THDataEHSW<- subset(THDataEHSW, Version != 3)
THDataEHSW<- subset(THDataEHSW, Version != 4)
THDataEHSW<- subset(THDataEHSW, Version != 6)
THDataEHSW<- subset(THDataEHSW, Version != 7)
THDataEHSW<- subset(THDataEHSW, Version != 8)
THDataEHSW<- subset(THDataEHSW, Version != 9)
table(THDataEHSW$Version)

#Get Pre and Post Values
###Approval Pre Message Summary
table(THDataEHSW$THPreTot)
length(THDataEHSW$THPreTot)
propPreEHSW<- table(THDataEHSW$THPreTot)/sum(table(THDataEHSW$THPreTot))
perPreEHSW <- propPreEHSW*100
perPreEHSW
## Remove 6 From Trophy Hunting Pre
THDataEHSW$THPreTot[THDataEHSW$THPreTot == 6] <- 3
THDataEHSW$THPreTot <- as.numeric(THDataEHSW$THPreTot)
table(THDataEHSW$THPreTot)
mean(THDataEHSW$THPreTot, na.rm = TRUE)
sd(THDataEHSW$THPreTot, na.rm = TRUE)
median(THDataEHSW$THPreTot, na.rm = TRUE)

###Approval Post Message Summary
table(THDataEHSW$THPostTot)
length(THDataEHSW$THPostTot)
propPostEHSW<- table(THDataEHSW$THPostTot)/sum(table(THDataEHSW$THPostTot))
perPostEHSW <- propPostEHSW*100
perPostEHSW
## Remove 6 From Trophy Hunting Post
THDataEHSW$THPostTot[THDataEHSW$THPostTot == 6] <- 3
THDataEHSW$THPostTot <- as.numeric(THDataEHSW$THPostTot)
table(THDataEHSW$THPostTot)
mean(THDataEHSW$THPostTot, na.rm = TRUE)
sd(THDataEHSW$THPostTot, na.rm = TRUE)
median(THDataEHSW$THPostTot, na.rm = TRUE)

###Difference
t.test(THDataEHSW$THPreTot, THDataEHSW$THPostTot, paired = TRUE)
##Effect Size
library(effsize)
cohen.d(THDataEHSW$THPreTot, THDataEHSW$THPostTot, paired = TRUE,
        within = FALSE)

###Test Difference Using Wilcoxon Signed Rank Test
wilcox.test(THDataEHSW$THPreTot, THDataEHSW$THPostTot, paired = TRUE)

###Effect Size Rank Biserial Correlation
library(effectsize)
rank_biserial(THDataEHSW$THPreTot, THDataEHSW$THPostTot, paired = TRUE,
              ci = 0.95)

##Message 6: Value Wildlife
#Remove Other Versions
THDataEHVW<- subset(THDataEcoH, Version != 1)
THDataEHVW<- subset(THDataEHVW, Version != 2)
THDataEHVW<- subset(THDataEHVW, Version != 3)
THDataEHVW<- subset(THDataEHVW, Version != 4)
THDataEHVW<- subset(THDataEHVW, Version != 5)
THDataEHVW<- subset(THDataEHVW, Version != 7)
THDataEHVW<- subset(THDataEHVW, Version != 8)
THDataEHVW<- subset(THDataEHVW, Version != 9)
table(THDataEHVW$Version)
```

```
#Get Pre and Post Values
###Approval Pre Message Summary
table(THDataEHVW$THPreTot)
length(THDataEHVW$THPreTot)
propPreEHVW<- table(THDataEHVW$THPreTot)/sum(table(THDataEHVW$THPreTot))
perPreEHVW <- propPreEHVW*100
perPreEHVW
## Remove 6 From Trophy Hunting Pre
THDataEHVW$THPreTot[THDataEHVW$THPreTot == 6] <- 3
THDataEHVW$THPreTot <- as.numeric(THDataEHVW$THPreTot)
table(THDataEHVW$THPreTot)
mean(THDataEHVW$THPreTot, na.rm = TRUE)
sd(THDataEHVW$THPreTot, na.rm = TRUE)
median(THDataEHVW$THPreTot, na.rm = TRUE)

###Approval Post Message Summary
table(THDataEHVW$THPostTot)
length(THDataEHVW$THPostTot)
propPostEHVW<- table(THDataEHVW$THPostTot)/sum(table(THDataEHVW$THPostTot))
perPostEHVW <- propPostEHVW*100
perPostEHVW
## Remove 6 From Trophy Hunting Post
THDataEHVW$THPostTot[THDataEHVW$THPostTot == 6] <- 3
THDataEHVW$THPostTot <- as.numeric(THDataEHVW$THPostTot)
table(THDataEHVW$THPostTot)
mean(THDataEHVW$THPostTot, na.rm = TRUE)
sd(THDataEHVW$THPostTot, na.rm = TRUE)
median(THDataEHVW$THPostTot, na.rm = TRUE)

###Difference
t.test(THDataEHVW$THPreTot, THDataEHVW$THPostTot, paired = TRUE)
##Effect Size
library(effsize)
cohen.d(THDataEHVW$THPreTot, THDataEHVW$THPostTot, paired = TRUE,
        within = FALSE)

###Test Difference Using Wilcoxon Signed Rank Test
wilcox.test(THDataEHVW$THPreTot, THDataEHVW$THPostTot, paired = TRUE)

###Effect Size Rank Biserial Correlation
library(effectsize)
rank_biserial(THDataEHVW$THPreTot, THDataEHVW$THPostTot, paired = TRUE,
              ci = 0.95)

##Message 7: Control Socioeconomic
#Remove Other Versions
THDataEHCS<- subset(THDataEcoH, Version != 1)
THDataEHCS<- subset(THDataEHCS, Version != 2)
THDataEHCS<- subset(THDataEHCS, Version != 3)
THDataEHCS<- subset(THDataEHCS, Version != 4)
THDataEHCS<- subset(THDataEHCS, Version != 5)
THDataEHCS<- subset(THDataEHCS, Version != 6)
THDataEHCS<- subset(THDataEHCS, Version != 8)
THDataEHCS<- subset(THDataEHCS, Version != 9)
table(THDataEHCS$Version)

#Get Pre and Post Values
###Approval Pre Message Summary
table(THDataEHCS$THPreTot)
length(THDataEHCS$THPreTot)
propPreEHCS<- table(THDataEHCS$THPreTot)/sum(table(THDataEHCS$THPreTot))
perPreEHCS <- propPreEHCS*100
perPreEHCS
## Remove 6 From Trophy Hunting Pre
THDataEHCS$THPreTot[THDataEHCS$THPreTot == 6] <- 3
```

```
THDataEHCS$THPreTot <- as.numeric(THDataEHCS$THPreTot)
table(THDataEHCS$THPreTot)
mean(THDataEHCS$THPreTot, na.rm = TRUE)
sd(THDataEHCS$THPreTot, na.rm = TRUE)
median(THDataEHCS$THPreTot, na.rm = TRUE)

###Approval Post Message Summary
table(THDataEHCS$THPostTot)
length(THDataEHCS$THPostTot)
propPostEHCS<- table(THDataEHCS$THPostTot)/sum(table(THDataEHCS$THPostTot))
perPostEHCS <- propPostEHCS*100
perPostEHCS
## Remove 6 From Trophy Hunting Post
THDataEHCS$THPostTot[THDataEHCS$THPostTot == 6] <- 3
THDataEHCS$THPostTot <- as.numeric(THDataEHCS$THPostTot)
table(THDataEHCS$THPostTot)
mean(THDataEHCS$THPostTot, na.rm = TRUE)
sd(THDataEHCS$THPostTot, na.rm = TRUE)
median(THDataEHCS$THPostTot, na.rm = TRUE)

###Difference
t.test(THDataEHCS$THPreTot, THDataEHCS$THPostTot, paired = TRUE)
##Effect Size
library(effsize)
cohen.d(THDataEHCS$THPreTot, THDataEHCS$THPostTot, paired = TRUE,
        within = FALSE)

###Test Difference Using Wilcoxon Signed Rank Test
wilcox.test(THDataEHCS$THPreTot, THDataEHCS$THPostTot, paired = TRUE)

###Effect Size Rank Biserial Correlation
library(effectsize)
rank_biserial(THDataEHCS$THPreTot, THDataEHCS$THPostTot, paired = TRUE,
              ci = 0.95)

##Message 8: Social Identity Socioeconomic
#Remove Other Versions
THDataEHSS<- subset(THDataEcoH, Version != 1)
THDataEHSS<- subset(THDataEHSS, Version != 2)
THDataEHSS<- subset(THDataEHSS, Version != 3)
THDataEHSS<- subset(THDataEHSS, Version != 4)
THDataEHSS<- subset(THDataEHSS, Version != 5)
THDataEHSS<- subset(THDataEHSS, Version != 6)
THDataEHSS<- subset(THDataEHSS, Version != 7)
THDataEHSS<- subset(THDataEHSS, Version != 9)
table(THDataEHSS$Version)

#Get Pre and Post Values
###Approval Pre Message Summary
table(THDataEHSS$THPreTot)
length(THDataEHSS$THPreTot)
propPreEHSS<- table(THDataEHSS$THPreTot)/sum(table(THDataEHSS$THPreTot))
perPreEHSS <- propPreEHSS*100
perPreEHSS
## Remove 6 From Trophy Hunting Pre
THDataEHSS$THPreTot[THDataEHSS$THPreTot == 6] <- 3
THDataEHSS$THPreTot <- as.numeric(THDataEHSS$THPreTot)
table(THDataEHSS$THPreTot)
mean(THDataEHSS$THPreTot, na.rm = TRUE)
sd(THDataEHSS$THPreTot, na.rm = TRUE)
median(THDataEHSS$THPreTot, na.rm = TRUE)

###Approval Post Message Summary
table(THDataEHSS$THPostTot)
length(THDataEHSS$THPostTot)
```

```
propPostEHSS<- table(THDataEHSS$THPostTot)/sum(table(THDataEHSS$THPostTot))
perPostEHSS <- propPostEHSS*100
perPostEHSS
## Remove 6 From Trophy Hunting Post
THDataEHSS$THPostTot[THDataEHSS$THPostTot == 6] <- 3
THDataEHSS$THPostTot <- as.numeric(THDataEHSS$THPostTot)
table(THDataEHSS$THPostTot)
mean(THDataEHSS$THPostTot, na.rm = TRUE)
sd(THDataEHSS$THPostTot, na.rm = TRUE)
median(THDataEHSS$THPostTot, na.rm = TRUE)

###Difference
t.test(THDataEHSS$THPreTot, THDataEHSS$THPostTot, paired = TRUE)
##Effect Size
library(effsize)
cohen.d(THDataEHSS$THPreTot, THDataEHSS$THPostTot, paired = TRUE,
        within = FALSE)

###Test Difference Using Wilcoxon Signed Rank Test
wilcox.test(THDataEHSS$THPreTot, THDataEHSS$THPostTot, paired = TRUE)

###Effect Size Rank Biserial Correlation
library(effectsize)
rank_biserial(THDataEHSS$THPreTot, THDataEHSS$THPostTot, paired = TRUE,
              ci = 0.95)

##Message 9: Value Socioeconomic
#Remove Other Versions
THDataEHVS<- subset(THDataEcoH, Version != 1)
THDataEHVS<- subset(THDataEHVS, Version != 2)
THDataEHVS<- subset(THDataEHVS, Version != 3)
THDataEHVS<- subset(THDataEHVS, Version != 4)
THDataEHVS<- subset(THDataEHVS, Version != 5)
THDataEHVS<- subset(THDataEHVS, Version != 6)
THDataEHVS<- subset(THDataEHVS, Version != 7)
THDataEHVS<- subset(THDataEHVS, Version != 8)
table(THDataEHVS$Version)

#Get Pre and Post Values
###Approval Pre Message Summary
table(THDataEHVS$THPreTot)
length(THDataEHVS$THPreTot)
propPreEHVS<- table(THDataEHVS$THPreTot)/sum(table(THDataEHVS$THPreTot))
perPreEHVS <- propPreEHVS*100
perPreEHVS
## Remove 6 From Trophy Hunting Pre
THDataEHVS$THPreTot[THDataEHVS$THPreTot == 6] <- 3
THDataEHVS$THPreTot <- as.numeric(THDataEHVS$THPreTot)
table(THDataEHVS$THPreTot)
mean(THDataEHVS$THPreTot, na.rm = TRUE)
sd(THDataEHVS$THPreTot, na.rm = TRUE)
median(THDataEHVS$THPreTot, na.rm = TRUE)

###Approval Post Message Summary
table(THDataEHVS$THPostTot)
length(THDataEHVS$THPostTot)
propPostEHVS<- table(THDataEHVS$THPostTot)/sum(table(THDataEHVS$THPostTot))
perPostEHVS <- propPostEHVS*100
perPostEHVS
## Remove 6 From Trophy Hunting Post
THDataEHVS$THPostTot[THDataEHVS$THPostTot == 6] <- 3
THDataEHVS$THPostTot <- as.numeric(THDataEHVS$THPostTot)
table(THDataEHVS$THPostTot)
mean(THDataEHVS$THPostTot, na.rm = TRUE)
sd(THDataEHVS$THPostTot, na.rm = TRUE)
```

```
median(THDataEHVS$THPostTot, na.rm = TRUE)

###Difference
t.test(THDataEHVS$THPreTot, THDataEHVS$THPostTot, paired = TRUE)
##Effect Size
library(effsize)
cohen.d(THDataEHVS$THPreTot, THDataEHVS$THPostTot, paired = TRUE,
        within = FALSE)

###Test Difference Using Wilcoxon Signed Rank Test
wilcox.test(THDataEHVS$THPreTot, THDataEHVS$THPostTot, paired = TRUE)

###Effect Size Rank Biserial Correlation
library(effectsize)
rank_biserial(THDataEHVS$THPreTot, THDataEHVS$THPostTot, paired = TRUE,
              ci = 0.95)

##########Message Source Trust
#Summaries by Source

#####USFWS
table(THData$SourceUSFWS)
length(THData$SourceUSFWS)
propUSFWS<- table(THData$SourceUSFWS)/sum(table(THData$SourceUSFWS))
perUSFWS <- propUSFWS*100
perUSFWS

#####SADEA
table(THData$SourceSADEA)
length(THData$SourceSADEA)
propSADEA<- table(THData$SourceSADEA)/sum(table(THData$SourceSADEA))
perSADEA <- propSADEA*100
perSADEA

#####SCI
table(THData$SourceSCI)
length(THData$SourceSCI)
propSCI<- table(THData$SourceSCI)/sum(table(THData$SourceSCI))
perSCI <- propSCI*100
perSCI

#####WWF
table(THData$SourceWWF)
length(THData$SourceWWF)
propWWF<- table(THData$SourceWWF)/sum(table(THData$SourceWWF))
perWWF <- propWWF*100
perWWF

#####TNC
table(THData$SourceTNC)
length(THData$SourceTNC)
propTNC<- table(THData$SourceTNC)/sum(table(THData$SourceTNC))
perTNC <- propTNC*100
perTNC

####Scientific Journal
table(THData$SourceJournal)
length(THData$SourceJournal)
propSJ<- table(THData$SourceJournal)/sum(table(THData$SourceJournal))
perSJ <- propSJ*100
perSJ

########Direct Comparison of Trust in Sources
#Data reformatted in excel by creating new sheet with 2 columns
#One for source ID, one for trust value
```

```
setwd("")
library(readxl)
THData1 <- read_xlsx("TH Data Sheet Labelled.xlsx", sheet = "Sheet8")
head(THData1)
THData1$Trust <- as.numeric(THData1$Trust)

###Get Trust by Source
table(THData1$Trust, THData1$Source)
## Remove 4 From Trust
THData1$Trust[THData1$Trust == 4] <- NA
table(THData1$Trust)
length(THData1$Trust)
table(THData1$Trust, THData1$Source)
aggregate(Trust ~ Source, data = THData1, FUN = mean)
aggregate(Trust ~ Source, data = THData1, FUN = sd)
aggregate(Trust ~ Source, data = THData1, FUN = median)

##ANOVA Model
TrustMod <- aov(Trust ~ Source, data = THData1)
summary(TrustMod)

library(effectsize)
eta_squared(TrustMod, partial = FALSE)

##Bonferroni
pairwise.t.test(THData1$Trust, THData1$Source, p.adj = "bonf")

##Make table with Bonf significance
TrustApp <- aggregate(Trust ~ Source, data = THData1, FUN = mean)
TrustApp
#define new column
BonSigTrust <- c('a','b','c', 'd', 'ad', 'd')
#add column
TrustSig <- cbind(TrustApp, BonSigTrust)
TrustSig

## Check normality of the residuals with the Shapiro-Wilk test
shapiro.test(TrustMod$residuals)
###Error
###Check for equality of variance
library(car)
leveneTest(Trust ~Source,
           data = THData1)

##Kruskal Wallis Test
library(rstatix)
kruskal.test(Trust ~ Source, data = THData1)

# pairwise using dunn test
dunn_test(Trust ~ Source, data = THData1,
          p.adjust = "bonferroni")

##Make table with Bonf significance
NPTrustApp <- aggregate(Trust ~ Source, data = THData1, FUN = median)
NPTrustApp
#define new column to add
NPBonSigTrust <- c('a','b','c', 'd', 'ad', 'd')
#add column called 'new'
NPTrustSig <- cbind(NPTrustApp, NPBonSigTrust)
#view new data frame
NPTrustSig
```
